# Crystal Clear: Metal–Organic Frameworks Pioneering the Path to Future Drug Detox

**DOI:** 10.1021/acsami.4c02450

**Published:** 2024-05-30

**Authors:** Przemysław J. Jodłowski, Klaudia Dymek, Grzegorz Kurowski, Kornelia Hyjek, Anna Boguszewska-Czubara, Barbara Budzyńska, Weronika Mrozek, Norbert Skoczylas, Łukasz Kuterasiński, Witold Piskorz, Marek Białoruski, Roman J. Jędrzejczyk, Piotr Jeleń, Maciej Sitarz

**Affiliations:** † Faculty of Chemical Engineering and Technology, Cracow University of Technology, Warszawska 24, Kraków 31-155, Poland; ‡ Lukasiewicz Research Network − Krakow Institute of Technology, Zakopiańska 73, Kraków 30-418, Poland; ¶ Department of Medical Chemistry, Medical University of Lublin, Chodzki 4A, Lublin 20-093, Poland; § Independent Laboratory of Behavioral Studies, Medical University of Lublin, Chodzki 4A, Lublin 20-093, Poland; ∥ Faculty of Geology, Geophysics and Environmental Protection, AGH University of Krakow, Mickiewicza 30, Kraków 30-059, Poland; ⊥ Jerzy Haber Institute of Catalysis and Surface Chemistry, Polish Academy of Sciences, Niezapominajek 8, Kraków 30-239, Poland; # Faculty of Chemistry, Jagiellonian University in Kraków, Gronostajowa 2, Kraków 30-387, Poland; ○ Małopolska Centre of Biotechnology, Jagiellonian University in Kraków, Gronostajowa 7A, Kraków 30-387, Poland; △ Faculty of Materials Science and Ceramics, AGH University of Krakow, Mickiewicza 30, Kraków 30-059, Poland

**Keywords:** metal−organic frameworks, amphetamine, MDMA, cocaine, in vivo

## Abstract

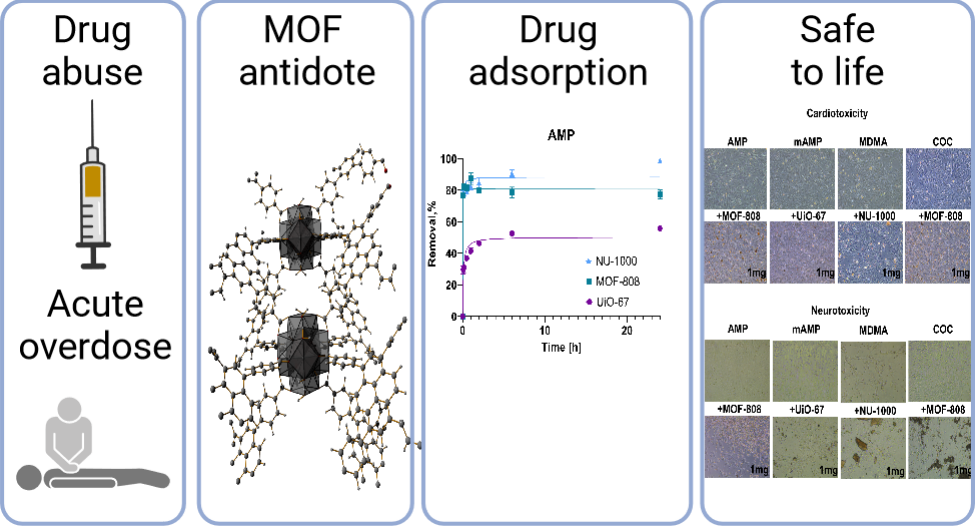

The growing number of acute drug abuse overdoses demands the development of innovative detoxification strategies for emergency purposes. In this study, an innovative approach for the application of porous Zr-based metal–organic frameworks for the treatment of acute overdoses of popular drugs of abuse including amphetamine, methamphetamine, cocaine, and MDMA is presented. A comprehensive approach determining the efficacy and the kinetics of drug removal, considering dosage, adsorption time, and adsorption mechanisms, was tested and corroborated with density functional theory (DFT) modeling. The experimental results showed high removal efficiency reaching up to 90% in the case of the application of the NU-1000 metal–organic framework. The difference Raman spectroscopy method presented in this study corroborated with DFT-based vibrational analysis allows the detection of drug adsorbed in the MOF framework even with as low a concentration as 5 mg/g. Additionally, the drug adsorption mechanisms were modeled with DFT, showing the π–π stacking in a vast majority of considered cases. The performance and influence on the living organisms were evaluated throughout the *in vitro* and *in vivo* experiments, indicating that Zr-based MOFs could serve as efficient, organic, safe drug adsorbents.

## Introduction

According to the World Health Organization (WHO) report,^1^ one in every 17 people around the world abused drugs in 2021. Compared to the decade earlier, 23% growth may be observed. The total number of individuals who use psychoactive drugs is estimated to be 296 million people aged 15–64.

**Figure 1:**
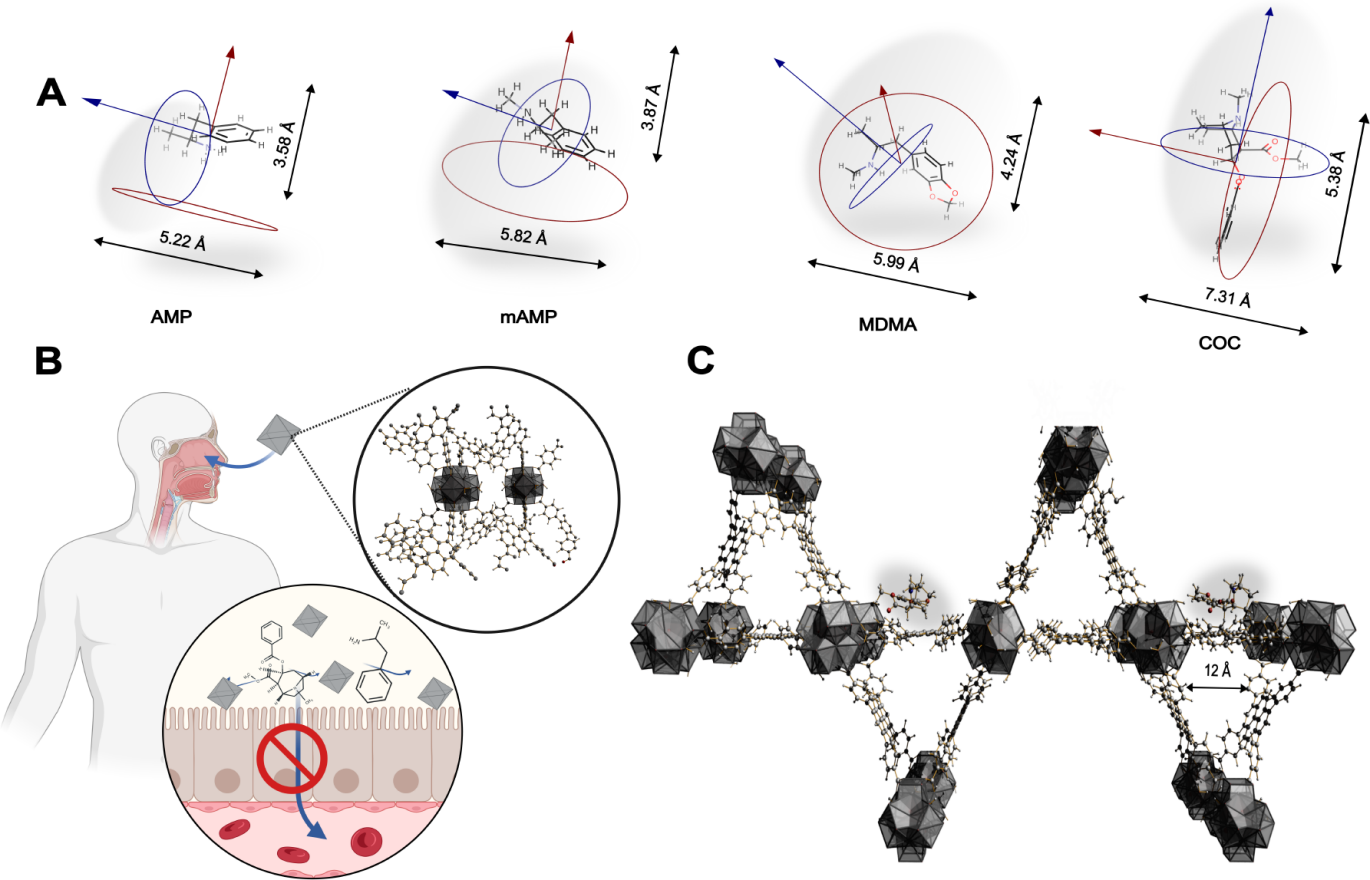
Chemical structures and geometrical projection descriptors of (A) amphetamine (AMP), methamphetamine (mAMP), 3,4-methylenedioxymethamphetamine (MDMA), and cocaine (COC); (B) perspective illustration of MOF application during the emergency detox (created with BioRender.com); (C) DFT-optimized structure of COC@NU-1000; adsorbed COC molecules marked as ellipsoids.

Drug addiction is categorized as a chronic relapsing mental disorder. According to the literature, this is characterized by an uncontrollable desire to take the substance despite awareness of its negative effects. Nowadays, medicine provides certain criteria to qualify a person as an addict. The list of criteria to evaluate a patient is called the Diagnostic and Statistical Manual of Mental Disorders, Fifth Edition (DSM-V). According to research, drug addiction develops through occasional use, recreational use, and regular use to addiction. Drugs of abuse directly or indirectly increase the release of dopamine and other neurotransmitters in the brain’s reward system. Neuroanatomically, the reward system in the brain is a complex network of structures and pathways that play a crucial role in motivation, pleasure, and reinforcement of certain behaviors.^2,3^ The mesolimbic pathway connecting the ventral tegmental area (VTA) with the nucleus accumbens and the mesocortical pathway projecting from the VTA to the prefrontal cortex are mainly involved in rewarding processes.^4^ Among the numerous psychoactive substances, amphetamine (AMP), methamphetamine (mAMP), 3,4-methylenedioxymethamphetamine (MDMA), also known as ”ecstasy”, and cocaine (COC) are the most abused drugs. The first three are also characterized by a very similar structure (Figure 1 A).

AMP (1-methyl-2-phenethylamine), see Figure 1A, is the world’s best-known illicit psychoactive central nervous system (CNS) stimulant and was synthesized as early as 1887.^5^ Its molecular weight is relatively small. As a stimulant, it causes an acceleration of heartbeat and breathing, a reduction in appetite, an increase in sexual desire, dry mouth, a feeling of exhilaration, and an enhancement of self-confidence. Even so, its intake’s effect varies depending on the dose taken, weight, height, health condition, or correlation with other drugs.^6,7^ AMP has gained popularity not only among young people but also among the elderly. Remarkably, the U.S. Food and Drug Administration (FDA) has approved AMP treatment for two diseases, specifically attention-deficit/hyperactivity disorder (ADHD) and narcolepsy.^7^ Only marijuana is a more frequently taken illegal drug.^5^ Less than six years after the discovery of AMP, *N*-methyl-alpha-methylphenethylamine, referred to as mAMP (Figure 1A) was synthesized. Although mAMP is also classified as a stimulant, its effects are significantly stronger than those of AMP. However, the effects are similar and include hypertension, sleep problems, headaches, anxiety, arrhythmia, paranoia, loss of appetite, and aggression.^8,9^ The force and nature of the effects of mAMP are reflected in the so-called postmethamphetamine psychosis. This is a state of hallucinations, mood disturbance, or irritability occurring even up to several days after taking mAMP.^10^ Another substance from the amphetamine group is MDMA (Figure 1A), which is often referred to as a “party drug” due to its psychoactive properties, such as feelings of euphoria and increased energy, which can be attractive in party and club settings. However, it is important to note that MDMA can also induce dangerous side effects, such as elevated blood pressure, panic attacks, loss of consciousness, and fainting. These adverse effects can occur, especially when the substance is abused, in the absence of proper hydration, and when it is taken without control. A popular street drug “ecstasy” often contains MDMA as one of its primary psychoactive ingredients. MDMA is compared to 4-methylmethcathinone (mephedrone) in terms of the pleasure felt after taking it, its potency, and its effects.^11^ All the drugs mentioned are classified as synthetic, psychostimulant substances. In contrast, COC (Figure 1A) is a natural plant alkaloid first extracted from *Erythroxylon coca* leaves in 1890. This compound contributes to an increase in dopamine and norepinephrine levels in the central and peripheral nervous system. It is abused due to its ability to induce intense euphoria, increase energy, and enhance sociability. This illicit use carries significant health and legal risks including myocarditis, arrhythmia, hypertension, or heart failure.^12,13^ When considering the way of administration of the above-considered drugs, it may be found that AMP, mAMP, and COC are mostly abused via oral administration, injection, or snorting, whereas in the case of MDMA, oral administration and snorting are mostly practiced. The latter is abused in the form of powder, colloquially called “molly” and is characterized by rapid and intense action usually accompanied by colored visual effects.^14^

Although a highly popular nasal administration of AMP, mAMP, COC, and MDMA, intensifies euphoria, it also intensifies side effects like increased withdrawal symptoms, increased body temperature, dehydration, nausea, and issues related to nasal administration like nosebleeds, permanent damage to the nasal septum, liver or kidney failure, or even death. Apart from the common acute overdose effects of the drugs from the amphetamine group, several additional factors that may amplify the overdose effect that may lead drug abusers to death should be considered, including additional drug contamination or even the substitution of other compounds with similar effects. The leading example is the contamination or even substitution of MDMA, with paramethoxyamphenimine (PMA) and paramethoxymethamphetamine (PMMA). Since both molecules have similar effects to MDMA, their action is delayed in comparison with MDMA, and as a result, drug abusers take additional drug portions.^15^ In consequence, both increased serotonin release accompanied by its blocked breakdown lead to serotonin syndrome and finally to seizures or death. It should not be overlooked that the study in conditions analogous to nasal administration, more realistic than those described in the already published articles, is a novelty in this article.

Bearing in mind the increase in people addicted to substances such as AMP, mAMP, MDMA, and COC, and the high popularity of the stimulants mentioned above, it is necessary to find novel alternative ways to detoxify patients with acute drug overdose. Currently, depending on the drug administration (oral, nasal, intravenous), medicine utilizes the methods of cleansing the body by using, e.g., gastric lavage, or administering substances that act antagonistically to the overdosed compound or hemodialysis or continuous renal replacement therapy.^16,17^

Despite the problems with overdoses, there is no fully effective treatment. Notably, there is a lack of effective sorbents for toxins, including drugs, that could be administered on-site without delay, even by nonspecialists in toxicology, such as paramedics or family members. Given the growing and ongoing problem and the large gap in this area of medicine, we propose the idea of using metal–organic frameworks (MOFs). MOFs are a new group of highly porous materials. Considering their structure, namely the inorganic metallic part and the organic linker, it can be found that they have many unique features.^18−20^ Characterized by high specific surface areas, high thermal and chemical stability, and through this, a variety of application possibilities. Low toxicity, high bioavailability, or biocompatibility enable the biomedical applications of MOFs. The structures have been proven as carriers of many drugs.^21−23^ The release of many therapeutic substances from MOFs has been described, including 5-fluorouracil (5-FU^24^), a well-known chemotherapeutic agent, chloroquine (CQ^25^), or acriflavine (ACF^26^), which has shown potential against the SARS-CoV-2 virus. The paper by Jodłowski et al.^25^ presents the protective effect of the MOF on the molecule of the released drug, as well as the reduction in drug toxicity after administration and its gradual release. In the case of the adsorption efficiency of MOFs, their potential use for pharmaceutical water purification has also been proven.^27−29^ Additionally, the use of MOFs in the adsorption of hippuric acid and 3-indoleacetic acid has been reported to imitate an artificial kidney.^30^ Likewise, mephedrone sorption by MOF networks has been described.^31^ High sorption capacity is yet another advantageous property. Combined with biomedical applications, it is conceivable and justifiable to use them as drug adsorbents during intentional or unintentional overdose. In the work of Rojas et al.,^32^ the application of MIL-127 composed of Fe^3+^ and 3,3′,5,5′-azobenzenetetracarboxylate as a biocompatible material for the treatment of poisoning or accidental oral intoxication by acetylsalicylic acid (ASA) as a model overdose molecule was proposed. As reported, the use of MIL-127 considerably decreases salicylate concentration in the gastrointestinal environment (GI), by reducing its concentration even 40-fold, possessing high MOF structural stability at the same time. It is also worth mentioning that the *in vivo* studies have confirmed MIL-127’s structure stability along the GI track and its further excretion in faeces.

In other work of Rojas et al.,^33^ MIL-125-NH_2_ (Ti-nanoMOF) was used as an efficient adsorbent of ASA. Analogously, the authors have confirmed the high biocompatibility and high efficacy of MIL-127-NH_2_ as an oral detoxification agent. It is also worth mentioning that similarly in this particular MOF, its chemical resistance to the GI environment was also confirmed. Nonetheless, MIL-127-NH_2_ was revealed to have a considerable salicylate protective effect.

Focusing on the capabilities of these materials as well as drug abuse problems, MOFs were chosen as selective adsorbents during overdoses. In our study, three types of Zr-based MOFs were synthesized and characterized, more specifically: MOF-808, UiO-67, and NU-1000. The structures applied differ in the ligand used and thus in the pore sizes and properties. The use of Zr-MOF networks as drug adsorbents during overdose has been proposed, referring to the real problem of drug abuse in the modern world.

This paper aims to broaden the concept of utilizing Zr-MOFs as effective adsorbents used *in situ*, i.e., with the nasal method of administration for both the drug and MOFs (Figure 1B), which is complementary to already published methods (oral) for commonly abused drugs such as AMP, mAMP, MDMA, and COC. These drugs possess different molecular properties that influence the proposed method of drug adsorption via MOFs. The drug removal method proposed in this article, based on its efficient adsorption, may be engineered via a proper MOF structure and specific interaction between host–guest molecules and meets the expectations of modern toxicology for the development of biocompatible adsorbents with low cytotoxicity. More importantly, the current state of the art regarding the application of MOFs to the efficient removal of drugs of abuse is scarce, mainly due to the difficulty of legally purchasing these substances, which require permission from government agencies. As a result, the mechanisms responsible for the efficient adsorption of popular drugs over the MOFs remain unknown.

Thus, in this study, a comprehensive approach to understanding the mechanisms of the efficient adsorption of AMP, mAMP, MDMA, and COC is presented. The adsorptive removal of popular drugs is complementary to the treatment described by experimental and theoretical approaches. Furthermore, keeping in mind the prospective application of MOFs in detoxification treatment, the effectiveness and low cytotoxicity properties of MOFs were corroborated by both *in vivo* and *in vitro* studies. Also, we assessed the impact of these compounds on toxicity as well as the peripheral and central effects of psychoactive substances in the larval model of *Danio rerio*.

## Materials and Methods

### Material Synthesis and Characterization

The Zr-based MOFs including UiO-67,^34^ MOF-808,^35^ and NU-1000,^36^ were synthesized according to the literature. The Supporting Information (SI) summarizes the detailed synthesis parameters.

The synthesized parent MOFs as well as MOFs after the adsorption of drugs of abuse were characterized by powder X-ray Diffraction (PXRD), low-temperature nitrogen adsorption, scanning electron microscopy (SEM), and μRaman spectroscopy. The characterization details are summarized in the Supporting Information.

Based on the literature structures (Table S1), the computational models were optimized at the periodic DFT+D level of theory. The details of the computational methodology are summarized in the Supporting Information, which also describes the structural and electronic properties and the Raman spectra simulations.

### Drug of Abuse Adsorption Studies

The adsorption efficiency of selected drugs of abuse, including AMP, mAMP, MDMA, and COC, was determined by the procedure previously reported for mephedrone,^31^ with some modifications. The kinetic tests for AMP, mAMP, MDMA, and COC adsorption involved exposing 10 mg of previously activated MOF samples to 2 mL of 500 μM drug aqueous solutions (AMP, mAMP, MDMA, or COC) under constant temperature conditions (25 °C). The concentrations of drugs of abuse were measured at specific time intervals by collecting 0.1 mL samples. These collected samples were then appropriately diluted, centrifuged at 6000 rpm for 5 min, and analyzed using the HPLC methods. Additional adsorption experiments at the *in situ* conditions simulating the nasal environment were performed in simulated nasal fluid (SNF).^37^ Prior to the adsorption experiment, simulated nasal fluid was performed by dissolving 8.77 g NaCl, 2.98 g KCl, and 0.59 g CaCl_2_ per liter of doubly distilled water.^37^ The pH was adjusted to 6.3 at 22 °C by the addition of hydrochloric acid. The adsorption efficiency was determined according to the procedure analogously to those described above, with the difference that adsorption was performed from the 500 μM drug SNF solutions (AMP, mAMP, MDMA, or COC) under constant temperature conditions (25 °C) that correspond to the temperature in the nasal cavity.^38^

Chromatographic separation of substances used Nexer XR LC-20AD with Shimadzu LCMS-2020 (AMP and mAMP), RID-20A (MDMA), and RF-20A XS (COC) detectors. The Knauer column (150 × 4.6 mm^2^, C18, 5 μm) was chosen for the separation. Two phases were prepared for the chromatographic separation of AMP and mAMP: 0.1% aqueous acetic acid solution (eluent A) and acetonitrile (eluent B). Isocratic flow (80% B) was used until 2.00 min, and then a gradient was used to obtain 100% eluent B at 4.20 min. 100% eluent B concentration was maintained until 4.40 min, and a linear gradient was used to obtain 80% eluent B from 10.00 min. Isocratic flow (80% B) was maintained for up to 10 min. The injection volume was 10 μL and the eluent flow was 0.6 mL/min. The column was thermostated at 30 °C. Mass spectrometry analysis was performed in the single reaction monitoring mode, measuring the proton fragmentation product (*m*/*z* = 119).^39^

For the separation of MDMA, two phases were prepared: eluent A composed of 5% 0.1 M solution of ammonium acetate in acetonitrile (ACN), 5% methanol MeOH, and 90% demineralized water, and eluent B composed of 45% ACN, 45% MeOH, and 10% demineralized water. Isocratic flow (100% A) was used until 6.00 min, and then, a gradient was used to obtain 70% eluent B at 30.00 min. The injection volume was 0.5 μL and the eluent flow was 0.5 mL/min. The column was thermostated at 40 °C. The excitation and emission wavelengths of the fluorescence detector were 288 and 324 nm, respectively.^40^

For the separation of COC, two phases were prepared: 0.01 mM aqueous ammonium acetate solution (eluent A) and ACN (eluent B). Isocratic flow (78% A) was used until 10.00 min, and then, a gradient was used to obtain 40% eluent B at 13.00 min. The 40% concentration of eluent B was maintained until 15.00 min. The injection volume was 40 μL and the eluent flow was 1 mL/min. The column was thermostated at 40 °C. Results were collected at a wavelength of 239 nm.^41^

The additional reusability studies were performed for the adsorption of COC from the SNF solution using NU-1000. The experiment of reusability of NU-1000 was performed by adding 10 mg of previously activated MOF sample to 2 mL of a 500 μM drug solution in SNF for 6 h under controlled temperature conditions (25 °C). Subsequently, spent NU-1000 was collected from the spent COC SNF solution and regenerated in methanol for 1 h under vigorous stirring. Subsequently, the regenerated MOF sample was activated using the standard activation procedure described above. The reusability was repeated 3 times. The concentration of COC was determined using the protocol described above.

The MOF adsorption selectivity tests were performed from SNF solution containing 500 μM MDMA and 500 μM COC. During the adsorption selectivity experiments, 10 mg of previously activated MOF sample was added to 2 mL of drug solution containing 500 μM MDMA and 500 μM COC in SNF for 24 h under controlled temperature conditions (25 °C). Subsequently, the spent MOF sample was collected from the spent SNF solution and the concentration of MDMA and COC was determined as described above for single drugs adsorption experiments.

The stability and safety for the nasal treatment of prepared materials were determined in terms of the release of the organic linker and Zr from the MOF matrix in the SNF medium. To test the MOF stability, 10 mg of a previously activated MOF sample was placed in 10 mL of SNF solution and left overnight at 25 °C under vigorous stirring. Subsequently, the resultant suspension was centrifuged at 6000 rpm for 5 min. The collected solution was then analyzed by UV–vis and X-ray fluorescence (XRF) spectroscopy methods to determine the concentrations of released organic linkers and Zr in the SNF solution. The crystal structure of the MOF sample after the soaking test was determined using the PXRD method. To determine the organic linker release, 1 mg of H_4_TBAPy (NU-1000), bpdc (1,1′-biphenyl-4,4′-dicarboxylic acid, UiO-67), and 1,3,5-benzenetricarboxylic acid (MOF-808) were dissolved in 10 mL of DMF/water (1:1) solution. The organic linker concentration was measured by monitoring maximum absorption intensities at 304, 280, and 280 nm for H_4_TBAPy (NU-1000), 1,1′-biphenyl-4,4′-dicarboxylic acid (UiO-67) and 1,3,5-benzenetricarboxylic acid (MOF-808), respectively. The amount of organic linker release was calculated from calibration curves using a Thermo Evolution 220 UV–vis spectrometer. The percentage of MOF degradation in the SNF solution was related to the organic linker concentration after dissolving 20 mg of pristine MOF samples in 35 mL of 1 M NaOH under ultrasonic irradiation. The determination of the zirconium release from MOF structures to SNF medium was determined by the XRF method using Bruker Tracer III-SD X-ray fluorescence spectrometer by dissolving ZrOCl_2_ × 8H_2_O in distilled water. The standard calibration curve determined the amount of Zr in the SNF solutions by integrating the Zr Kα line in the 779 channel (15.78 keV).

### *In Vitro* and *In Vivo* Experiments

Feline astrocytes (PG-4, ATCC: CRL-2032) and rat cardiomyoblasts (H9C2, ATCC: CRL-1446) were employed to assess the cytotoxicity of the MOFs (UiO-67, MOF-808, and NU-1000). Selected cell lines can serve as appropriate and reliable models for studying the toxicity of compounds in organs and tissues under *in vitro* conditions. They were feline astrocytes (PG-4, ATCC: CRL-2032) and rat cardiomyoblasts (H9C2, ATCC: CRL-1446) due to their relevance to the nervous system and heart tissue, which were key issues in toxicity studies of selected MOFs.

Neuronal cells cultured directly from brain tissue provide insights into cellular responses after the application of tested substances. PG-4 (S+L−) is a glial, astrocyte cell line isolated in 1980 from the brain of a normal embryo, deposited by KJ Dunn, and may be used in neuroscience research. Astrocytes are a type of glial cell in the brain and spinal cord, having many important functions, including drug addiction and neural adaptations after exposure to drugs of abuse. Recent results have identified several key astrocytic signaling pathways that are involved in cocaine-induced synaptic and circuit adaptations.^42^

Myoblasts are precursor cells to muscle cells, including cardiac muscle cells (cardiomyocytes). Therefore, they share some characteristics with cardiomyocytes and can provide insights into potential toxic effects on cardiac tissue. H9C2(2–1) is a subclone of the original clonal cell line derived from embryonic BD1X rat heart tissue that exhibits many skeletal muscle properties. This cell line can be used in cardiovascular disease research. Myoblasts, including H9C2 cells, have been shown to exhibit sensitivity to various cardiotoxic compounds, including drugs and environmental toxins. Therefore, they can be used to assess the potential cardiotoxic effects of new compounds or materials. The use of recreational drugs, including new psychoactive substances (NPS), is paralleled by emergency department visits of drug users with severe cardiotoxicity.^43^

In the cytotoxicity examination, the selected Feline astrocytes (PG-4, ATCC: CRL-2032) and rat cardiomyoblasts (H9C2, ATCC: CRL-1446) were examined in conjunction with the following substances: AMP, COC, mAMP and MDMA. PG-4 cells were cultured in McCoy’s Medium, supplemented with 10% Fetal Bovine Serum (FBS), 100 U/mL penicillin, and 100 μg/mL streptomycin, while the cardio myoblasts were cultured in Dulbecco’s Modified Eagle’s Medium (DMEM), also supplemented with 10% FBS, 100 U/mL penicillin, and 100 μg/mL streptomycin. Both cell lines were maintained at 37 °C in a humidified atmosphere containing 5% CO_2_. Subculturing and feeding occurred every 3 days to prevent cell differentiation.

For the experiments, cells between passages 7 and 20 were utilized. Initially, cells were seeded at a concentration of 5 × 10^5^ cells/mL in 96-well plates and incubated for 24 h. Subsequently, the cells were exposed to drugs at concentrations ranging from 0 to 500 μg/mL. Cell viability was assessed using the 3-(4,5-dimethylthiazol-2-yl)-2,5-diphenyltetrazolium bromide (MTT) assay, and IC50 values were determined for AMP, mAMP, MDMA, and COC.

In the next phase of the experiment, the toxicity of the MOFs was evaluated. Cells were seeded at a concentration of 5 × 10^5^ cells/mL in 6-well plates and incubated for 24 h. Afterward, the cells were exposed to MOFs at a concentration of 2 mg/mL. Cell viability was visually assessed after 24 and 48 h.

Subsequently, cells were exposed to drugs and a combination of drugs with the MOFs. Cells were seeded at a concentration of 5 × 10^5^ cells/mL in 24-well plates and incubated for 24 h. One group of cells was exposed to drugs at a concentration of 250 μg/mL, while another group received drugs with the addition of MOFs at a concentration of 1 mg/mL. Cell viability was visually assessed after 24 and 48 h.

Microscopic images were captured by using a Leica DMi1 microscope.

#### *In Vivo* Experiments

##### Animals

*Danio rerio* (zebrafish) specimens, of AB strain, were kept at the Experimental Medicine Center, Medical University of Lublin, Poland, at a temperature of 28.5 °C. They followed a light/dark cycle of 14 h of light and 10 h of darkness as part of standard aquaculture practices. Fertilized eggs were obtained through natural spawning. The embryos were raised in an E3 embryo medium (with a pH range of 7.1–7.3), which consisted of 17.4 μM NaCl, 0.21 μM KCl, 0.12 μM MgSO_4_, and 0.18 μM Ca(NO_3_)_2_. The incubator maintained a temperature of 28.5 °C for the embryos. The larvae were euthanized by immersing them in a solution containing 15 μM tricaine. All details were described in our previous work.^26^

##### Fish Embryo Toxicity (FET) Test

As its foundation, the zebrafish embryo test utilized the adapted OECD Guidelines for the Examination of Chemicals (OECD, 2013). Within 90 min after fertilization, embryos were meticulously examined using a light microscope (Stemi 508, Zeiss). Subsequently, viable and fertilized embryos were relocated to 96-well plates within 3 h post fertilization (hpf). Each embryo was placed separately in 200 μL of the substances being tested or control solutions. Over the course of 96 h, the embryos were subjected to these “treatment”, or “control”, solutions.

At intervals of 24 h, the embryos were observed under a stereomicroscope, and data on their survival rate, hatching, and any irregularities in development were meticulously documented. Upon reaching 96 hpf, after letting the larvae acclimate to room temperature for half an hour, their heartbeats were tallied using a stereomicroscope for 15 s. The resultant values were multiplied by four to derive the heart rate in beats per minute (bpm). For the experiment, 12 larvae pretreatment were taken.

##### Locomotor Activity

For evaluation of locomotor activity, the assay was performed in 5 dpf larvae, after the FET test, with one larva in each well of a 96 multiwell plate. EthoVision XT video tracking software (Noldus) was used for evaluating locomotor activity. The distance moved in 10 min was calculated in a light condition. For the experiment, 12 larvae pretreatment were taken.

### Statistical Analysis

The statistical analysis for drug adsorption kinetics was performed using GraphPad Prism 10 software. Data were analyzed using one-way and two-way analysis of variance followed by Tukey’s posthoc test or Bonferroni’s posthoc test, respectively. The confidence limit of *p* < 0.05 was considered statistically significant. Grubbs’ test was employed as a statistical method to identify outliers at the significance level of 0.05.

All experiments were carried out following the National Institute of Health Guidelines for the Care and Use of Laboratory Animals and the European Community Council Directive for the Care and Use of Laboratory Animals of 22 September 22, 2010 (2010/63/EU). For the experiment with larvae up to 120 hpf, the agreement of the Local Ethical Commission is not required.

## Results and Discussion

### Synthesis and Characterization of Zr-Based MOFs As an Efficient Drug of Abuse Adsorbents

The crystallinity of Zr-MOF used in this study was determined using PXRD analysis (Figure S1A).

The obtained PXRD patterns confirm the high crystallinity of prepared samples and remain in good agreement with the literature data.^26^

The pore structure of parent MOFs was determined by using a low-temperature gas adsorption method. Differing adsorption isotherms were obtained, indicating the different pore structures of these materials (Figure S1B, C). Based on these isotherms, structural parameters were calculated (Table 1).

**Table 1:** Sample Characteristics

Sample	*S*_BET_ (m^2^/ g)	*S*_Lang_ (m^2^/ g)	*V*_micro_ (cm^3^/ g)	*D* (Å)^a^	*n*_molecules_, DFT calcd^b^
**MOF-808**					
pristine	1340.4	2115.2	0.714	34.9	–
AMP	870.6	1418.4	0.475	31.2	24 (6)
mAMP	929.0	1516.8	0.509	29.2	21 (5.25)
COC	844.6	1382.9	0.460	33.6	12 (3)
MDMA	962.3	1507.0	0.504	31.7	10 (2.5)
**UiO-67**					
pristine	2405.8	3306.6	1.203	19.9	–
AMP	7.5	12.5	0.005	65.2	9 (9)
mAMP	12.2	22.8	0.007	59.3	7 (7)
COC	24.3	40.7	0.014	47.2	6 (6)
MDMA	15.6	25.0	0.009	57.4	3 (3)
**NU-1000**					
pristine	1958.5	2660.7	1.249	27.0	–
AMP	978.6	1275.6	0.637	37.8	55 (18.33)
mAMP	448.3	587.3	0.291	38.1	38 (12.67)
COC	1317.7	1788.1	0.842	35.9	38 (12.67)
MDMA	594.8	779.0	0.381	35.8	23 (7.67)

a Average pore diameter calculated from the BET model.

b Calculated within the rigid host approximation. Values in parentheses–per single Zr_6_ cluster.

For the N_2_ adsorption in MOF-808, an isotherm typical of mesoporous materials was obtained (type II), where the filling of the surface with adsorbate occurs in multilayers (Figure S1B). Here, the BET surface area was 1340.4 m^2^/g, and an increased pore volume below 1 nm and 1.5–2 nm and few pores above 2.5 nm in diameter were observed (Figure S1C). In UiO-67, the obtained isotherm was of the type I – typical for microporous materials, where the surface filling is a monolayer. This was reflected in the developed BET and Langmuir surface area, which amounted here to 2405.6 and 3306.6 m^2^/g, respectively. In this material, the highest volume was recorded in fine micropores up to 1.5 nm in diameter. In NU-1000, a type IV isotherm was determined and, in accordance with this, increased volume was observed in pores with two diameter ranges, mainly up to 1.5 nm and from 2.5 to 4 nm. The recorded BET surface area here was 1958.5 m^2^/g (Figure S1B, C).

The morphology of MOF samples after the adsorption of AMP, mAMP, COC, and MDMA was determined by using scanning electron microscopy. The results are shown in Figures S2–S6, respectively.

The morphology of MOF samples after the adsorption of AMP, mAMP, COC, and MDMA does not differ significantly from that of parent MOF samples (Figures S2–S6). The crystals of MOF-808 were detected as spherical particles with an average crystal size of ca. 50 nm. In the case of the parent UiO-67 sample, the crystal size may be considered to have the largest crystal size around 1 μm with a truncated octahedral morphology. The parent NU-1000 sample revealed uniform rice-grain-shaped crystals with an average length of ca. 200 nm. In all considered samples MOF crystal morphologies are in good agreement with the literature data.^44−46^ It is worth mentioning that in the case of samples after the adsorption of AMP, mAMP, COC, and MDMA, the individual crystals appear to be stuck together. This phenomenon is most visible in the case of the adsorption of mAMP over MOF-808 and NU-1000 samples (cf. Figures S2 and S4). It must be pointed out that the reason for the appearance of stuck MOF crystals may be due to the fact that the MOF samples after the adsorption were only centrifuged without further pretreatment before the SEM analysis.

### Raman Characterization

**Figure 2:**
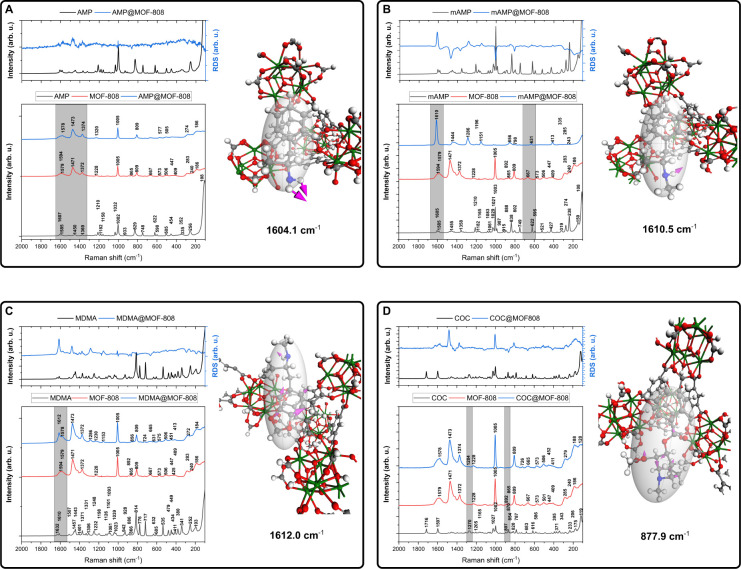
μRaman difference spectroscopy results, RDS, (upper plots) and μRaman analyses (lower plots) of MOF-808 after adsorption of (A) AMP, (B) mAMP, (C) MDMA, and (D) COC; DFT optimized structures with vibrational modes, normalized independently for each structure by the corresponding transition intensities, depicted as arrows. For clarity, some insignificant vectors have been omitted and also visible vectors have been rescaled to increase their visibility. N–H deformation vibration for (A, B, C), and C–C tropane ring stretching vibrations for (D).

To comprehensively characterize MOF materials after the adsorption of AMP, mAMP, COC, and MDMA, μRaman analyses were performed for pure drugs of abuse, parent MOF samples, and drug@MOF samples. The results of μRaman analyses, namely, the representative μ Raman spectra of pure AMP and mAMP, MDMA, COC, pristine MOFs, and MOF samples after the adsorption of AMP, mAMP, MDMA, and COC for MOF-808 are shown in Figure 2 and separately for all drugs of abuse adsorbed in UiO-67 and NU-1000 in Figures S7 and S8.

The obtained μRaman spectra of pristine MOFs are in good agreement with the literature data,^47,48^ which confirms a high crystallinity of prepared samples and MOF structure stability after the adsorption of drugs. At first glance, a comparison of the characteristic μRaman spectra of MOFs after the adsorption of drugs of abuse with μRaman spectra of pristine drugs shows no evidence of drugs adsorbed in the MOF structure. Since the characteristic Raman bands for pristine drugs (Table S3) and characteristic MOF vibrations (Figure 2, Figures S7 and S8) overlap in the most distinctive regions, the N–H or C–N vibrations are indicative of successful drug adsorption in most of the considered cases.

Due to the low initial concentrations of considered drugs and characteristic band overlapping on the Raman spectra, the Raman Difference Spectroscopy (RDS), developed to highlight the changes that occur relative to its ref (49), was applied to monitoring drug adsorption. By the subtraction of measured spectra (e.g., AMP@MOF-808) from the reference spectra (MOF-808), the changes in the spectrum are emphasized. The characteristic RDS values for all considered MOFs are summarized in Table 2.

**Table 2:** Raman Difference Spectroscopy (RDS) Results upon Sorption^a^

	**RDS band wavenumbers (cm^–1^)**			
**MOF**	**AMP**	**mAMP**	**MDMA**	**COC**
**MOF-808 exp.**	1603 (−4)	1613 (8)	1613 (3)	867^b^ (−3)
**MOF-808 calcd.**	1597 (9)	1597(13)	1617 (−5)	877 (1)
**UiO-67 exp.**	1614 (8)	1613 (8)	1612 (2)	1283 (4)
**UiO-67 calcd.**	1597 (11)	1596 (11)	1618 (−8)	1274 (0)
**NU-1000 exp.**	1606 (−1)	1603 (−4)	1606 (−4)	1277 (7)
**NU-1000 calcd.**	1608 (1)	1597 (−6)	1617 (−7)	1274 (−5)

a Values in parentheses: positive shift = blue-shift (increase in wavenumber), negative shift = red-shift.

b C–C stretching in a tropane ring.

It may be found that, in the case of AMP, mAMP, MDMA, and COC adsorption, the characteristic RDS bands originate from NH deformation vibrations of the amine group. The characteristic RDS bands for N–H deformation vibrations of the amine group vary around 1607 cm^–1^ for AMP, mAMP, and MDMA. In the case of COC, the band at 1270 cm^–1^, originating from the C–N stretching vibration, was used as an indicator RDS band. Even though that band was not evident in the case of the adsorption of COC@MOF-808 (broad weak band), the band at 867 cm^–1^ indicates the C–C vibrations in the tropane ring. In the case of UiO-67 and NU-1000, C–N stretching vibrations were observed at 1283 cm^–1^ and 1277 cm^–1^. It is worth mentioning that detection of the illicit drug of abuse may be challenging in the case of its efficient adsorption on MOFs. The presence of similar functional groups in both MOFs and considered drugs may overlap and the successful band assignment may be challenging. It is also worth mentioning that in the literature data methods such as the Surface Enhanced Raman Spectroscopy (SERS) or the Fourier Transform Infrared Spectroscopy (FTIR) are successfully applied to fast and efficient illicit drug detection^31,49^ even at very low concentrations, those methods are efficient when considering illicit drug in neat state. In our case, low-concentration illicit drugs are adsorbed in the MOF structures, and enhancing Raman signals by SERS would result in a simultaneous increase in the bands of both the MOF and the drug of abuse.

### Efficient Drug Adsorption–A Theoretical and Experimental Approach

**Figure 3:**
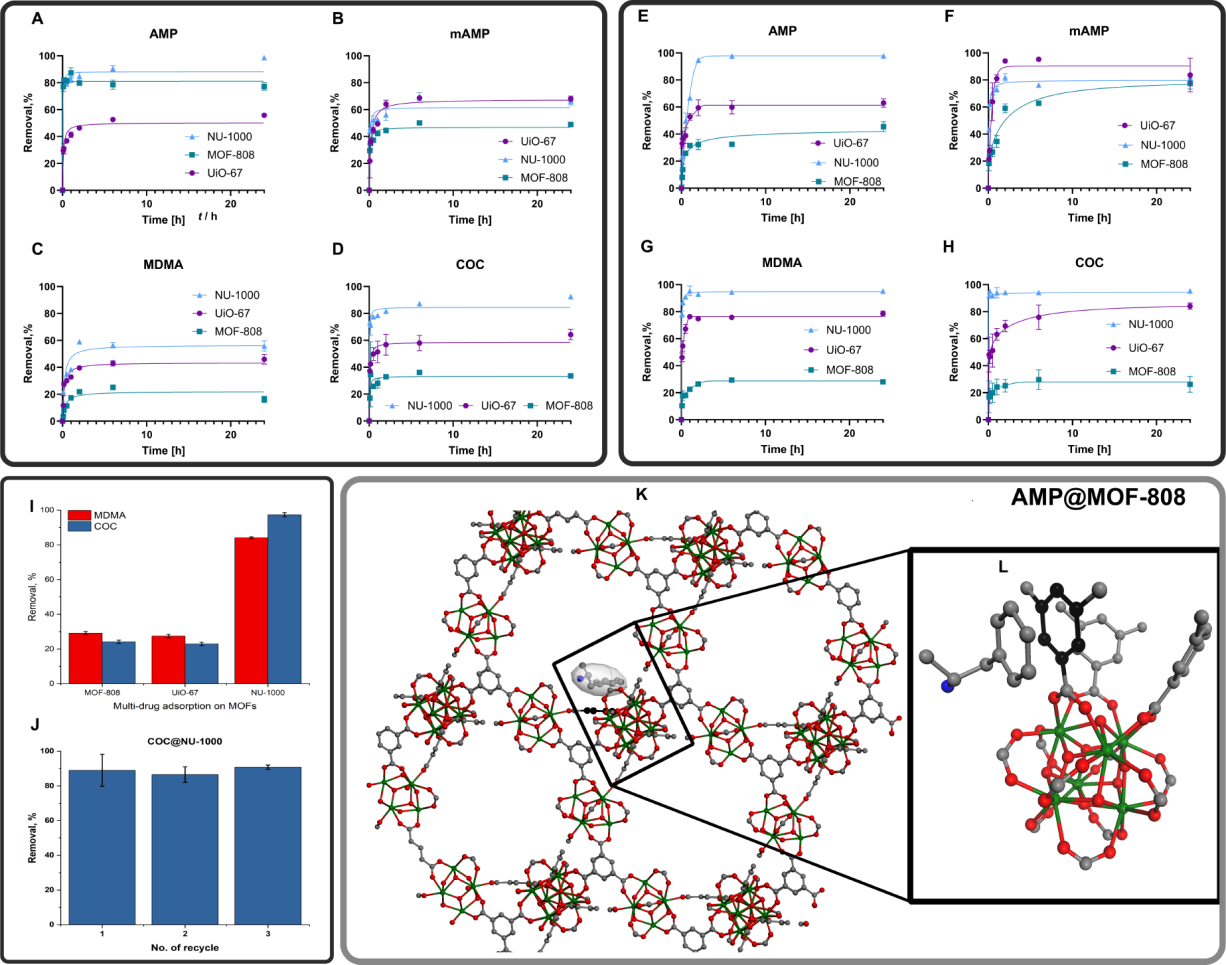
Removal of (A) AMP, (B) mAMP, (C) MDMA, and (D) COC over Zr-MOFs from water solution ; (E) AMP, (F) mAMP, (G) MDMA, and (H) COC over Zr-MOFs from SNF solution; (I) multidrug adsorption over Zr-MOFs, (J) MOFs reusability experiment results; Data (A–H) are presented as mean ± SD, *n* = 3; (K) DFT-optimized structure of AMP@MOF-808 adsorbed in channel; (L) close-up of adsorption mode. Color coding: gray: C, blue: N, red: O, green: Zr, black: an aromatic ring of the linker molecule involved in the π–π stacking. For the other adsorbate@MOF structures, see the Supporting Information.

The adsorption efficiency of AMP, mAMP, MDMA, and COC was determined over each of the MOF-808, UiO-67, and NU-1000. The results are summarized in the form of percentage removal of individuals as a function of time in Figure 3 (and *q*_*t*_ vs time, Figure S9), and in the form of pseudo-first and pseudo-second order kinetics in Figures S10–S11. Additionally, the adsorption experiments were performed in an SNF environment, and the results are shown in Figure 3 and Figure S12.

Additionally, the calculated pseudo-first-order and pseudo-second order kinetic parameters were summarized in Table S4. The adsorption kinetics for a series of drugs of abuse differs significantly depending on the drug adsorbate used and the MOFs. At first glance, it could be concluded that the adsorption of the drugs will be a derivative of the structural parameters of the considered drug and an average pore diameter of the MOF used in the adsorption process. The comparison of calculated geometrical descriptors for AMP, mAMP, MDMA, and COC shows that the parameters such as the maximal projection area, or the maximal projection radius or van der Waals volume of considered drugs of abuse, increase in the following order: AMP > mAMP > MDMA > COC. The general tendency to decrease the maximum sorption capacity was observed when comparing the percentage removal of selected drugs over zirconium-based MOFs (Figure 3). Indeed, for AMP, mAMP, and MDMA, the general decreased tendency in the percentage removal of drugs may be observed. However, for the geometrically bulkiest structure, COC, the decreasing percentage removal trend was not maintained, reaching the maximum percentage removal close to 80% for NU-1000, 60% for UiO-67, and 35% for MOF-808, respectively. It must be emphasized that, in the case of all drugs of abuse considered in this study, the most intense adsorption takes place in the first 2 h of the adsorption process. After 2 h of adsorption, the adsorption curves reach a plateau, and the adsorption reaches equilibrium. The analysis of the adsorption kinetics, expressed in mg/g of the MOF materials, indicates the undisputed leader in the removal of all considered drugs of abuse. Although the maximum adsorption capacity in the case of AMP was slightly higher when using UiO-67, after considering the calculated standard deviations, the adsorption efficiency can be considered equal for both UiO-67 and NU-1000. An interesting observation can be made when the adsorption curves for COC over all tested MOFs are analyzed (Figure 3D).

In all of the considered cases, the maximum adsorption capacity is doubled in all of the considered drugs of abuse. This fact is also expressed in the calculated equilibrium amount of adsorbed substance for pseudo-first and pseudo-second order (Table S4). The fact of the increasing the sorption capacity for COC over selected Zr-MOFs allows us to conclude that the geometric dimensions (Figure 1A) of both adsorbed molecules and pore dimensions are conditions sine qua non to adsorb drug molecules; the major impact on the adsorption of drug molecules and its strength, however, will be dominated by host–guest interactions.

The efficiency of the selected MOFs in the adsorption of AMP, mAMP, MDMA, and COC was determined in an SNF environment by simulating the in situ conditions (Figure 3). The general increasing drug adsorption tendency can be observed when comparing the adsorption efficiency results from SNF solution with those performed in a water environment. The most spectacular increase in the adsorption efficiency may be observed in the case of MDMA and COC for the NU-1000 sample, whereas in the case of MOF-808, only a slight increase was observed. It must be noticed that in the case of COC, overall drug adsorption in NU-1000 is almost achieved and an almost similar trend remained for MOF-808, whereas for UiO-67, the adsorption seems gradual and extended in time. It must be pointed out, that when considering drug adsorption in SNF solution, the adsorption environment is far more ion enriched when comparing the adsorption in a water environment.

Such behavior can be explained based on the hydrophobic nature of the aromatic/tropane rings, namely, when comparing the DFT-derived adsorption energy change upon an introduction of the polar solvent environment, which suggests that the rise of the ion concentration (and thus even more polar environment) can strengthen the sorption. The DFT calculations were performed to comprehensively describe the host–guest interactions between adsorbed drug molecules and the MOF framework.

In most cases, π–π stacking was found to be the main contribution to the adsorption energy. For the visualization of the DFT-optimized drug@MOF adsorbate structures, see Figure 3K, L for AMP@MOF-808 and Figures S26–S38 for the rest of the structures (in all cases, only a crucial part of the models is shown and the remaining parts of the structures are omitted for the sake of clarity) and Tables S6 and S7 for the energies, charge and bond analyses, and geometrical descriptors, respectively.

Indeed, for the molecules with an aromatic ring (AMP, mAMP, and MDMA), the value of *E*_ads_^sol^ – *E*_ads_^vac^ remains positive, in the range of 0.088 eV (AMP@MOF-808) to 0.121 eV (MDMA@MOF-808). For the heterocyclic tropane ring containing COC@MOF-808, a rise of as much as 0.267 eV is observed. For NU-1000, the values of *E*_ads_^sol^ – *E*_ads_^vac^ remain within the range of 0.009 eV (AMP@NU-1000) to 0.113 eV (COC@NU-1000).

The adsorption energies (in vacuum) remained in the range of −0.422 eV (AMP@NU-1000) to −0.935 eV (COC@UiO-67) for the systems with pronounced π–π stacking. When the polarizable continuum was introduced, mimicking the presence of the polar solvent environment, the sorption energies for the systems mentioned above rose slightly (i.e., their absolute values dropped to −0.413 eV and −0.871 eV, respectively), indicating that the drug molecules are not destabilized (and thus detached) by the solvent cavities. For the case where no π–π stacking is observed (COC@UiO-67-str3), the energetics change in the opposite direction upon the introduction of the solvent. Namely, for COC@UiO-67-str3, the adsorption energy drops from −0.966 eV to −1.012 eV, which can be interpreted as the trend to dissolve the COC molecule by water regardless of the relatively strong adsorption. Indeed, for COC@UiO-67-str3 the electronic (covalent) bond order is only 0.790, compared to 0.918 for COC@UiO-67-str1 which exhibits π–π stacking. The strongest covalent bonding (COC@MOF-808), 1.100, corresponds to the highest, to the absolute value, adsorption energy among all drug@MOF-808 structures. The same relationship holds for the other COC@MOF structures. The charge transfer between adsorbates and host frameworks was minute in all cases.

The μRaman spectra were interpreted by the DFT simulations via the harmonic vibrational analysis yielding the vibration eigenvalues (wavenumbers) and eigenvectors. For the modes visible in the experiment, the Raman intensities were calculated (see the SI section Vibrational analysis and Raman spectra for computational details and Figures S13–S24 for the visualization of the vibrational modes).

### MOF Stability in SNF Solution and Metabolic Pathways

To determine the stability of selected MOFs, a stability test was performed by soaking the MOFs in the SNF solution overnight. The structure of MOFs was then determined by PXRD (Figures S39 and S40) analysis. It may be found that in the case of MOF-808 and NU-1000, the crystalline structure remains unchanged, whereas the diffractogram of UiO-67 indicates its partial amorphization. As previously reported by Mondloch et al.,^50^ the UiO-67 is unstable in the water environment, due to the extended linker hydrolysis. However, it must be emphasized that, despite the structural collapse and partial amorphization of UiO-67, the adsorbed drug molecules were not back-released to either water or SNF solution. Additionally, the peaks originating from the NaCl (card no. 01–080–3939) and the KCl (card no. 01–074–9685) phases were detected in the PXRD diffractograms (Figures S39 and S40).

Additionally, for comparison, the structural parameters were also determined for MOFs after the adsorption of drugs of abuse (Table 1, Figures S41 and S42). In all metal–organic frameworks, drug adsorption caused significant changes in pore structure. The specific BET surface area in MOF-808 after the process decreased by about 30% and was about 840–960 m^2^/g and the available pore volume also reduced. The largest change in structure was obtained here after the COC and AMP treatment. In NU-1000, the structural parameters decreased significantly and more than half of the available pores were clogged. This was particularly evident in the MOFs treated with mAMP and MDMA, where the BET surface area was less than 600 m^2^/g and the total pore volume was 0.3 cm^3^/g. The greatest changes and collapse of the pore structure were observed in UiO-67. A significant decrease in structure parameters was observed in the UiO-67 sample, regardless of the type of drug used.

The stability tests were performed to further investigate the stability of selected MOFs in the SNF environment in terms of the release of the organic linker and Zr release to the SNF solution. The organic linker concentration in the SNF filtrates was measured by UV–vis (Figure S43), whereas Zr content was determined by XRF spectroscopy (Figure S44). In the UV–vis spectra of MOF-808 and NU-1000, the stability of the structure was confirmed by the absence of the organic linkers in the SNF filtrates, which is in line with the PXRD stability tests (TA LOD = 5.83 ppm, H_4_TBAPy LOD = 0.19 ppm) However, in the case of UiO-67, 35% MOF degradation was observed, which was indicated by an increased bpdc linker absorption intensity band (bpdc LOD = 0.19 ppm). Subsequently, the release of Zr from the MOF matrix was determined by XRF spectroscopy (Figure S44). It was found that in all considered MOFs, none of Zr was released to the SNF solution (Zr LOD = 10.19 ppm). Although the excellent hydrolytic stability of Zr_6_-based MOFs was previously reported in several works^50,51^ for the MOFs with similar linker lengths, it is worth mentioning that our results showing no Zr release to the SNF solution may indicate the partial structure defect generation through the SNF solution. It is also worth noting that the increased stability of MOFs is not limited to Zr_6_-based MOFs. In recent works of Rojas et al.,^32,33^ exceptional stability of MIL-125 in the gastrointestinal fluid (GI) was found. The MOF degradation in GI medium under acidic conditions (pH 1.2) was below 9%. On the other hand, in the case of MIL-127-NH_2_, ca. 30% MOF degradation was found, however, both Ti and organic ligand (H_2_BDC-NH_2_) were found to be removed by 95% in urine and only 0.03 and 0.02% were detected in the liver and spleen. Based on the stability results and bearing in mind the prospective application of MOFs for the treatment of the drug acute overdose the metabolic pathways of both nonadsorbed drug molecules and drug@MOFs should be considered. AMP metabolism primarily involves oxidative deamination and hydroxylation, mainly catalyzed by CYP2D6, leading to the formation of various metabolites such as hippuric acid or 4-hydroxyamphetamine.^52^ The stereoselective metabolism of amphetamine results in dextroamphetamine being metabolized more rapidly than levoamphetamine, resulting in differing half-lives and disproportionate concentrations of amphetamine excreted in urine. Genetic variability, particularly in CYP2D6 polymorphism, influences amphetamine metabolism, affecting its dose/effect relationship. Factors such as urinary pH, volume, age, weight, and diseases such as kidney failure significantly impact amphetamine excretion and half-life, with alkalinization leading to decreased excretion rates and vice versa. Understanding these metabolic pathways and their variability is essential for optimizing drug efficacy and minimizing adverse effects in clinical contexts. On the other hand, MDMA metabolism involves two primary pathways: *O*-demethylation followed by methylation or conjugation, and *N*-dealkylation leading to benzoic acid derivatives conjugated with glycine.^53^ The polymorphic enzyme CYP2D6 influences the *O*-demethylenation pathway, but its impact on acute toxicity is limited by mechanism-based inhibition after consecutive doses. However, MDMA metabolism may contribute to mid- to long-term neurotoxic effects through progressive neurodegeneration of the serotonergic neurotransmission system. In contrast, COC is primarily metabolized by plasma butyrylcholinesterase (BChE) to ecgonine methyl ester and benzoylecgonine.^54^ These metabolites may further hydrolyze into ecgonine. Additionally, cocaine can be metabolized to norcocaine by the cytochrome P450 enzyme CYP3A4. Other minor metabolites include norbenzoylecgonine (NBE), norecgonine methyl ester, and meta-hydroxybenzoylecgonine. Differences in BChE plasma levels can influence cocaine metabolism and alter its effects with lower BChE levels potentially associated with more adverse clinical outcomes. Similarly, the metabolic pathway of drug@MOF or more specifically, MOFs during the on-site acute overdose treatment and their biodistribution should also be considered. In the work of Baati et al.,^55^ the *in vivo* toxicity of three Fe-based MOFs including MIL-100, MIL-88A, and MIL-88B_4_CH_3_ differing in the organic linker (MIL-100- trimesic acid, MIL-88A- fumaric acid and MIL-88B_4_CH_3_- tetramethyl-terephthalic acid) was examined. The methodology of the *in vivo* toxicity on the animal model described the whole toxico-kinetics by following the series of experiments determining adsorption, distribution, metabolism, and elimination.^55^ In their work, the acute MOF cytotoxicity was determined in a group of rats by injecting high doses of different types of MOFs intravenously, keeping doses as high as 220 mg/kg, and the rats were examined 1, 7, and 30 days after the intravenous injection. The toxicity was evaluated in terms of animal behavior, histology, oxidative stress, metabolism, MOF biodistribution, and excretion. They found that the MOFs were degraded to iron and organic linkers and were removed by the organisms by urine and faces. The temporary iron increase in orgasms such as the spleen and liver resulted in utterly reversible oxidative stress. On the contrary, oral MOF administration and toxicity of MOFs were recently examined by Rojas et al.^32,33^ in a group of rats. In both MIL-127 and MIL-125-NH_2_ cases, MOFs were administered orally, and despite minor absorption of iron in the gastrointestinal tract, MOFs were removed directly via excretion in faces. Following the results presented in the literature,^32,33,55^ in the case considered in our work, the partial removal of MOFs could be nasal cleaning. In contrast, the majority of MOFs could be removed via the GI route and finally excreted in the faces. Keeping the MOF stability result from our study, the natural choice would be MOF-808 and NU-1000 whose high acidic stability was confirmed in numerous works.^56^ However, since the determination of metabolic pathways goes far beyond this review, we plan to extend our studies toward further detailed understanding. The selectivity of selected Zr-MOFs was tested in the adsorption of a mixture of MDMA+COC SNF solution (Figure 3 I). It was found that in all of the considered cases, the adsorption was limited mainly by the size of the drug molecule which is reflected especially for MOF-808 and UiO-67 samples. In the case of NU-1000, possessing relatively the largest pores system, the pores are almost utterly filled with MDMA molecules and subsequently by COC molecules, which has a reversed tendency compared to MOF-808 and UiO-67. Additionally, it may be observed that in the case of UiO-67, the overall adsorption efficiency is lower than in the case of single-drug adsorption from the SNF solution (Figure 3G and H). It may be found that the use of Zr-MOFs would be potentially beneficial when considering acute overdose in the case of patients abusing multiple drugs during drug trips.

The additional MOF reusability experiments were performed for NU-1000 for the adsorption of COC from SNF solution after 6h of the adsorption (Figure 3J). It was found that NU-1000 remains stable, and its sorption activity toward the COC from SNF solution remains at the same level equal to ca. 86%. Although in this study the application of selected MOFs for the removal of drugs of abuse during acute overdoses, their application for the removal of drugs of abuse from the aqueous solutions in other applications cannot be excluded as previously reported elsewhere.^31^

### In Vitro

**Figure 4:**
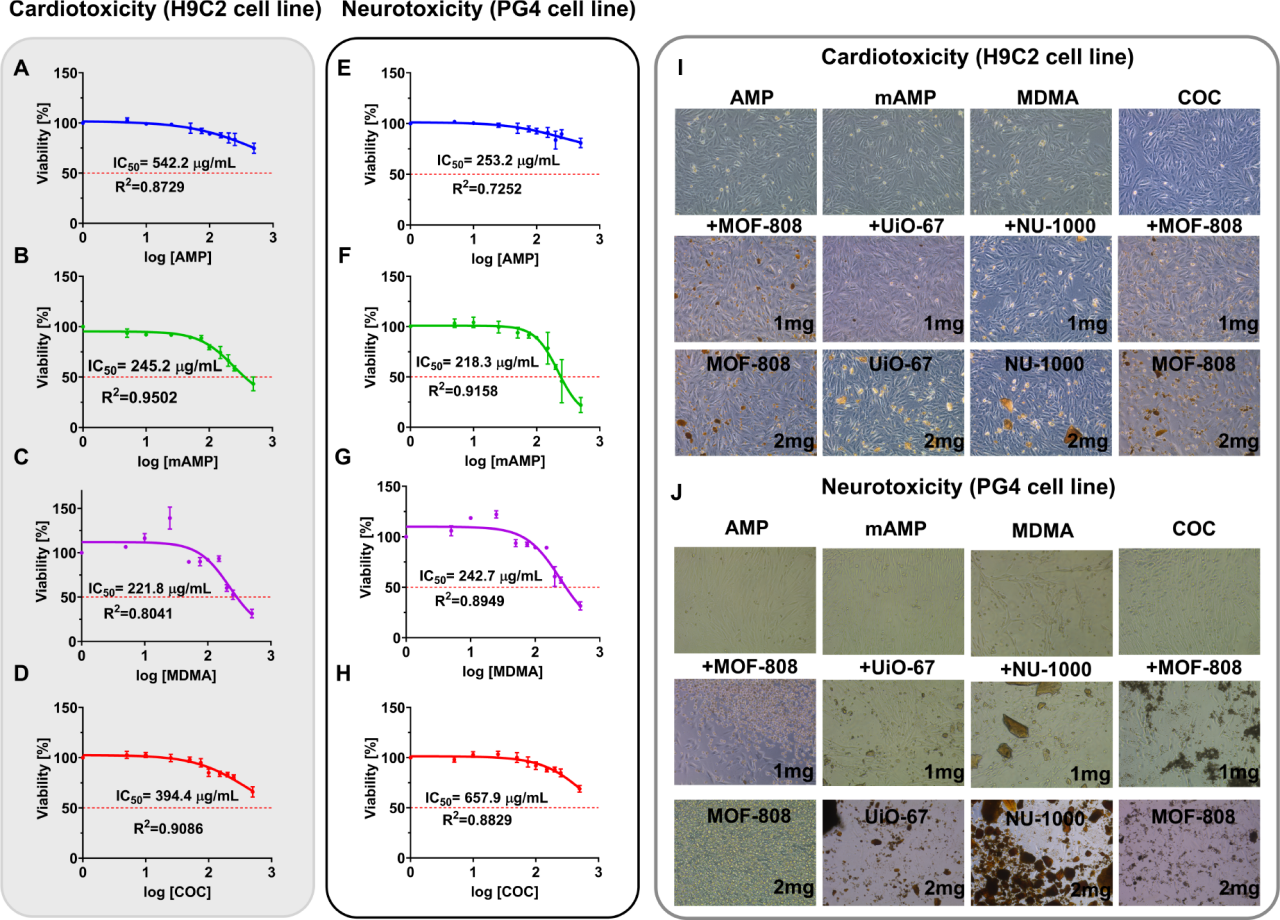
Effects of AMP, mAMP, MDMA, and COC on H9C2 (A–D) and PG-4 (E–H) cell viability. (I, J) The effect of AMP, mAMP, MDMA, and COC (250 μg/mL) alone (top row), with 1 mg/mL MOFs: MOF-808, UiO-67, and NU-1000 (middle row) and MOFs alone (2 mg/mL) (bottom row) on viability of cardiomyocytes of the H9C2 cell line (I) and astrocyte cell line PG-4 (J). The values of IC50 were calculated for each drug on each cell line. Data (A–-H) are presented as the mean ± SD, *n* = 3.

The *in vitro* experiments were performed to check the safety and effectiveness of the studied MOF in cellular models of neurotoxicity and cardiotoxicity, as the psychoactive substances seem to exert the most potent effects on those cells (organs). Psychoactive drugs like AMP, mAMP, MDMA, and COC, can have a significant impact on the cardiovascular system. These effects can vary depending on the drug, dosage, individual factors, and frequency of use. Our experiments revealed that the tested psychoactive substances do not exert direct cytotoxicity on neural or cardiac cells but rather activate receptors and neurotransmitters to evoke their effects within studied systems (Figure 4).

Images presented in Figure 4 prove the low toxicity of tested MOFs against PG-4 (I) and H9C2 (J) cell lines: bottom row. Then, we can observe, that the addition of MOFs to the cells intoxicated with tested psychoactive drugs did not significantly decrease the cells’ viability, but even promoted their growth (especially in the case of H9C2 cells).

The results obtained in *in vitro* experiments show positive effects of tested MOFs against psychoactive substance action in terms of safety and efficacy. However, to comprehensively understand the profound effect of MOF on the removal of psychoactive substances from organisms, the *in vivo* model should be implemented.

### In Vivo

In this study, zebrafish as an *in vivo* experimental model, appropriate for comparative studies on mammalian biology, was selected. *Danio rerio* is recommended by several international environments and health organizations (NIEHS, USA, and IES, Europe) as an excellent model to study environmental toxicity. Indeed, OECG recommendations on ecotoxicity testing include the method with the zebrafish embryo: OECG, test no. 236 Fish embryo acute toxicity testing. Moreover, the system is accepted by the National Institutes of Health (NIH, USA) as an alternative model to study the basis of human diseases.^57^

Zebrafish, used in our studies, are highly permeable to water and dissolved substances due to their thin, transparent skin and the presence of ion channels and transporters. Therefore, immersion in drug solutions allows for efficient absorption of substances through the skin, gut, and gills, mimicking potential routes of exposure. Further planned studies on rodents will involve specific routes of administration, such as intravenous, intragastric, or inhalation.

The experiments in the *Danio rerio* model gave interesting results concerning the effects of MOFs on the toxicity of psychoactive drugs. Psychoactive drugs such as AMP, mAMP, MDMA, and COC can exert their cardiotoxic and profound effects on the CNS, primarily by altering the levels and activities of various neurotransmitters (dopamine, norepinephrine, and serotonin) release. Their intake can lead to a range of neurological and psychological effects. *Danio rerio* can indeed be used as a model organism to study the neurotoxic and cardiovascular effects of psychoactive drugs. Zebrafish have gained popularity in scientific research due to their genetic similarities to humans, rapid development, transparency during the early stages of life, and ability to model various physiological and behavioral responses. Zebrafish share many similarities with humans in terms of neurotransmitter systems and pathways. While there are differences between species, zebrafish have conserved neurotransmitter systems that play key roles in various physiological and behavioral processes. These similarities in neurotransmitter systems provide a basis for studying the effects of psychoactive drugs on zebrafish behavior and physiology. By exposing zebrafish to drugs like AMP, mAMP, MDMA, and COC, changes in behaviors related to reward, locomotor activity, anxiety, and social interactions but also effects on the cardiovascular system can be observed. The locomotor activity test in zebrafish is a common behavioral assay used to assess the effects of drugs or other substances on the fish’s movement patterns. This test can provide insights into changes in motor function, coordination, and overall activity level, which can indirectly indicate neurological effects or neurotoxicity. It is important to recognize that while the fundamental neurotransmitter systems are conserved, there are also differences between zebrafish and humans in terms of brain structure, complexity, and specific receptor subtypes. Therefore, while zebrafish provide a valuable model for studying neurotransmitter-related effects, findings from zebrafish studies need to be extrapolated to humans with caution.

**Figure 5:**
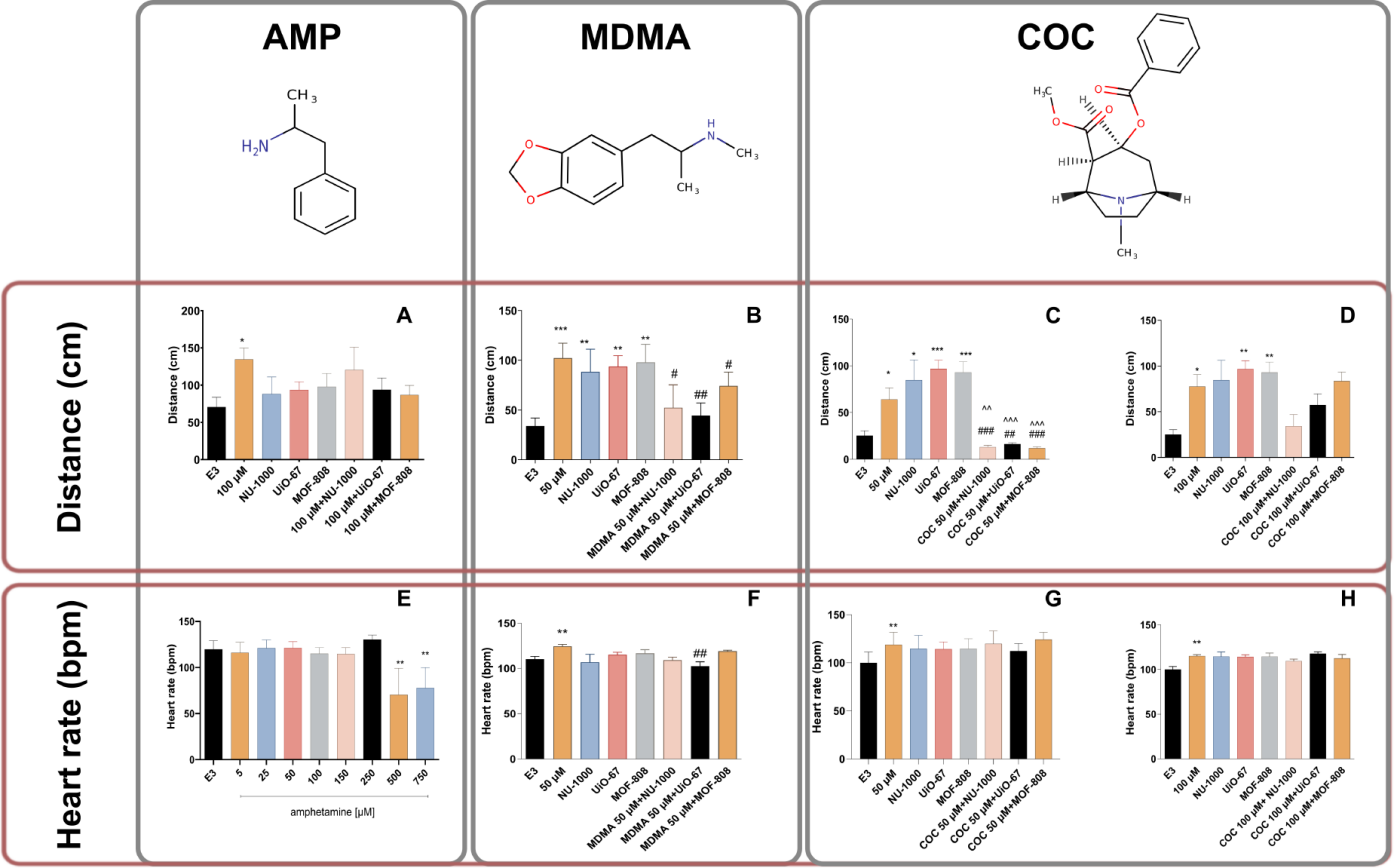
Effect of (A) AMP (100 μM) and metal–organic frameworks (MOFs: MOF-808, UiO-67, and NU-1000) (B) MDMA (50 μM) and MOFs, (C) COC (50 μM) and MOFs, and (D) COC (100 μM) and MOFs, on average distance (cm) moved by zebrafish larvae during the 10 min light phase. The effect of (E) AMP (5 μM, 25 μM, 50 μM, 100 μM, 150 μM, 250 μM, 500 μM, 750 μM), (F) MDMA (50 μM) and MOFs, (G) COC (50 μM) and MOFs, and (H) COC (100 μM) and MOFs, on heart rate was measured in 1 min (beat per minute). Data are presented as mean ± SEM, *n* = 12, *p < 0.05, **p < 0.01, ***p < 0.001 in comparison with E3 control group, #*p* < 0.05, ##*p* < 0.01, ###*p* < 0.001 in comparison with MDMA/COC control group, ^̂*p* < 0.01, ^̂̂*p* < 0.001 in comparison to the MOF-treated group; post hoc Tukey’s test.

Amphetamines induce the release of dopamine, norepinephrine, and serotonin from nerve terminals. As previously mentioned, this neurotransmitter surge activates the sympathetic nervous system, which leads to an increase in heart rate, blood pressure, and vasoconstriction. However, our preliminary results did not show an increase in heart rate when AMP was used in a wide range of doses (5–250 μM, Figure 5E, Table S9). The observed decrease in heart rate following incubation in higher concentrations (500 μM and 750 μM, Figure 5E) was a result of mortality induced by AMP. In the locomotor activity test, AMP at a concentration of 100 μM increased swim distance (*p* < 0.05). MOFs, as well as the combination of MOFs with AMP, did not significantly influence the locomotor activity of 5 dpf zebrafish (Figure 5A–D, Table S10).

MDMA is a synthetic drug that has both stimulant and mild hallucinogenic effects. Its effects on the cardiovascular system are somewhat different from those of COC and amphetamines. It can lead to an increase in heart rate, but this effect is generally not as pronounced as those with COC and amphetamines. MDMA primarily affects serotonin levels by causing the release of this neurotransmitter. While serotonin plays a role in mood regulation, its release in excessive amounts can lead to various effects, including increased heart rate and altered cardiovascular responses. MDMA also impacts the hypothalamus, the part of the brain responsible for regulating body temperature. It can interfere with the body’s thermoregulation, potentially leading to hyperthermia, which strains the cardiovascular system. Our studies confirmed that MDMA increased heart rate at the concentrations 50 μM — *p* < 0.001 and 100 μM — *p* < 0.001 (Figure S45A, Tables S11–S13). In the larvae incubated in the MOF solutions, we did not observe changes in the parameters observed. Only UiO-67 decreased MDMA-increased heartbeat (*p* < 0.01, Figure 5F).

In the locomotor activity test, MDMA at the concentration 25 μM — *p* < 0.05 and 50 μM — *p* < 0.05 increased swim distance. Also, MOFs influenced the locomotor activity of 5 dpf zebrafish in a statistically significant way (Figure 5B: NU-1000 — *p* < 0.01, UiO-67 — *p* < 0.01, MOF-808 — *p* < 0.01. Our results showed that coincubation of the larvae with MDMA at the concentration of 50 μM with studied MOFs decreased observed parameters: NU-1000 — *p* < 0.05, UiO-67 — *p* < 0.01, and MOF-808 — *p* < 0.01, when compared to MDMA treated group.

COC is a powerful stimulant that affects the CNS. It acts by blocking the reuptake of neurotransmitters like dopamine, leading to increased levels of these chemicals in the brain. COC also has potent vasoconstrictive properties. Our studies confirmed that COC increased heart rate at the concentrations: 50 μM — *p* < 0.001, 100 μM — *p* < 0.05, 150 μM — *p* < 0.05, 250 μM — *p* < 0.01, and 500 μM — *p* < 0.05 (Figure S46, Tables S14 and S15). No changes were observed in the monitored parameters in the larvae incubated in the MOF solutions. Also, MOFs did not influence COC-increased heartbeat (Figure 5G, H).

In the locomotor activity test, COC at the concentration of 25 μM — *p* < 0.05; 50 μM — *p* < 0.05; 100 μM — *p* < 0.01, 150 μM — *p* < 0.001, 250 μM — *p* < 0.05 increased swim distance (Figure S46B, Table S16). Additionally, MOFs influenced the locomotor activity of 5 dpf zebrafish in a statistically significant way (see Figure 5C — NU-1000: *p* < 0.05, UiO-67: *p* < 0.001, MOF-808: *p* < 0.001 and Figure 5D — UiO-67: *p* < 0.01, MOF-808: *p* < 0.01). Our results showed that coincubation of the larvae with COC at the concentration 50 μM with studied MOFs decreased observed parameters: NU-1000 — *p* < 0.001, UiO-67 — *p* < 0.01, MOF-808 — *p* < 0.001, when compared to COC treated group, and: NU-1000 — *p* < 0.01, UiO-67 — *p* < 0.001, and MOF-808 — *p* < 0.001, in comparison to groups treated with MOFs (Tables S9–S11).

## Conclusions

In summary, in this work, we present the concept of the application of zirconium-based metal–organic frameworks for the removal of illicit drugs that can be used on-site by nonspecialists. The concept of safe and efficient metal–organic frameworks was confirmed in this study for popular illicit drugs of abuse, including amphetamine, methamphetamine, MDMA, and cocaine. The selected model zirconium-based metal–organic frameworks revealed high adsorption efficiency reaching up to 90% removal in the case of NU-1000 from water and almost 100% from SNF solution. Additionally, the high adsorption efficiency of NU-1000 was confirmed in a multidrug adsorption experiment, where overall MDMA and COC removal was equal to 90% and 100%, respectively.

The illicit drug sorption mechanisms were determined by DFT modeling, showing in the vast majority of cases the π–π stacking, confirmed by both the geometrical relationship of the interacting aromatic rings and the electronic structure-derived quantities (atomic charges, bond orders). The DFT-based vibrational analysis and the Raman intensities modeling rationalized the Raman spectra.

The DFT modeling, via harmonic vibrational analysis, allowed for the unequivocal assignment of the experimental Raman bands and thus for the determination of the diagnostic bands. The elucidation of the sorption mechanism was possible via the concepts bridging the gap between the accurate and formal world of quantum chemistry, and the chemical intuition–namely the atomic charges and the bond orders–inaccessible otherwise, e.g., experimentally.^58^

The dominant mode of adsorption, the π–π stacking, was determined for all but for two structures of COC@UiO-67. For all studied MOFs, however, the sorption of the largest molecule, cocaine, was the strongest. The calculated bond orders held the same tendency.

Additionally, the efficient adsorption of considered drugs of abuse was confirmed by Raman differential spectroscopy, corroborated by DFT vibrational analysis. It is worth mentioning that the developed methodology allowed us to confirm the presence of illicit drugs adsorbed on MOFs even at concentrations as low as 5 mg/g. The use of RDS corroborated with DFT methods facilitates the direct detection of low concentrations of drugs in the MOF matrix and may be an alternative to indirect methods utilizing mineralization techniques such as dilution chromatography.

The *in vitro* cardiotoxicity and neurotoxicity experiments have confirmed low cytotoxicity of the considered Zr-MOFs, showing their prospective application for living organisms. Additionally, the *in vivo* experiments have demonstrated that COC and MDMA seem to have notable impacts on the heart function and heart rate of zebrafish, indicating potential cardiotoxic effects. On the other hand, AMP did not demonstrate such effects on the heart. All of the psychoactive substances tested increased the locomotor activity of zebrafish, which suggests that they have stimulant properties affecting movement. However, the presence of MOFs seemed to counteract the locomotor effects induced by COC and MDMA, which might imply a modulating or inhibitory role of MOFs in the context of the CNS rather than the cardiovascular system.

The concept of the application of Zr-MOFs, proposed in this study, opens new strategies in the development of novel drug removal systems that may be used on-site by nonspecialists in emergencies. Indeed, the real application of Zr-MOF detoxification systems requires both preclinical and clinical trials. The former is currently under investigation in our research group, and the results are promising and hopefully will serve as preliminary data for clinical trials. The prospective application of detox systems based on MOFs would strongly support the fight against abused drugs worldwide.

## Data Availability

The data that support the findings of this study are openly available in Jodłowski, Przemysław (2024), Crystal Clear: Metal–Organic Frameworks Pioneering the Path to Future Drug Detox. *Mendeley Data*, V1, DOI: 10.17632/t8g6p9hyt2.1.

## Supporting Information Available

Materials; synthesis; characterization; additional experimental results (PDF)
