# Supplementary material for: Crystal Clear: Metal–Organic Frameworks Pioneering the Path to Future Drug Detox
Source: ACS Appl Mater Interfaces. 2024 May 30;16(23):29657–71. doi: 10.1021/acsami.4c02450 (PMC11181303; doi:10.1021/acsami.4c02450)
Supplement: Supplementary file 1 — am4c02450_si_001.pdf [file am4c02450_si_001.pdf]

Supporting Information

Crystal Clear: Metal-Organic Frameworks Pioneering the Path to Future Drug Detox

Przemysław J. Jodłowski,[*,†] Klaudia Dymek,[†,‡] Grzegorz Kurowski,[†] Kornelia Hyjek,[†] Anna Boguszewska-Czubara,[¶] Barbara Budzyńska,[§] Weronika Mrozek,[§] Norbert Skoczylas,[‖] Łukasz Kuterasiński,[⊥] Witold Piskorz,[#] Marek Białoruski,[#] Roman J. Jędrzejczyk,[@] Piotr Jeleń,[△] and Maciej Sitarz[△]

†Faculty of Chemical Engineering and Technology, Cracow University of Technology, Warszawska 24, 31-155 Kraków, Poland

‡Lukasiewicz Research Network – Krakow Institute of Technology, Zakopiańska 73, 30-418 Kraków, Poland

¶Department of Medical Chemistry, Medical University of Lublin, Chodzki 4A, 20-093 Lublin, Poland

§Independent Laboratory of Behavioral Studies, Medical University of Lublin, Chodzki 4A, 20-093 Lublin, Poland

‖Faculty of Geology, Geophysics and Environmental Protection, AGH University of Krakow, Mickiewicza 30, 30-059 Kraków, Poland

⊥Jerzy Haber Institute of Catalysis and Surface Chemistry, Polish Academy of Sciences, Niezapominajek 8, 30-239 Kraków, Poland

#Faculty of Chemistry, Jagiellonian University in Kraków, Gronostajowa 2, 30-387 Kraków, Poland

@Małopolska Centre of Biotechnology, Jagiellonian University in Kraków, Gronostajowa 7A, 30-387 Kraków, Poland

△Faculty of Materials Science and Ceramics, AGH University of Krakow, Mickiewicza 30, 30-059 Kraków, Poland

Corresponding author: przemyslaw.jodlowski@pk.edu.pl

Experimental section

Materials

Zirconium oxide dichloride octahydrate ($ZrOCl_2 \times 8H_2O$, Alfa Aesar, 98%), Zirconium chloride ($ZrCl_4$, Merck, 98%), 1,3,5-benzenetricarboxylic acid (H_3BTC, TCI, 98%), 1,1’-biphenyl-4,4’-dicarboxylic acid (BPDC, Angene, 95%), benzoic acid (Acros, 99%), formic acid (Chempur, 85%), hydrochloric acid (Eurochem, 35-38%), *N*,*N*-dimethylformamide (Chempur, 98%), H_4TBAPy, acetone (Chempur, 98%), amphetamine (**AMP**, LGC Standards), methamphetamine (**mAMP**, LGC Standards), 3,4-methylenedioxymethamphetamine (**MDMA**, THC Pharm), cocaine (**COC**, LGC Standards).

Synthesis

NU-1000

0.338 g of $ZrOCl_2 \times 8H_2O$ and 10.8 g of benzoic acid were dissolved in 32 mL of DMF in an ultrasonic bath (20 min). The solution was placed in a glass bottle and heated for 1 hour at 80 °C. After cooling, 0.16 g of H_4TBAPy was added to the solution and placed in an ultrasonic bath for 10 min. The mixture was placed in the oven for 15.5 h at 100 °C and, after cooling down, centrifuged and washed three times with 20 mL of DMF. The crystals were dispersed in 52 mL of DMF and 2 mL of 8 M HCl solution and placed in an oven for 12 hours at 100 °C. After cooling, the material was placed in 20 mL DMF for 2 hours. After centrifuging the crystals, the operation was repeated twice more. The crystals were then flooded with acetone three times for 12 hours. The prepared material was left for 1 hour at 80 °C in a vacuum.

UiO-67

0.54 g of $ZrCl_4$ was dissolved in 9 mL of DMF and 250 µL of water by stirring at 80 °C for 10 min. 0.85 g of benzoic acid was added to the solution and placed in an ultrasonic bath for 10 min. Next, 0.56 g of 1,1'-biphenyl-4,4'-dicarboxylic acid was added to the solution and poured into a glass bottle, which was placed in an oven at 130 °C for 16 hours. The cooled crystals were centrifuged and washed 5 times with 10 mL of DMF and 3 times with 10 mL of acetone. The obtained material was left to dry overnight at 80 °C.

MOF-808

0.22 g of $ZrOCl_2 \times 8H_2O$ and 0.15 g of 1,3,5-benzenetricarboxylic acid were dissolved in 12 mL of DMF. 13 mL of formic acid was added to the solution and stirred for 20 min in a glass bottle. Then the bottle with the solution was placed in an oven at 110 °C for 48 hours. After cooling, the crystals were filtered and heated at 100 °C in 30 cm^3 DMF for 10 hours. The cooled crystals were centrifuged and washed 3 times with 10 cm^3 of DMF and 2 times with 10 cm^3 of acetone. The obtained material was left to dry overnight at 80 °C.

Characterization

Powder X-ray diffractometry (PXRD)

The crystallinity of the materials was tested using the powder X-ray diffractometry (PXRD) on X'Per Pro MPD (PANalytical) diffractometer with a copper lamp (CuKα radiation, λ = 1.5406 Å). The theoretical PXRD formula for "ideal" UiO-66 was calculated based on the .cif file and Mercury 4.2.0 software.

Low temperature nitrogen adsorption

Zr-MOF was subjected to low-temperature gas adsorption analysis using an ASAP 2020 analyser. The apparatus operated in the pressure range of 0-0.1 MPa at 77 K. Prior to measurement, the samples were degassed for 12 hours under vacuum at 423 K, and nitrogen was used as adsorbate. The surface area was calculated using the multilayer BET surface filling (S_{BET}) and single layer Langmuir (S_{Lang}) model. The total pore volume (V_{micro}) and pore distribution were determined according to the non-local density functional theory (NLDFT) method dedicated to cylindrical pores, respectively. The average pore diameter was determined based on the BET model.

μRaman spectroscopy

Raman measurements were performed using WITec Alpha 300M+ spectrometer equipped with 50× objective, 785 nm diode laser, and 300 grating. Each sample was measured 10 times, 60 s each, and then averaged. Laser power was each time adjusted to prevent sample degradation. All of the obtained spectra were subjected to baseline treatment and normalization.

SEM

The scanning electron microscopy images were collected by a Nova Nano SEM 300 FEI microscope. Prior to analysis, the MOF samples were deposited on graphite holders from ethanolic solutions.

DFT

Computational methods

The periodic density functional theory (DFT) calculations were performed with the use of the VASP code [1–4]. The PBE [5,6] exchange-correlation functional was used and, to account for the London dispersion part of the van der Waals interactions, the semi-empirical method parametrised by Grimme was applied [7,8]. The plane wave basis set energy cut-off was chosen as 400 eV. As a convergence accelerator, the Gaussian smearing of the Fermi-Dirac distribution was used with width of 0.01 eV.

Due to the large size of the unit cells (see Table S1), the Γ-point only *k*-space sampling was found to be sufficient allowing for the separation of the *k*-points in the Irreducible Brillouin Zone.

In order to compare the adsorption energies among different MOFs, aware of the incomplete basis set, in case of each MOF structure the adsorption energy was calculated with use of the total energy of a single drug molecule in the unit cell of the given MOF. It is noteworthy that the differences between the total energies of the same molecule in different unit cells were as high as *ca*. 0.4 eV.

The initial structures in the DFT modelling were obtained by the Monte-Carlo simulations at the classical force field level of theory (Universal Force Field) [9]. The atomic charges and bond orders were calculated with use of the Henkelman group's Bader analysis toolkit [10–13] and the Density Derived Electrostatic and Chemical (DDEC6) population analysis and bond order package Chargemol [14–18].

Vibrational analysis and Raman spectra

The harmonic vibrational analysis was performed by means of the finite displacement method to obtain the wavenumbers and then the Raman intensities were calculated from the static ion-clamped dielectric matrix using density functional perturbation theory (DFPT)[19–21]. The full Hessian was obtained in the case of all adsorbate@MOF systems. For the systems with more

than one stable localisation of the adsorbate, the Hessian was calculated for the most stable conformer only. To reduce the computational cost of the Raman intensities modelling, only the modes corresponding to the experimentally important (diagnostic) bands were selected in the intensities' simulations. The selected modes were also verified for the involvement of the linker atoms.

Computational models

The models were built based on the literature .CIF files and the geometries were optimised.

Table S1. Unit cell parameters of the computational models.

MOF	Stoichiometry	Unit cell vectors length / Å			Unit cell vectors angles / °		
		h	*k*	*l*	α	β	γ
NU-1000 [22]	$C_{264}H_{180}Zr_{18}O_{96}$	39.970	40.000	16.580	90.0	90.0	120.0
UiO-67 [23]	$C_{84}H_{52}Zr_{6}O_{32}$	19.012	19.012	19.012	60.0	60.0	60.0
MOF-808 [24]	$C_{96}H_{64}Zr_{24}O_{128}$	25.001	25.000	24.997	59.70	59.72	59.72

Sorption studies

Sorption kinetic studies of psychoactive compounds (AMP, mAMP, MDMA, and COC) were carried out to check the sorption capacity of the materials: MOF-808, UiO-67, and NU-1000. The studies were conducted according to the procedures described in our previous publications with some modifications[25,26]. Sorption of psychoactive substances was carried out under thermostatic conditions (25 °C). 10 mg of the activated material was placed in 2 mL of a 500 µM psychoactive substance solution. In order to ensure repeatability of results, each sorption test was repeated three times. To determine the kinetics and the amount of adsorbed substances as a function of time, 0.1 mL of the solution was collected at 0 min, 5 min, 15 min, 30 min, 60 min, 2 h, 6 h, and 24 h of the test. The sample filtered through a 33 mm, 0.22 µm, PTFE syringe filter was analysed by MS-HPLC for AMP and mAMP, and by HLPC for MDMA and COC.

The equilibrium amount of adsorbed psychoactive substances and the pseudo-first and pseudo-second-order kinetic models were calculated using formulas S1-S3. The rate constant and the equilibrium amount of substance as a function of time were determined from equations S4 and S5.

$$q_e = (C_0 - C_e) \cdot \frac{V}{W} \; (S1)$$

$$\log(q_e - q_t) = \log q_e - \frac{k_1 t}{2.303} \; (S2)$$

$$\frac{t}{q_t} = \frac{1}{k_2 q_e^2} + \frac{t}{q_e} \; (S3)$$

$$k_2 = \frac{(slope)^2}{intercept} \; (S4)$$

$$q_e = \frac{1}{slope} \; (S5)$$

Where:

C_0 - initial concentration [mg/L], C_e - equilibrium concentration [mg/L], V - volume of the tested solution [L], W - mass of adsorbent [g], q_t - amount of substance adsorbed after time t, q_e - equilibrium amount of adsorbed substance, k_1, k_2 - constant for pseudo-first and pseudo-second order of adsorption.

Results

Characterisation

Table S2. Geometrical descriptors of selected drugs. Corresponding projections depicted in Figure 1.

Descriptor	Minimal projection area / Å^2	Maximal projection area / Å^2	Minimal projection radius / Å	Maximal projection radius / Å	Length perpendicular to the max area / Å^2	Length perpendicular to the min area / Å^2	van der Waals volume / Å^3
**AMP**							
	28.16	49.22	3.58	5.22	5.72	10.36	144.89
**mAMP**							
	31.17	55.18	3.87	5.82	6.41	11.64	162.41
**MDMA**							
	34.26	64.84	4.24	5.99	6.12	11.84	185.76

COC							
	51.87	84.95	5.38	7.31	8.59	14.47	283.89

PXRD and N_2 adsorption isotherms

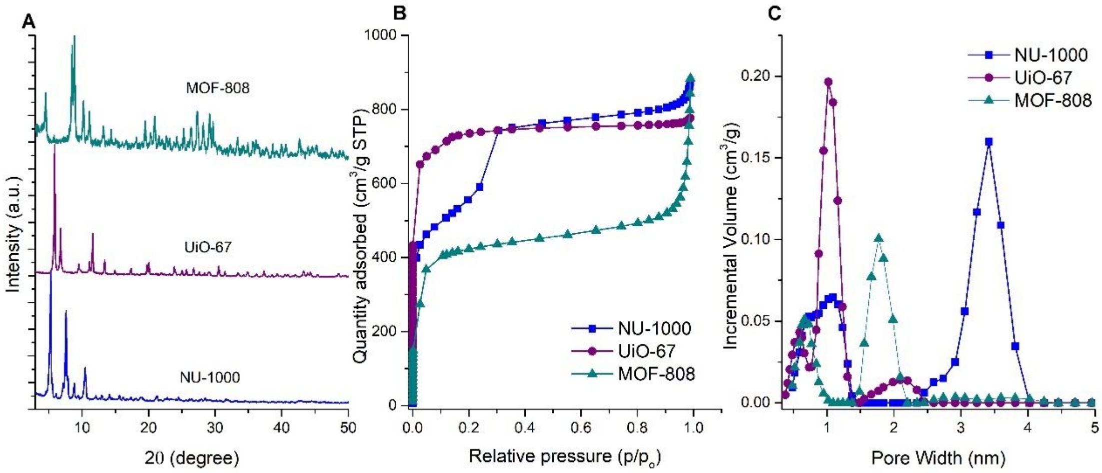

Figure S1. (A) PXRD; **(B)** N_2 adsorption isotherms; **(C)** pore size distribution (PSD) for parent MOFs samples

SEM analysis

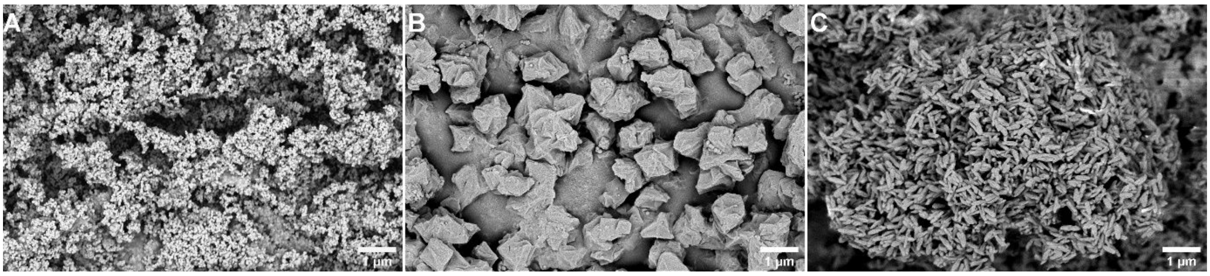

Figure S2. SEM micrographs of parent MOF samples; **(A)** MOF-808, **(B)** UiO-67, and **(C)** NU-1000

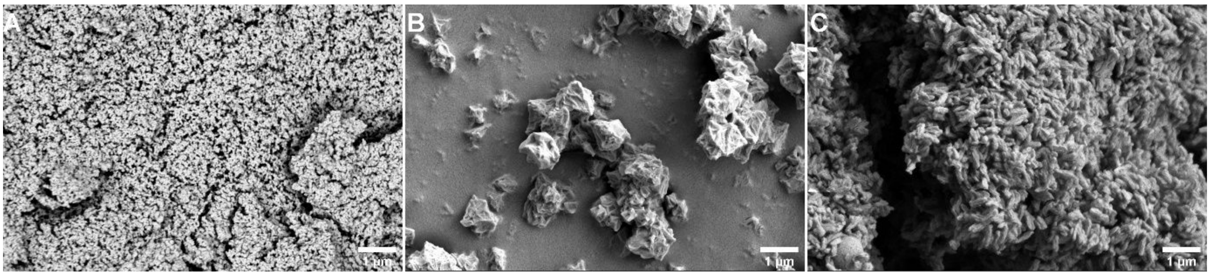

Figure S3. SEM micrographs of prepared samples after the adsorption of **(A)** AMP@MOF-808, **(B)** AMP@UiO-67, and **(C)** AMP@NU-1000

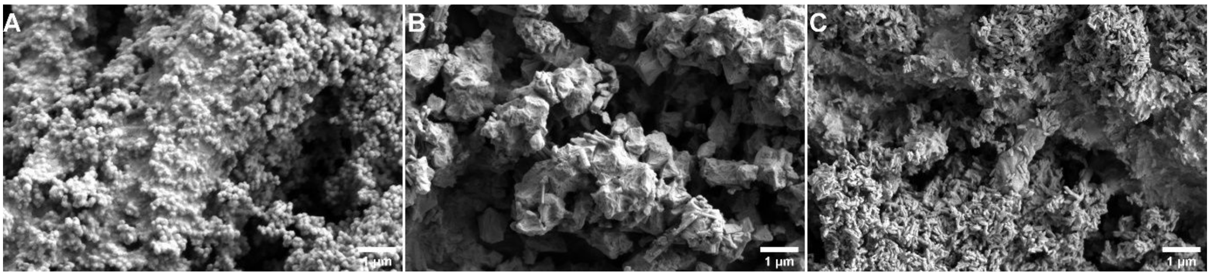

Figure S4. SEM micrographs of prepared samples after the adsorption of **(A)** mAMP@MOF-808, **(B)** mAMP@UiO-67, and **(C)** mAMP@NU-1000

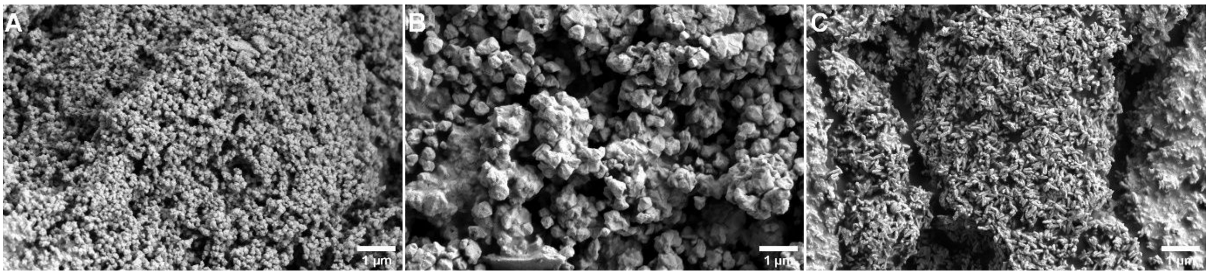

Figure S5. SEM micrographs of prepared samples after the adsorption of **(A)** COC@MOF-808, **(B)** COC@UiO-67, and **(C)** COC@NU-1000

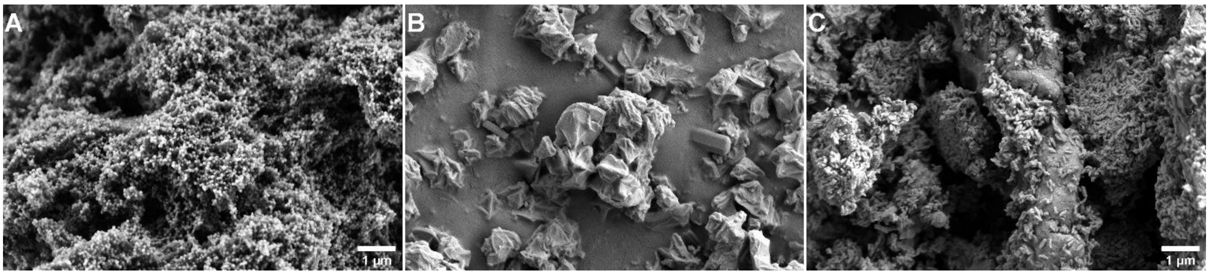

Figure S6. SEM micrographs of prepared samples after the adsorption of **(A)** MDMA@MOF-808, **(B)** MDMA@UiO-67, and **(C)** MDMA@NU-1000

μRaman analyses

Table S3. Calculated and experimental μRaman spectra and corresponding band assignment for AMP, mAMP, MDMA, and COC adsorbed in MOF-808. RS = Raman shift. Intensity: m = medium; (v)s = (very) strong; w = weak; br = broad.

**AMP** [2728]		**mAMP** [2728]		**MDMA** [29]		**COC** [2830]	
RS / cm^{-1} (intensity)	Assignment	RS / cm^{-1} (intensity)	Assignment	RS / cm^{-1} (intensity)	Assignment	RS / cm^{-1} (intensity)	Assignment
256 (s)	CN bending	622 (s)	Chain CH, Ring out-of-plane def.	341 (s)	C-N-C	854 (w)	C-C stretch. Tropane ring
622 (s)	Chain CH, Ring out-of-plane def.	838 (vs)	Ring out-of-plane def.	535 (s)	C-O-C	870 (m)	
829 (s)	Ring out-of-plane def.	1003 (vs)	Ring and chain CC stretching	717 (s)	Ring and NH vibr.	897 (w)	
1002 (vs)	Ring and chain CC stretching	1021 (m)	Ring and chain CC stretching	776 (s)	C-O-C	1002 (s)	Symm. stretching, arom. ring breath.
1032 (s)	Ring and chain CC stretching	1032 (m)	Ring and chain CC stretching	814 (vs)	Asymm. and rocking C-C	1027 (m)	Asymm. stretching, arom. ring breath.
1158 (m)	CH bending	1182 (w)	CH bending	1371 (s)	Asymm. and rocking C-C	1270 (w)	C-N stretching
1182 (m)	CH bending	1210 (s)	CH_2, CH_3 def.	1443 (s)	Asymm. and rocking C-C	1597 (m)	Arom. Ring C-C stretching
1210 (s)	CH_2, CH_3 def.	1455 (br)	Ring in-plane CH bending	1610 (m)	Ring and NH vibr.	1716 (m)	C=O stretching
1369 (w)	CH_3 def.	1585 (m)	NH def. vibr.	1632 (m)	Ring and NH vibr.		
1450 (w)	Ring in-plane CH bending	1605 (m)	NH def. vibr.				
1583 (w)	NH def. vibr.						
1607 (w)	NH def. vibr.						

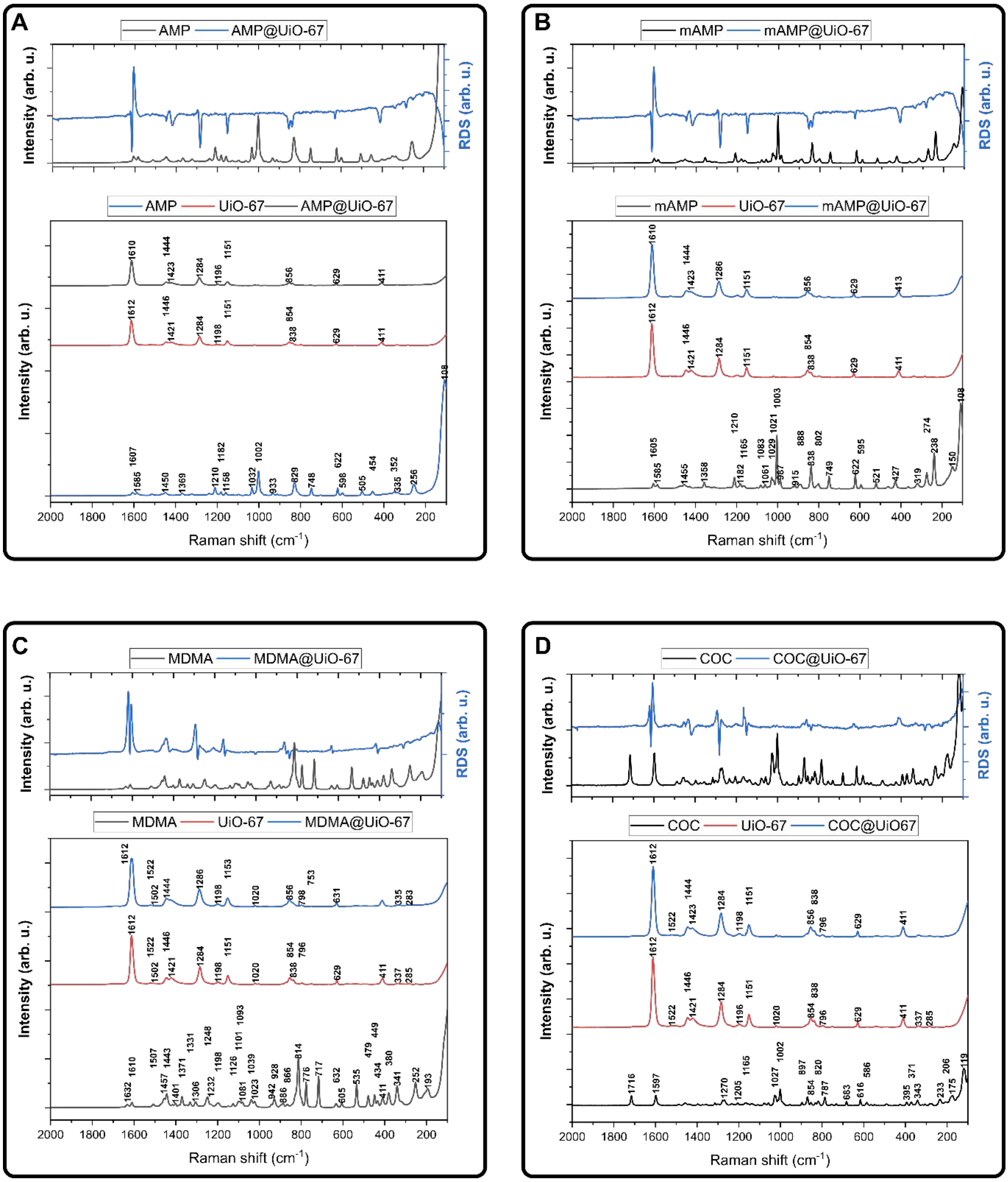

Figure S7. µRaman difference spectroscopy results, RDS, (upper plots) and µRaman analyses (lower plots) of UiO-67 after adsorption of **(A)** AMP, **(B)** mAMP, **(C)** MDMA, and **(D)** COC.

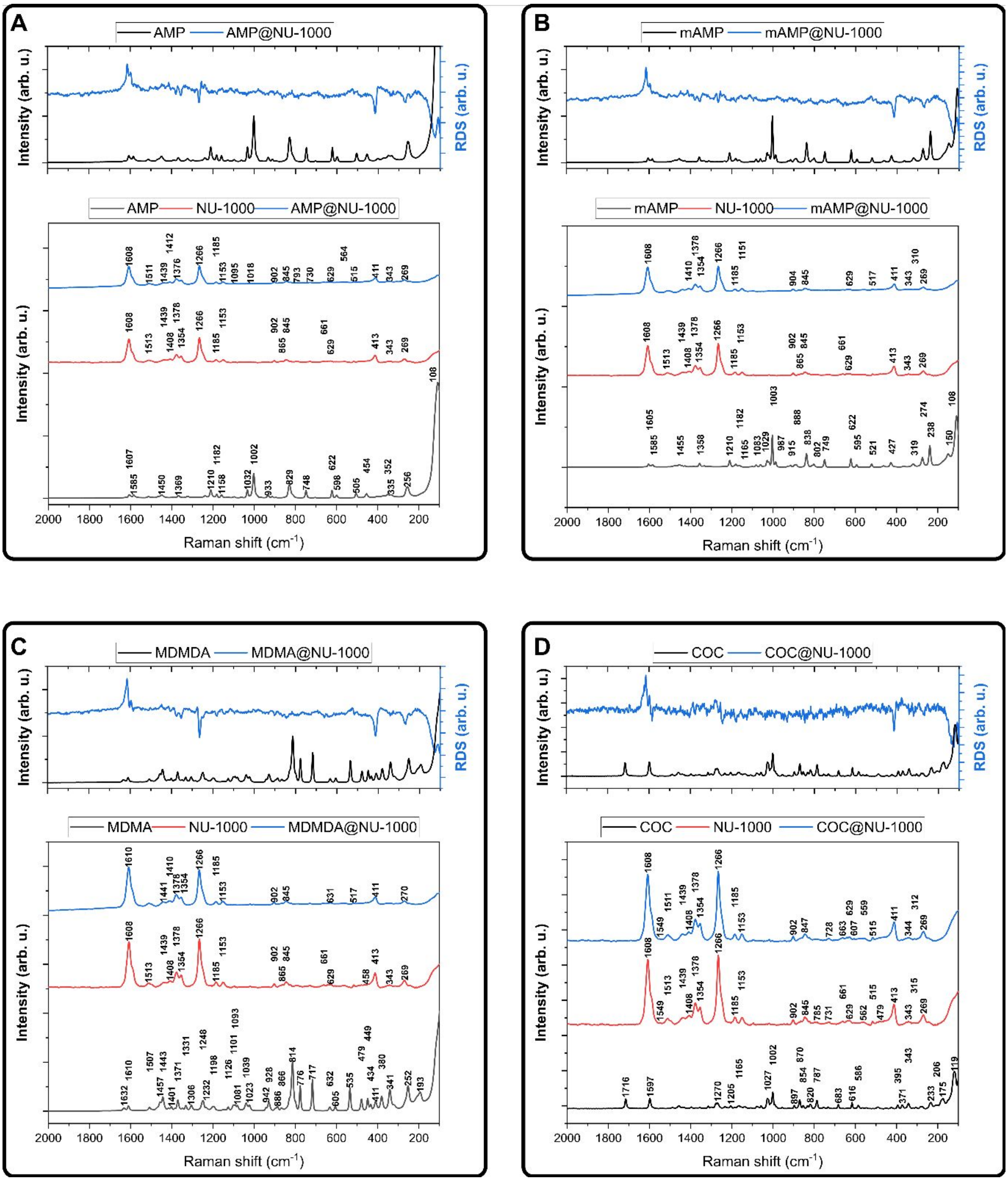

Figure S8. μRaman difference spectroscopy results, RDS, (upper plots) and μRaman analyses (lower plots) of NU-1000 after adsorption of **(A)** AMP, **(B)** mAMP, **(C)** MDMA, and **(D)** COC.

Adsorption of AMP, methamphetamine, MDMA, and COC in Zr-MOFs

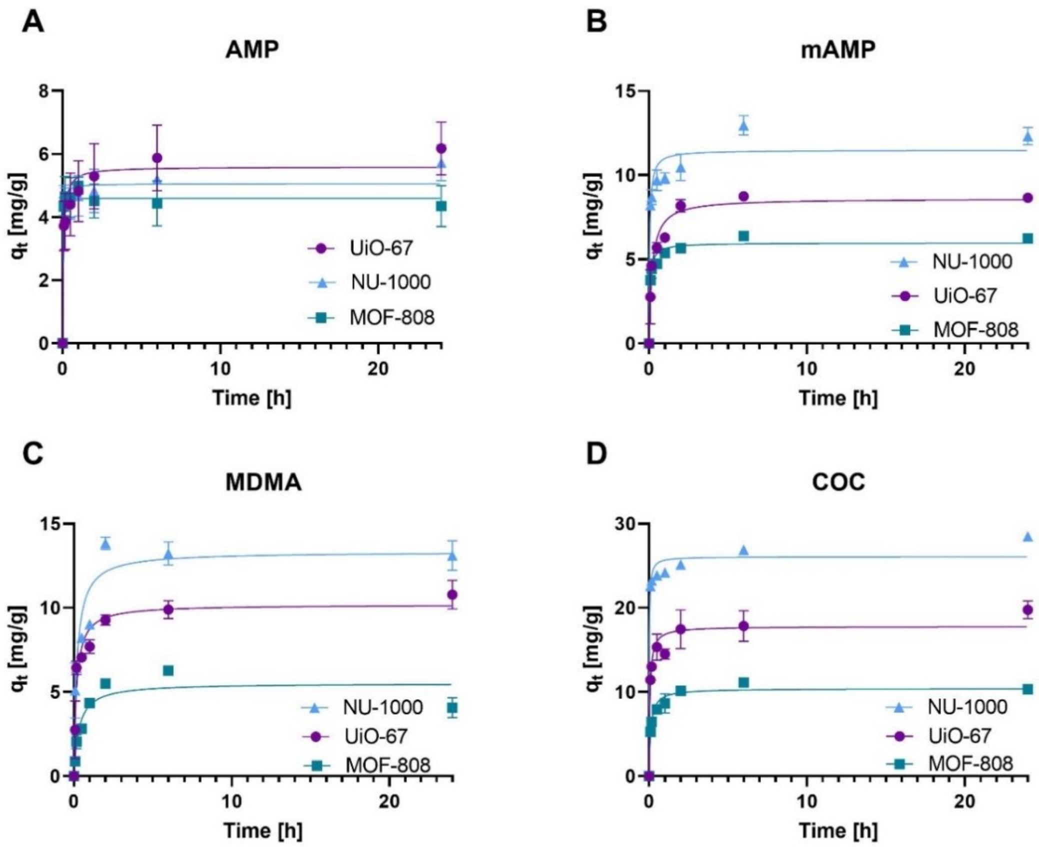

Figure S9. Removal of **(A)** AMP, **(B)** mAMP, **(C)** MDMA, and **(D)** COC over Zr-MOFs

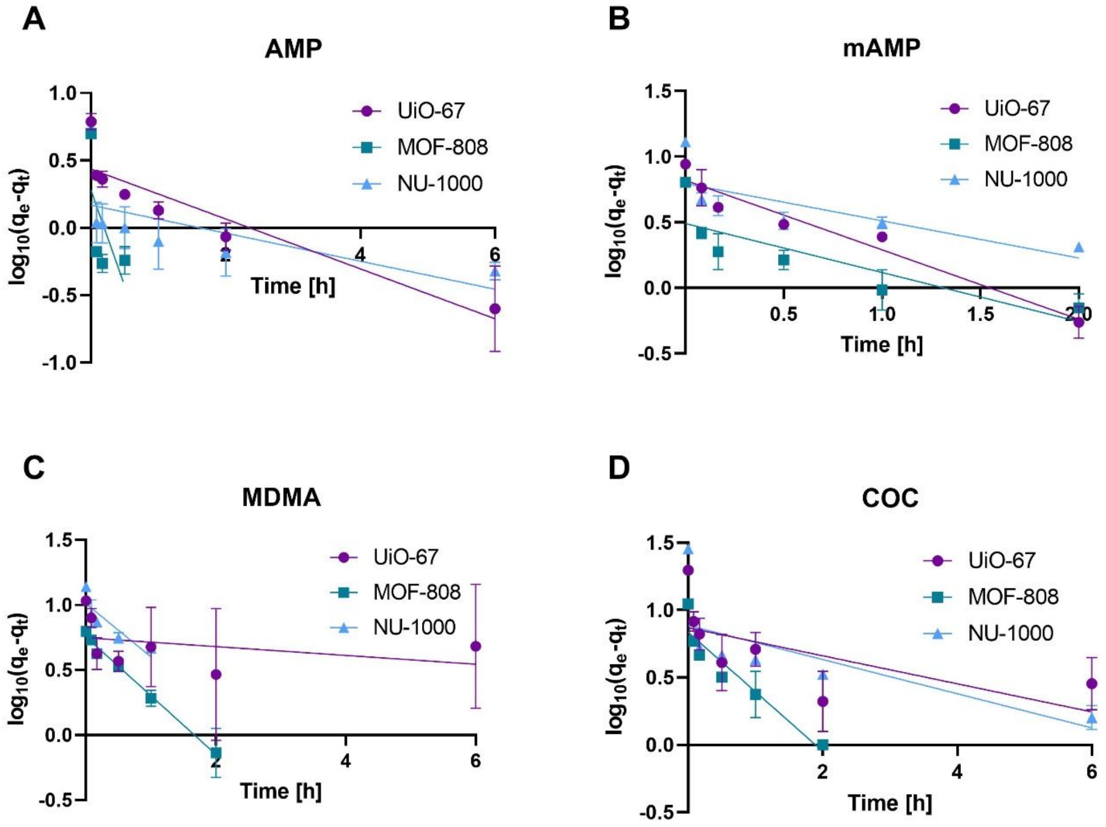

Figure S10. Pseudo-first order kinetic model of adsorption of **(A)** AMP, **(B)** mAMP, **(C)** MDMA, and **(D)** COC over Zr-MOFs;

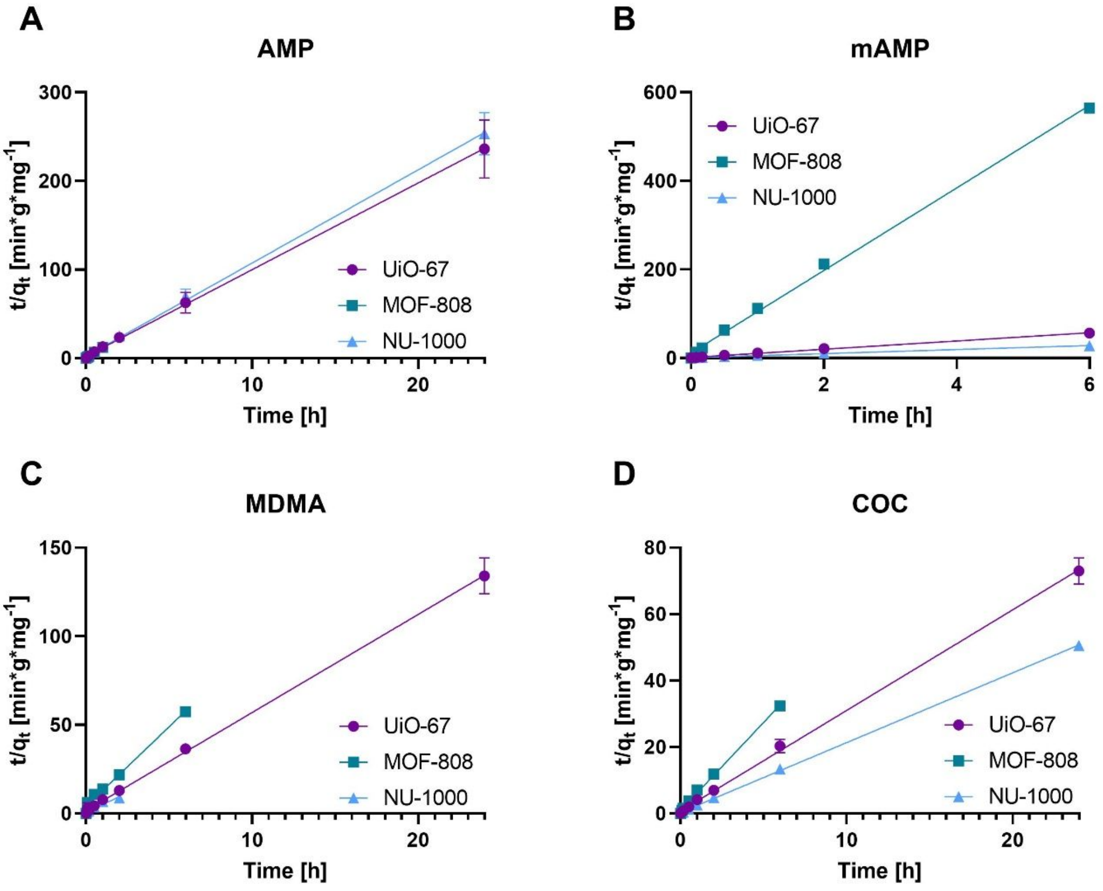

Figure S11. Pseudo-second order kinetic model of adsorption of **(A)** AMP, **(B)** mAMP, **(C)** MDMA, and **(D)** COC over Zr-MOFs;

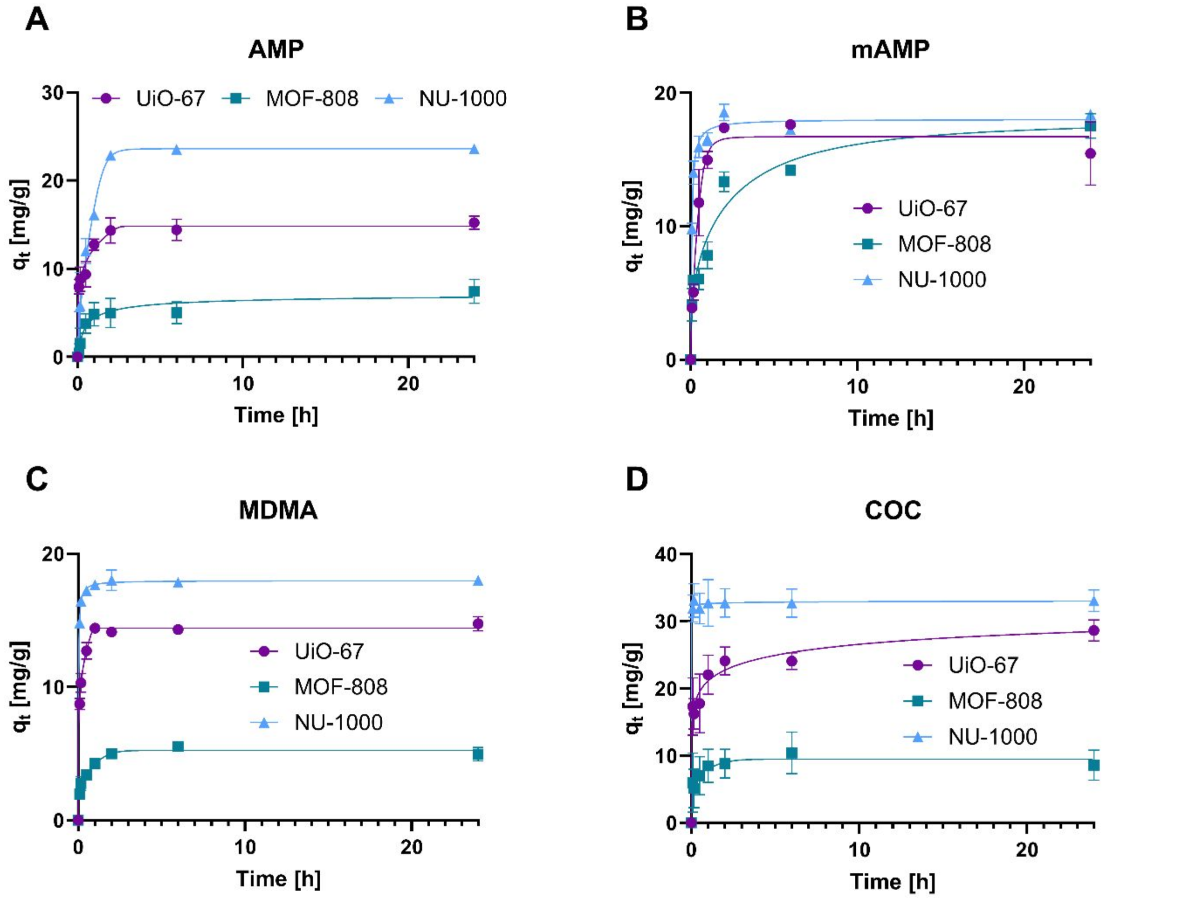

Figure S12. Removal of **(A)** AMP, **(B)** mAMP, **(C)** MDMA, and **(D)** COC over Zr-MOFs from SNF solution;

Table S4. Kinetic parameters of pseudo-first order and pseudo-second order models for AMP, mAMP, MDMA, and COC over Zr-MOFs in water environment

**AMP**						
	Pseudo-first order			Pseudo-second order		
**MOF**	k_1 / (1/min)	q_e / (mg/g)	R^2	k_2 / (g/(mg · min))	q_e / (mg/g)	R^2
**NU-1000**	0.010	1.85	0.38	0.041	5.15	0.99
**UiO-67**	0.012	3.23	0.72	0.029	5.84	0.99
**MOF-808**	0.049	1.74	0.36	0.264	4.98	0.99
**mAMP**						
	Pseudo-first order			Pseudo-second order		
**MOF**	k_1 / (1/min)	q_e / (mg/g)	R^2	k_2 / (g/(mg · min))	q_e / (mg/g)	R^2

**NU-1000**	0.009	6.03	0.5	0.0082	12.99	0.99
**UiO-67**	0.020	6.64	0.95	0.0084	9.01	0.99
**MOF-808**	0.014	3.13	0.72	0.0222	6.44	0.99
**MDMA**						
**MOF**	Pseudo-first order			Pseudo-second order		
	k_1 / (1/min)	q_e / (mg/g)	R^2	k_2 / (g/(mg · min))	q_e / (mg/g)	R^2
**NU-1000**	0.027	11.31	0.95	0.0057	13.91	0.93
**UiO-67**	0.003	5.18	0.39	0.0062	10.81	0.99
**MOF-808**	0.017	5.71	0.99	0.0053	6.79	0.99
**COC**						
**MOF**	Pseudo-first order			Pseudo-second order		
	k_1 / (1/min)	q_e / (mg/g)	R^2	k_2 / (g/(mg · min))	q_e / (mg/g)	R^2
**NU-1000**	0.0051	7.76	0.55	0.0045	28.57	0.99
**UiO-67**	0.0037	7.59	0.41	0.0039	19.61	0.99
**MOF-808**	0.0170	6.92	0.89	0.0093	11.36	0.99

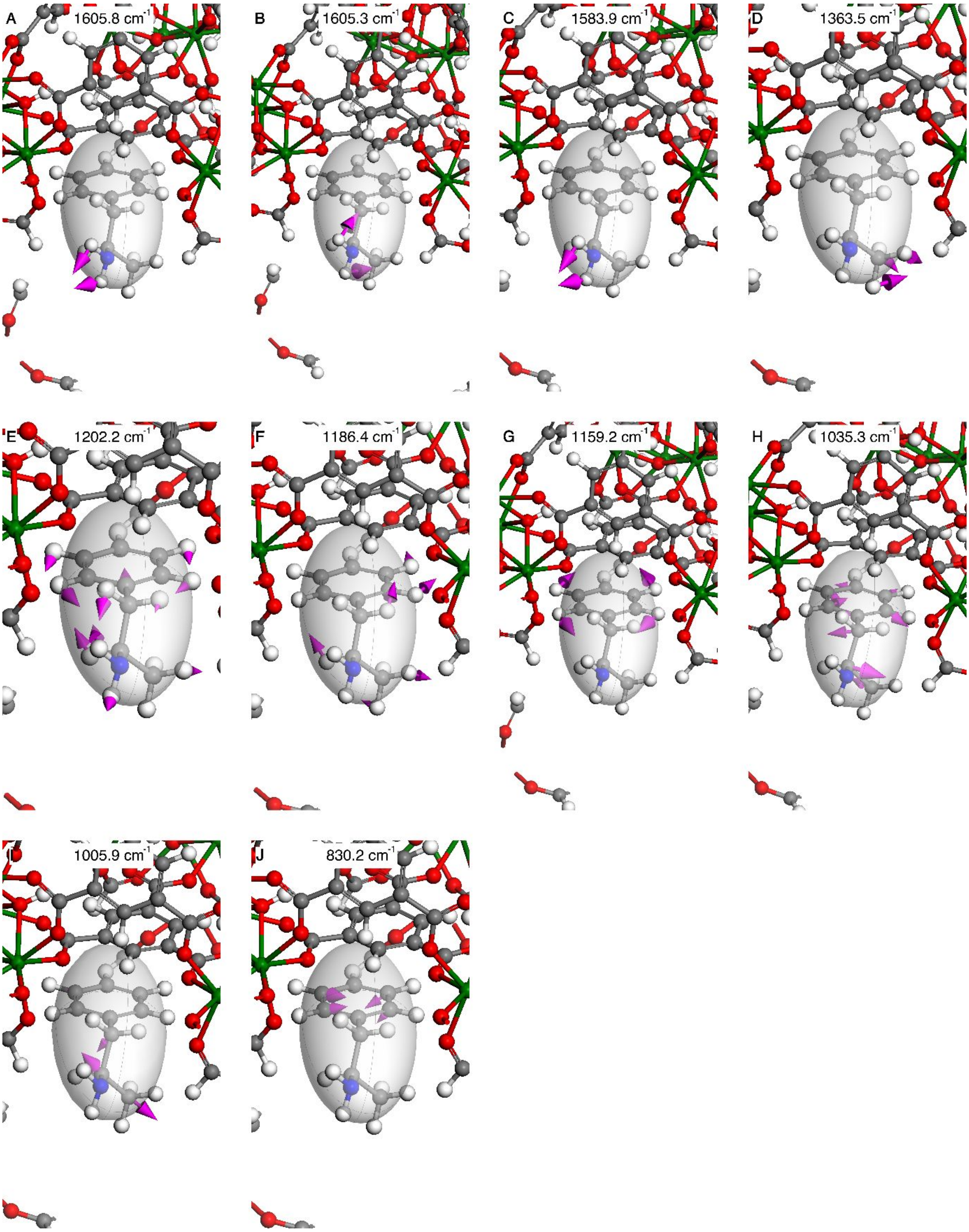

Figure S13. DFT optimised structures of AMP adsorbed in MOF-808 with vibrational eigenvectors depicted by arrows with lengths scaled by the Raman intensities; **(A)** deformation/scissoring NH_2**, (B)** deformation NH_2, **(C)** deformation NH_2, **(D)** deformation CH_3, **(E)** deformation CH_3 and CH_2, **(F)** bending CH, **(G)** bending CH, **(H)** aromatic ring and

aliphatic chain CC stretch, **(I)** aromatic ring and aliphatic chain CC stretch, **(J)** aromatic ring out-of-plane deformation

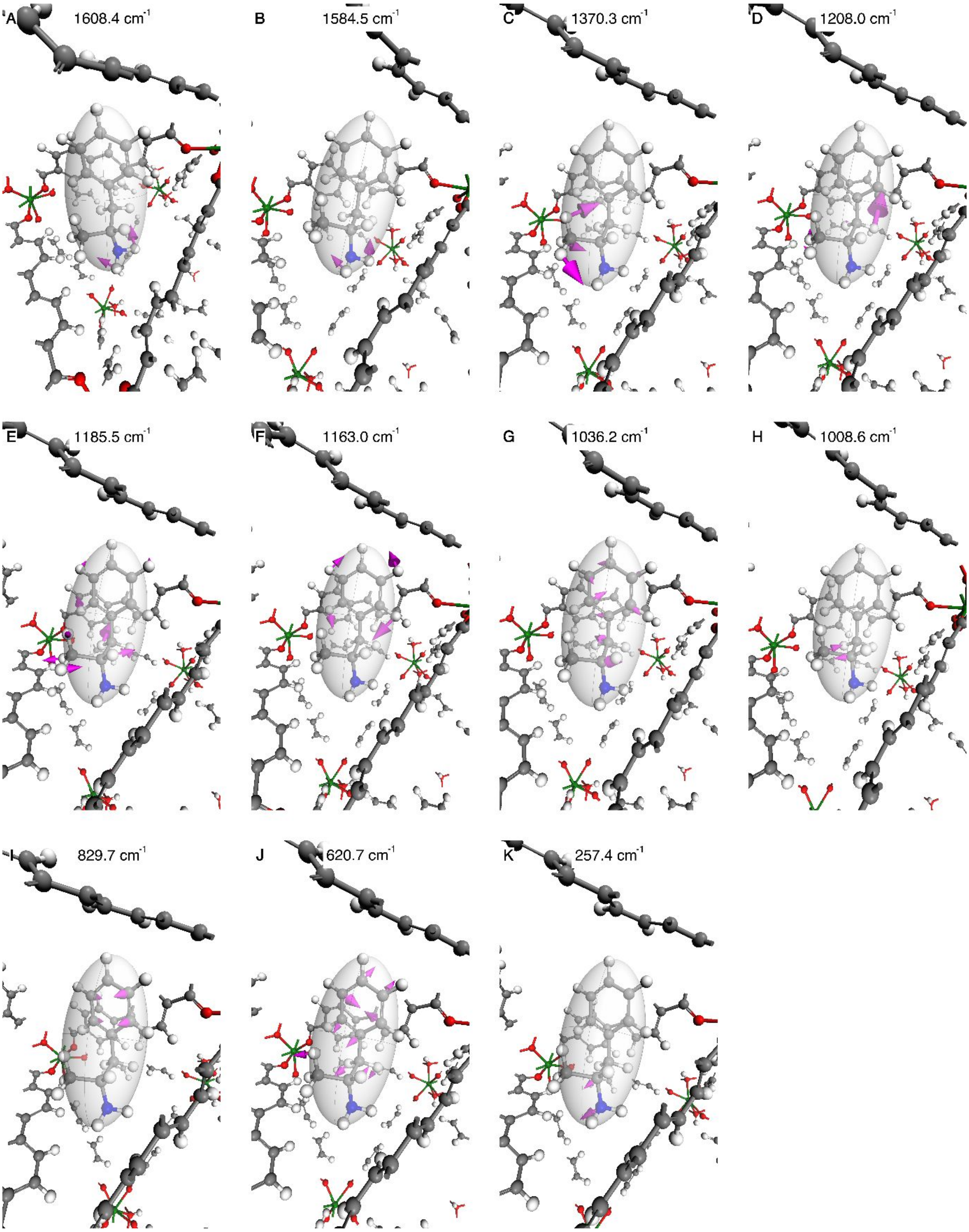

Figure S14. DFT optimised structures of AMP adsorbed in UiO-67 with vibrational eigenvectors depicted by arrows with lengths scaled by the Raman intensities; **(A)** deformation NH_2, **(B)** deformation NH_2, (C) deformation CH_3, **(D)** deformation CH_2, **(E)** bending CH, **(F)** bending CH, **(G)** aromatic ring and aliphatic chain CC stretch, **(H)** aliphatic chain CC stretch, **(I)** aromatic ring out-of-plane deformation, **(J)** aromatic ring CC and aliphatic chain CH deformation, **(K)** bending CN

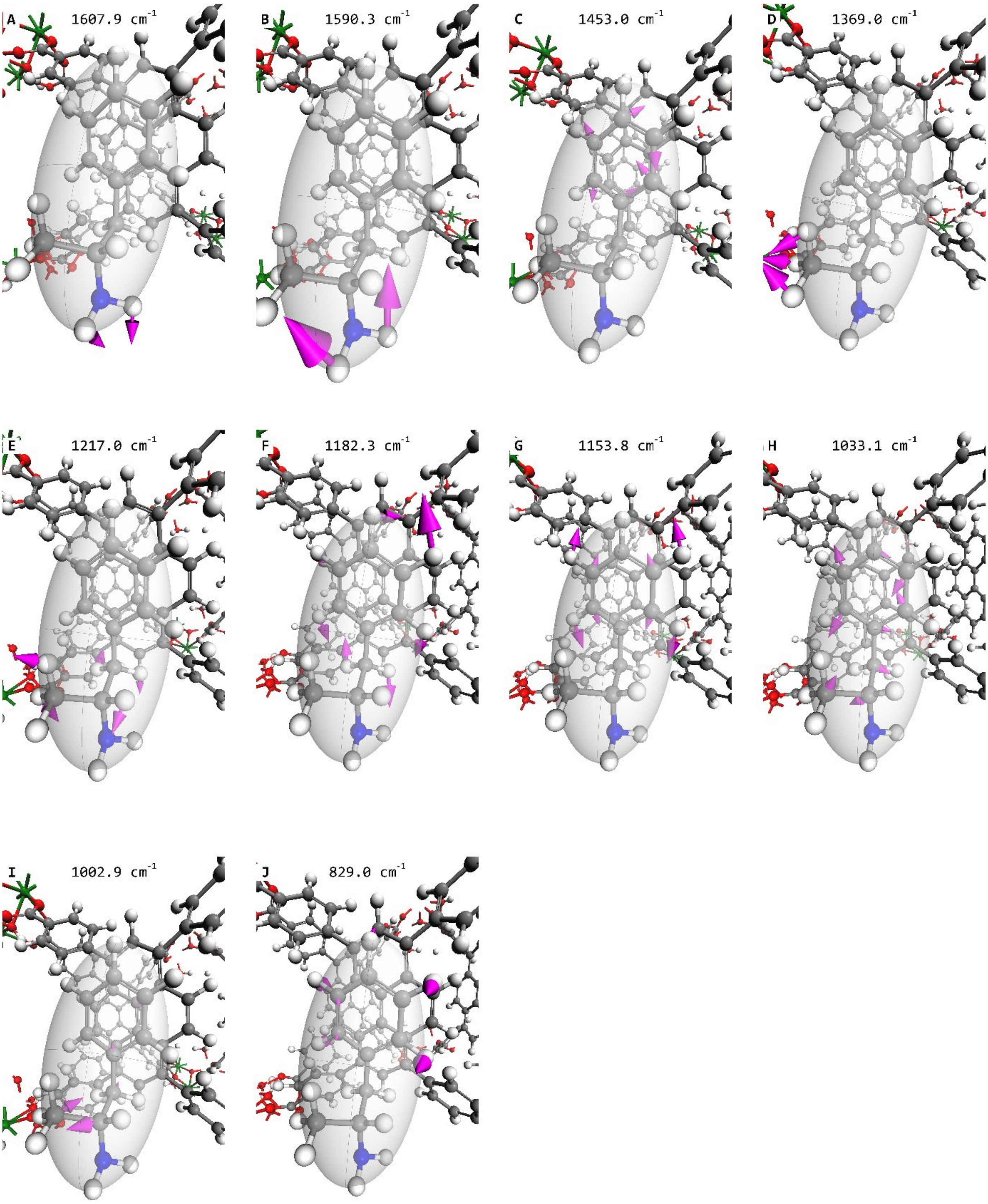

Figure S15. DFT optimised structures of AMP adsorbed in NU-1000 with vibrational eigenvectors depicted by arrows with lengths scaled by the Raman intensities; **(A)** deformation NH_2 **(B)** deformation NH_2, **(C)** aromatic ring in-plane CH bending, **(D)** deformation CH_3, **(E)** deformation CH_3 and CH_2, **(F)** bending CH, **(G)** bending CH, **(H)** aromatic ring and aliphatic chain CC stretch, **(I)** aromatic ring and aliphatic chain CC stretch, **(J)** aromatic ring out-of-plane deformation

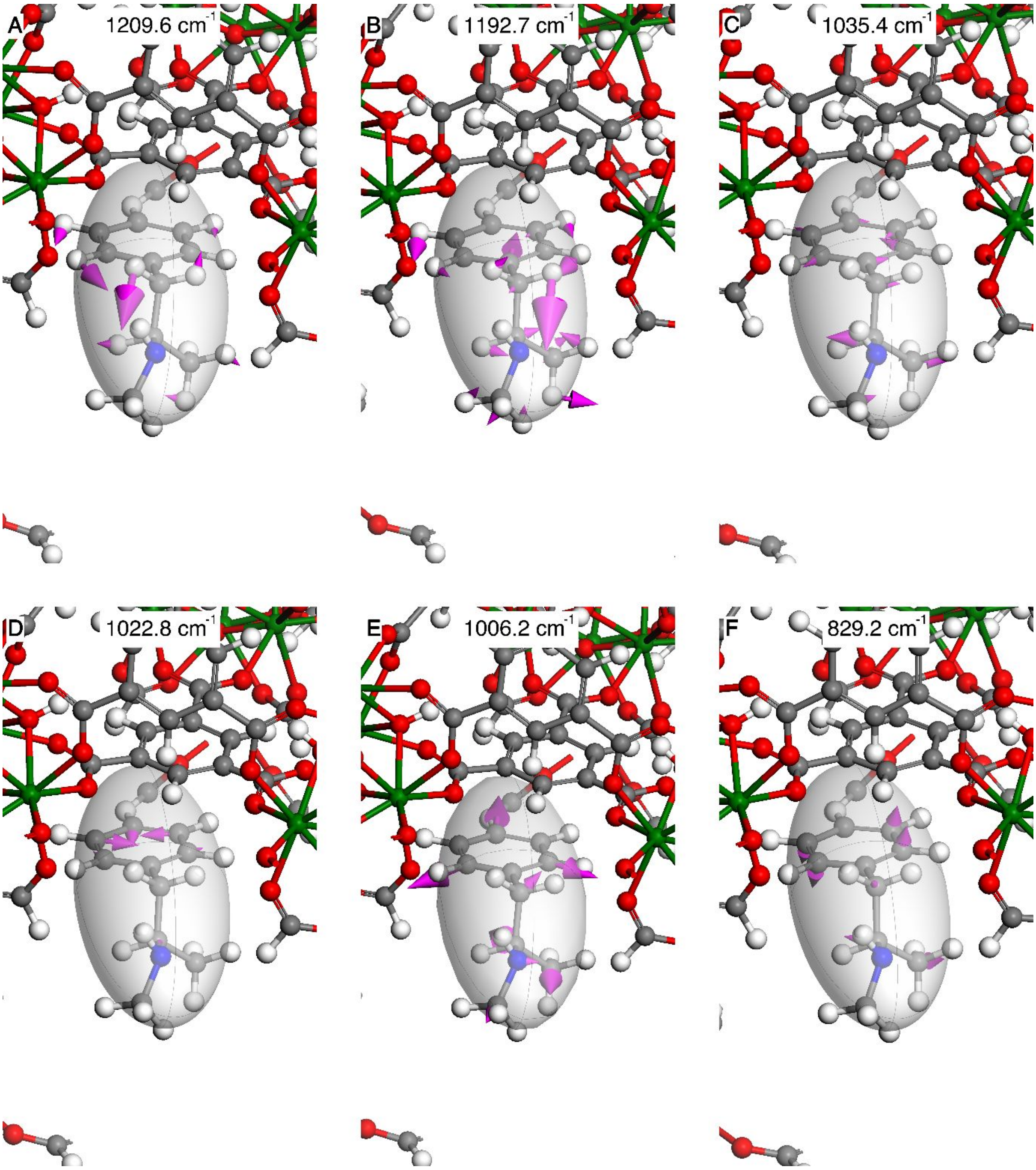

Figure S16. DFT optimised structures of mAMP adsorbed in MOF-808 with vibrational eigenvectors depicted by arrows with lengths scaled by the Raman intensities; **(A)** aromatic ring and aliphatic chain CH deformation, **(B)** CH bending, **(C)** aromatic ring and aliphatic chain CC stretching, **(D)** aromatic ring and aliphatic chain CC stretching, **(E)** aromatic ring and aliphatic chain CC stretching, **(F)** aromatic ring out-of-plane deformation

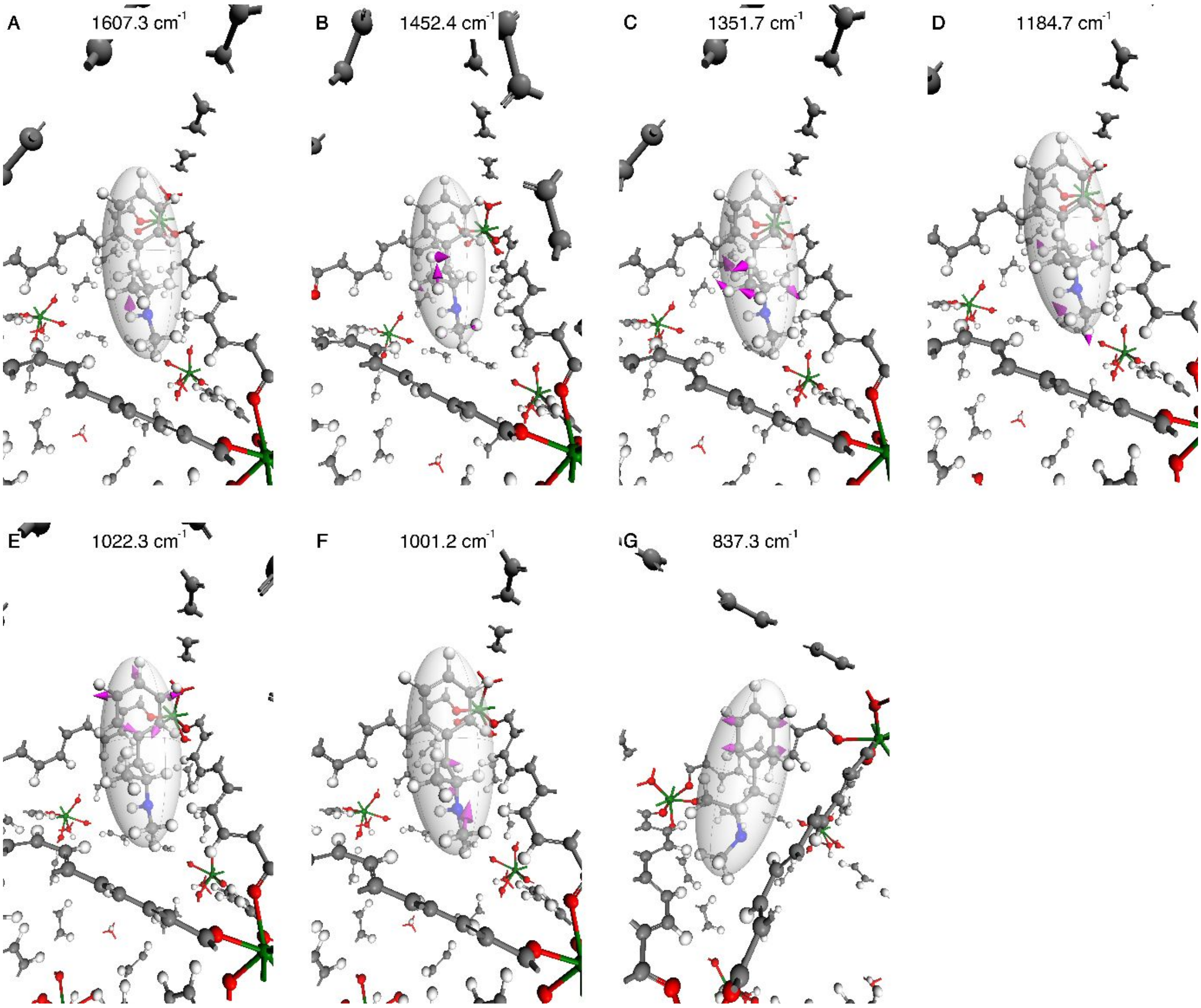

Figure S17. DFT optimised structures of mAMP adsorbed in UiO-67 with vibrational eigenvectors depicted by arrows with lengths scaled by the Raman intensities; **(A)** NH deformation, **(B)** CH bending, **(C)** aliphatic chain CH deformation, **(D)** CH bending, **(E)** aromatic ring CC stretching, **(F)** aliphatic chain CC stretching, **(G)** aromatic ring out-of-plane deformation

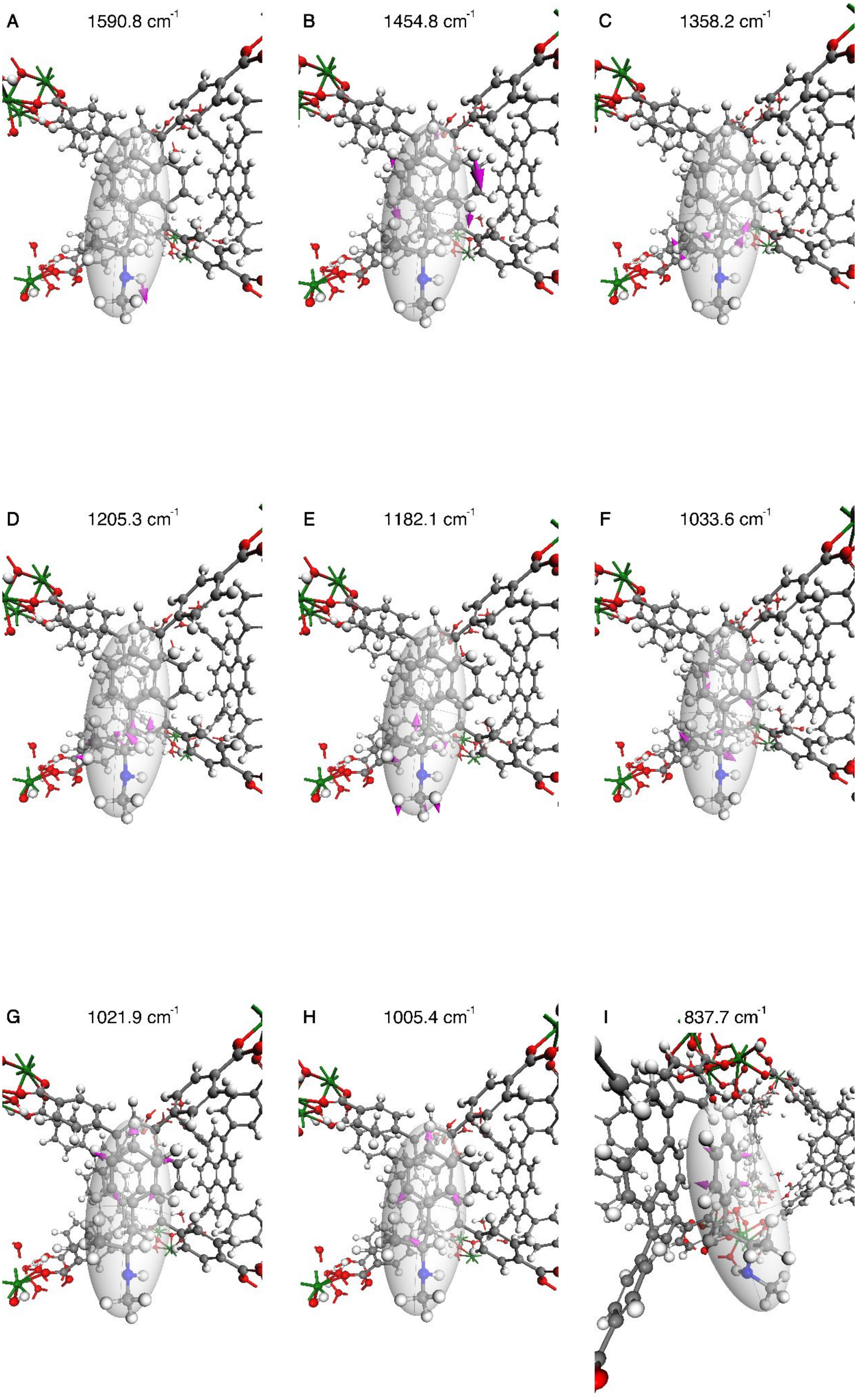
A
1590.8 cm-1
B
1454.8 cm-1
C
1358.2 cm-1
D
1205.3 cm-1
E
1182.1 cm-1
F
1033.6 cm-1
G
1021.9 cm-1
H
1005.4 cm-1
I
837.7 cm-1

Figure S18. DFT optimised structures of mAMP adsorbed in NU-1000 with vibrational eigenvectors depicted by arrows with lengths scaled by the Raman intensities; **(A)** NH deformation, **(B**) aromatic ring in-plane CH bending, **(C)** aliphatic chain CH deformation, **(D)** CH_3 CH_2 deformation, **(E)** bending CH, **(F)** aromatic ring and aliphatic chain CC stretching, **(G)** aromatic ring CC stretching, **(H)** aromatic ring and aliphatic chain CC stretching, **(I)** aromatic ring out-of-plane deformation

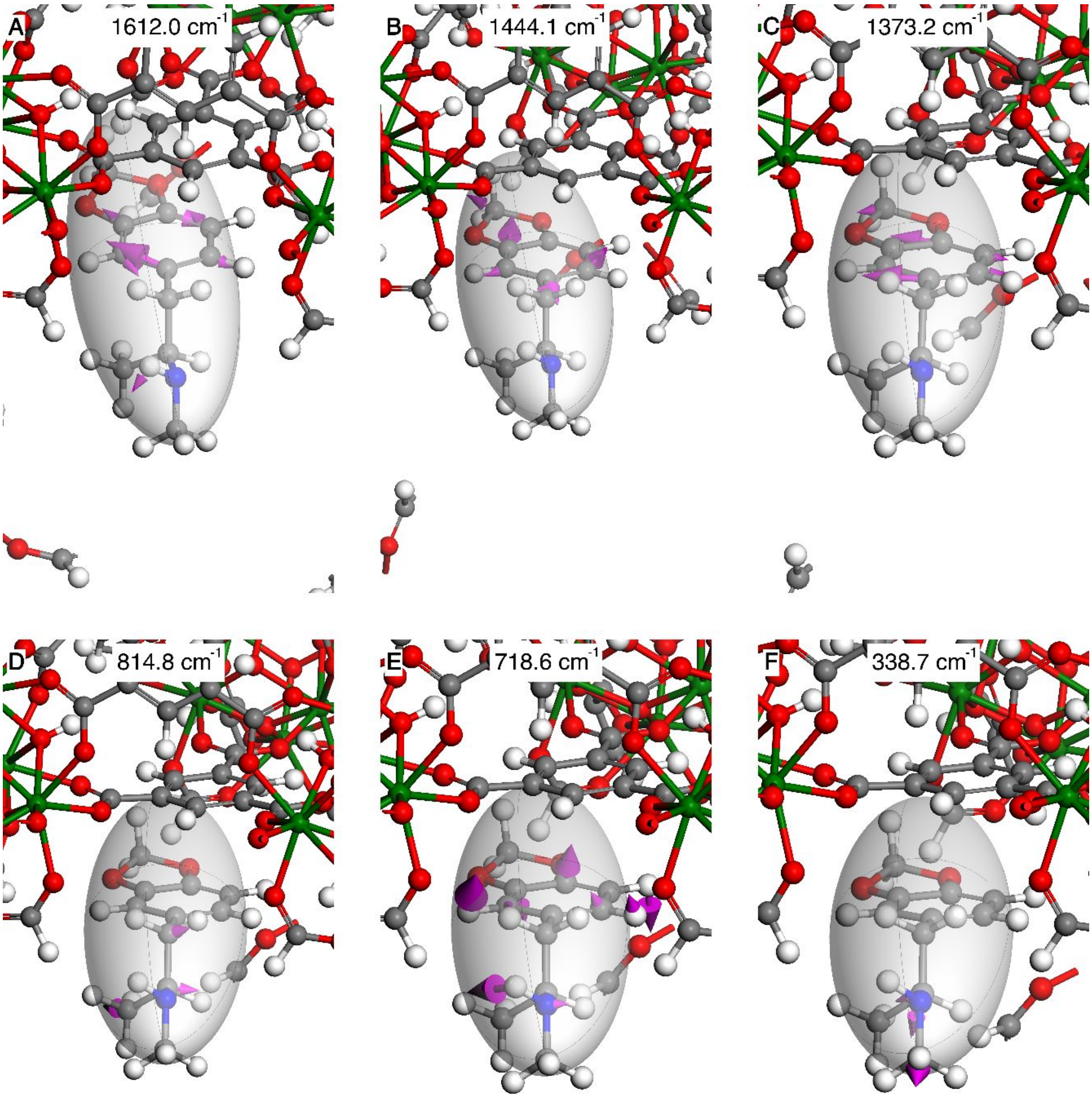

Figure S19. DFT optimised structures of MDMA adsorbed in MOF-808 with vibrational eigenvectors depicted by arrows with lengths scaled by the Raman intensities; **(A)** aromatic ring and NH vibration, **(B)** asymmetric and rocking CC, **(C)** asymmetric and rocking CC, **(D)** asymmetric and rocking CC, **(E)** aromatic ring and NH vibration, **(F)** C-N-C deformation

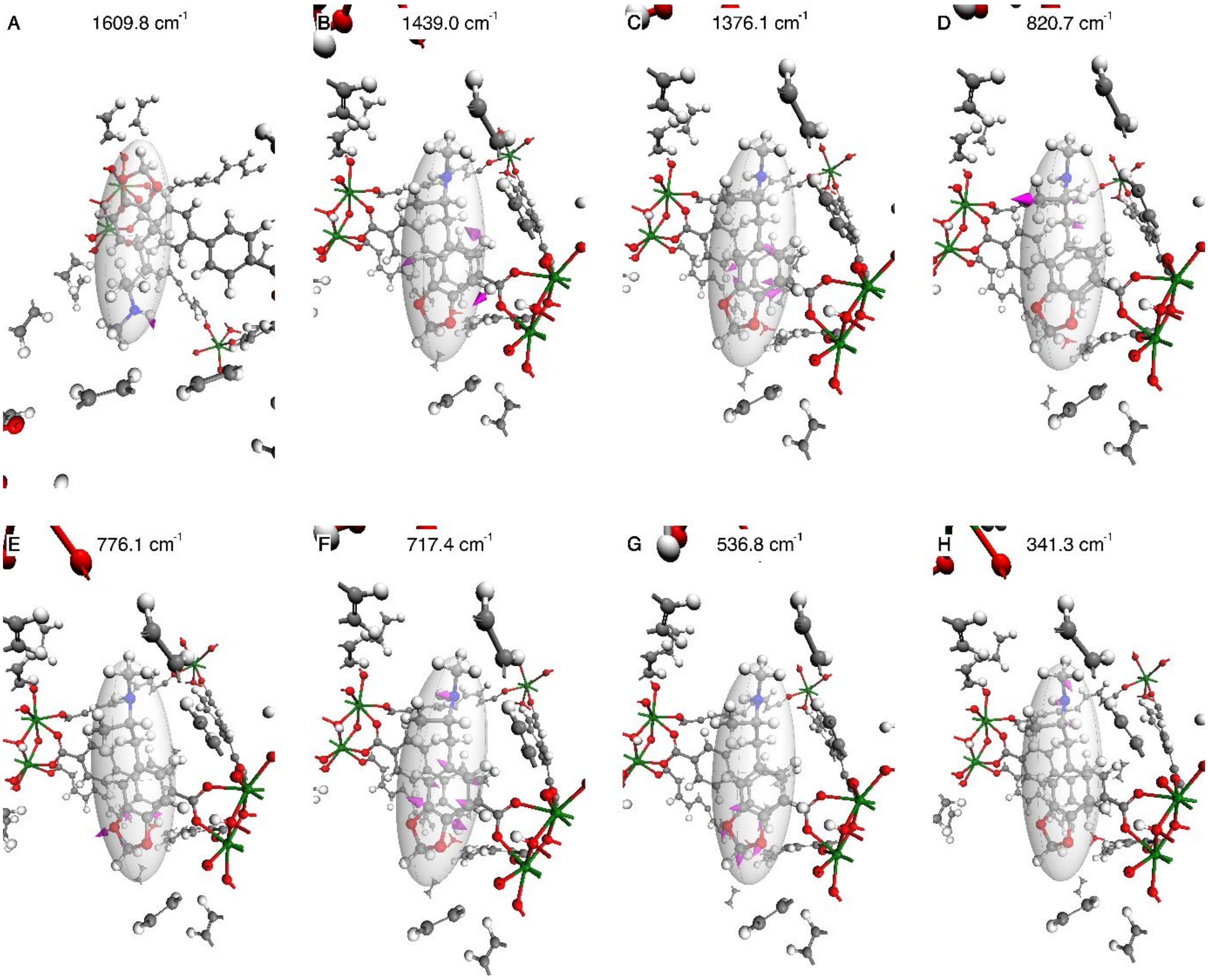

Figure S20. DFT optimised structures of MDMA adsorbed in UiO-67 with vibrational eigenvectors depicted by arrows with lengths scaled by the Raman intensities; **(A)** aromatic ring and NH vibration, **(B)** asymmetric and rocking CC, **(C)** asymmetric and rocking CC, **(D)** asymmetric and rocking CC, **(E)** C-O-C vibration, **(F)** aromatic ring and NH vibration, **(G)** C-O-C vibration, **(H)** C-N-C vibration

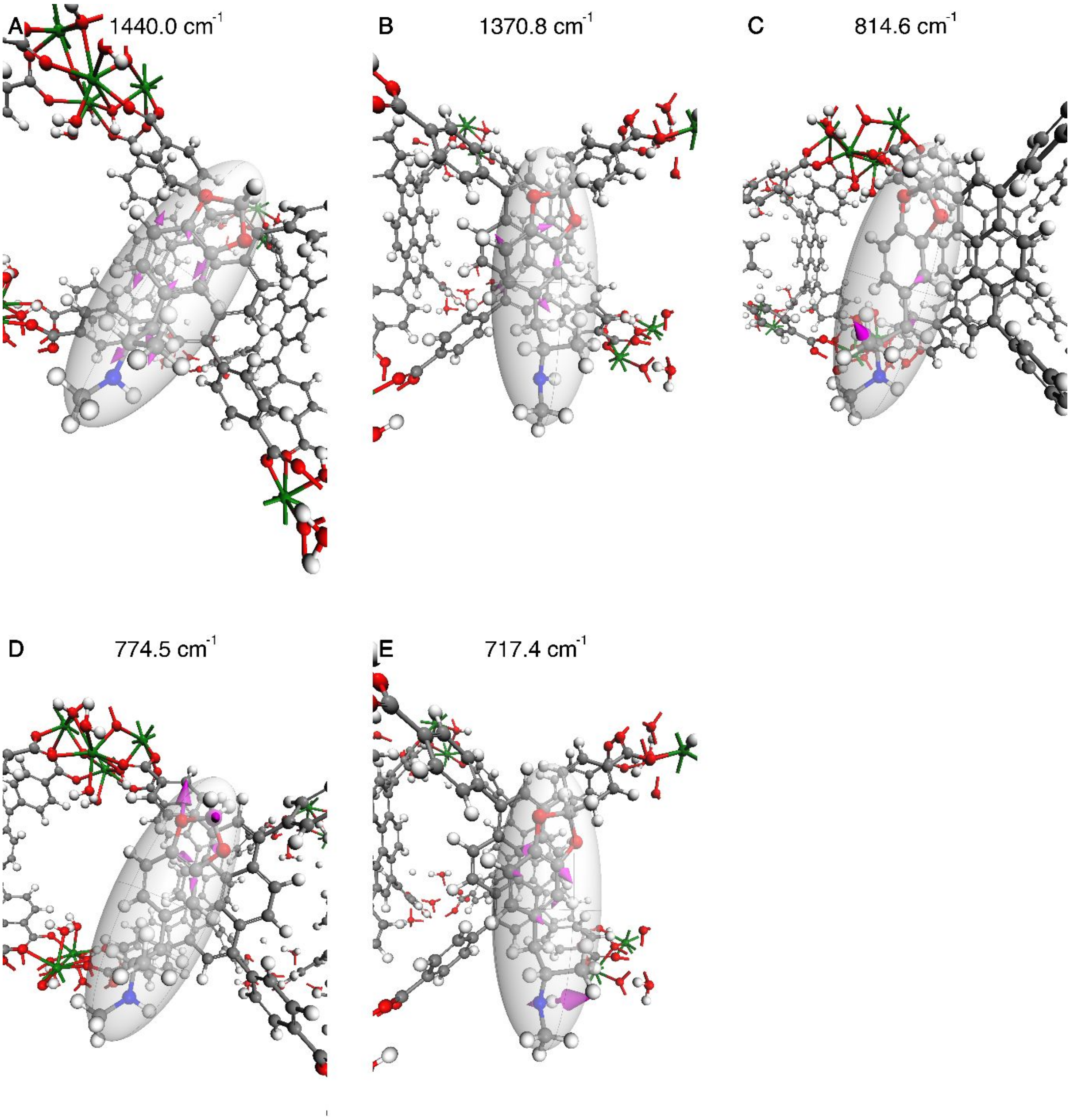

Figure S21. DFT optimised structures of MDMA adsorbed in NU-1000 with vibrational eigenvectors depicted by arrows with lengths scaled by the Raman intensities; **(A)** asymmetric and rocking CC, **(B)** asymmetric and rocking CC, **(C)** asymmetric and rocking CC, **(D)** C-O-C vibration, **(E)** aromatic ring and NH vibration

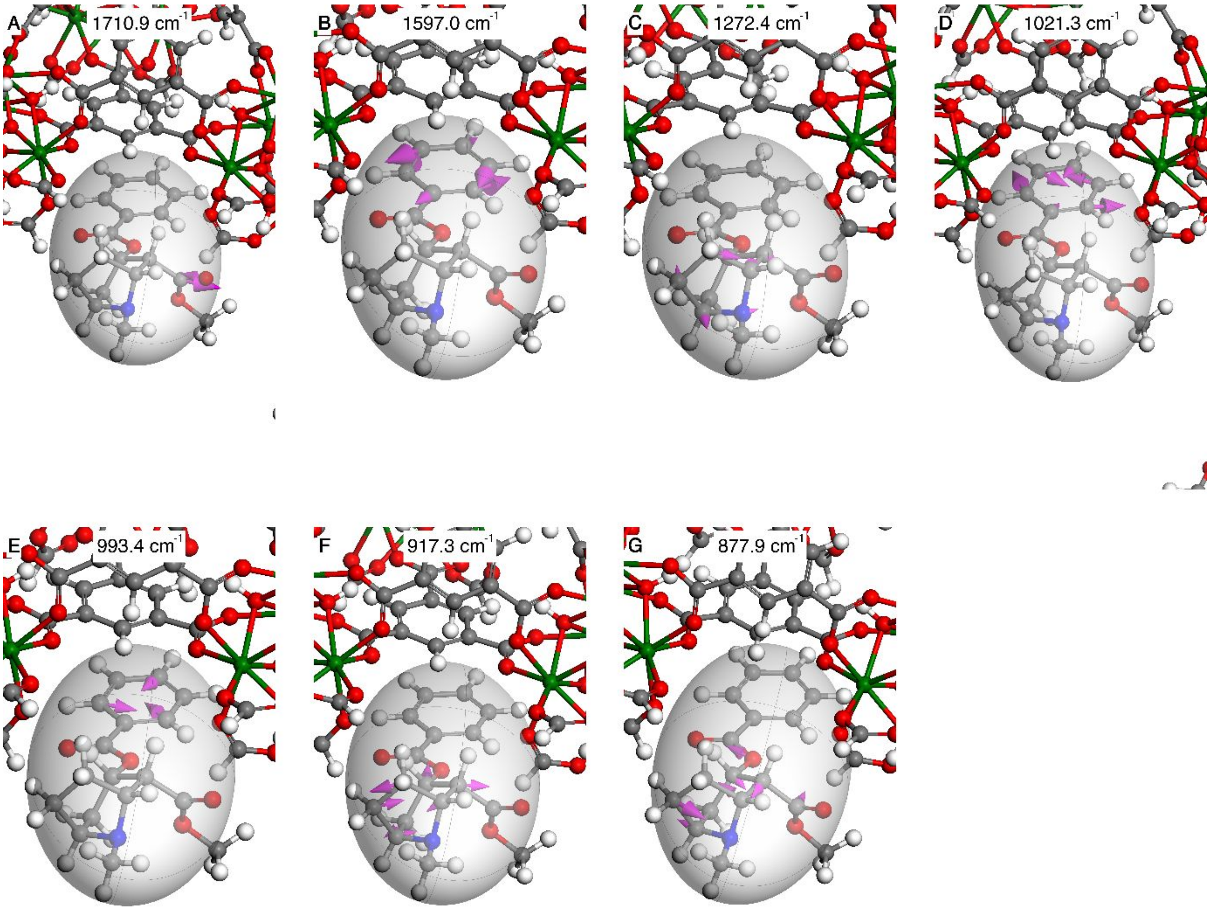

Figure S22. DFT optimised structures of COC adsorbed in MOF-808 with vibrational eigenvectors depicted by arrows with lengths scaled by the Raman intensities; **(A)** carbonyl C-O stretching, **(B)** aromatic ring CC stretching, **(C)** C-N stretching, **(D)** asymmetric aromatic ring breathing, **(E)** symmetric aromatic ring breathing, **(F)** tropane ring CC stretching, **(G)** tropane ring CC stretching

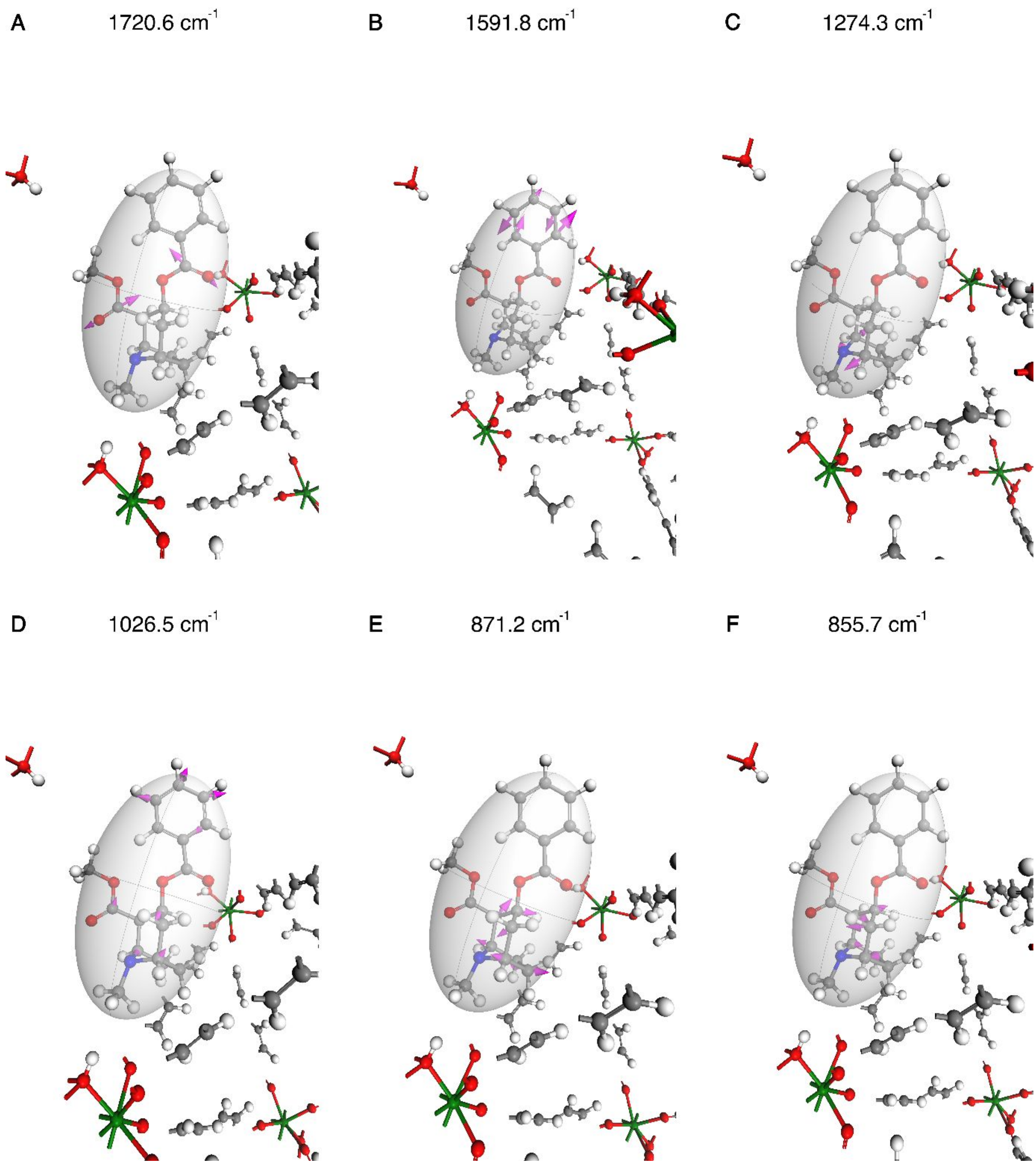

Figure S23. DFT optimised structures of COC adsorbed in UiO-67 with vibrational eigenvectors depicted by arrows with lengths scaled by the Raman intensities; **(A)** carbonyl C-O stretching, **(B)** aromatic ring CC stretching, **(C)** C-N stretching, **(D)** asymmetric aromatic ring breathing, **(E)** tropane ring CC stretching, **(F)** tropane ring CC stretching

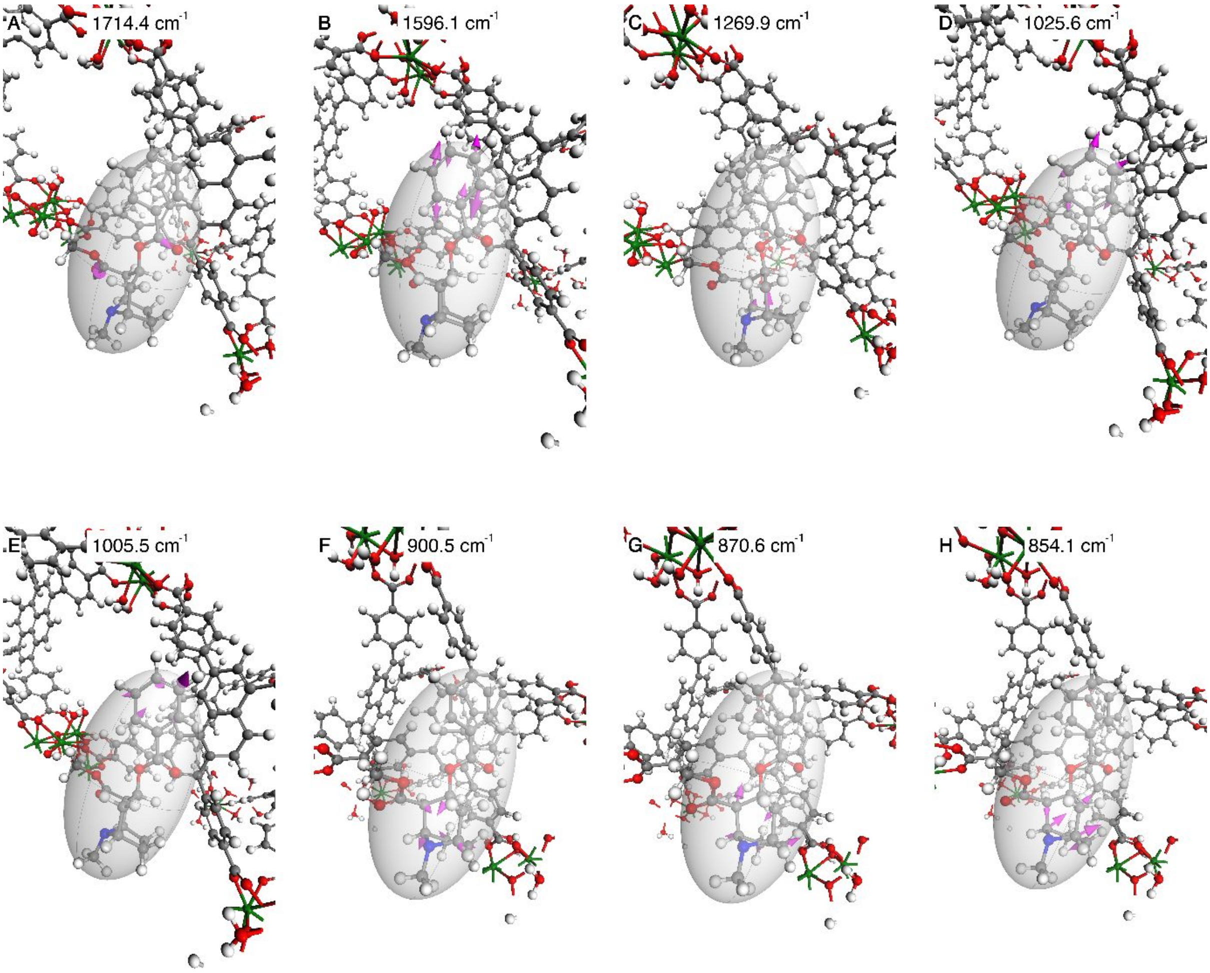

Figure S24. DFT optimised structures of COC adsorbed in NU-1000 with vibrational eigenvectors depicted by arrows with lengths scaled by the Raman intensities; **(A)** carbonyl C-O stretching, **(B)** aromatic ring CC stretching, **(C)** C-N stretching, **(D)** asymmetric aromatic ring breathing, **(E)** symmetric aromatic ring breathing, **(F)** tropane ring CC stretching, **(G)** tropane ring CC stretching, **(H)** tropane ring CC stretching **(F)**, **(G)**, **(H)**, **(I)**, **(J)**, **(K)**

DFT

The structures of AMP, mAMP, COC, and MDMA adsorbed in 3 studied MOF structures are described in the view of the π-π stacking of the aromatic rings of the organic adsorbates and the aromatic ring/rings of the MOF linkers.

Geometries

The definition of the distances and angles is given in Fig. S22, and their values obtained for the optimised structures are summarised in Table S5.

Table S5. Geometrical details for the π-π stacking in adsorbate molecule interaction with NU-1000, UiO-67, and MOF-808 frameworks. The angle α is a dihedral between planes of the rings. Definition of distances and β, γ angles are given in Fig. S22.

Adsorbate molecule	*d*(c(M)-c(L)) / Å	$d_\perp$(c(M)-P(L)) / Å	$d_\perp$(c(L)-P(M)) / Å	α / °	β / °	γ / °
**NU-1000**						
AMP	3.380	3.297	3.338	10.95	9.06	12.71
mAMP	3.458	3.425	3.389	5.83	11.36	7.86
COC	3.836	3.580	3.095	18.45	36.20	21.05
MDMA	3.687	3.508	3.396	7.96	22.93	17.93
**UiO-67**						
AMP	3.570	3.570	3.522	8.81	9.45	0.66
mAMP	3.750	3.734	3.534	17.94	19.58	5.42
COC, structure 1	3.597	3.586	3.462	12.36	15.80	4.50
COC, structure 2	*no π-π stacking*(*)					
COC, structure 3	*no π-π stacking*(*)					
MDMA	3.573	3.426	3.285	8.86	23.18	16.51
**MOF-808**						
AMP	3.504	3.335	3.286	8.35	20.32	17.87
mAMP	3.569	3.368	3.294	9.15	22.64	19.30
COC	3.559	3.337	3.426	6.38	15.75	20.37
MDMA	3.364	3.251	3.287	2.66	12.23	14.88

c(*x*) – centre of "*x*" ring
P(*x*) – plane fitted to the aromatic ring "*x*"
M – ring of mephedrone
L – ring of the MOF linker
* – mutual orientation of the aromatic rings indicates no π-π stacking hence the geometrical relations are not given

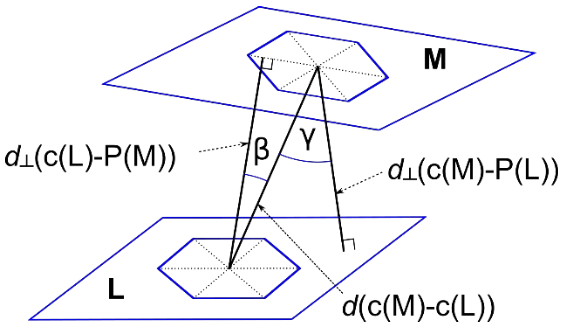

Figure S25. Sketch presenting the definition of the angles and distances whose values are shown in Table S5 (adapted from article [31]).

Energetics, charges and bond orders

The adsorption energies, obtained in a way accounting for the basis set incompleteness, together with the charges (two population analyses: Bader and DDEC6) and bond orders (DDEC6), are summarised in Table S6.

Table S6. Adsorption energies stemming from the π-π stacking between aromatic ring in organic adsorbate and aromatic rings in the MOF NU-1000, UiO-67, and MOF-808 linkers. The total bond order, *BO*(ads.-MOF), between the adsorbed molecule and the host MOF framework is determined by means of the DDEC6 bond analysis. The accumulated charge on the adsorbate molecule, q_{DDEC}(ads.) and q_{Bader}(ads.), calculated *via* the DDEC6 and the Bader partial charge analyses, respectively.

Adsorbate molecule	E_{ads} / eV	*BO*(ads.-MOF)	q_{DDEC}(ads.)	q_{Bader}(ads.)
		**NU-1000**		
AMP	-0.422	0.631	0.001	-0.005
mAMP	-0.433	0.594	-0.001	-0.003
COC	-0.955	0.955	-0.008	-0.019
MDMA	-0.543	0.694	0.011	-0.005
		**UiO-67**		
AMP	-0.530	0.692	-0.011	-0.004
mAMP	-0.698	0.963	-0.016	-0.011
COC, structure 1	-0.935	0.918	0.023	-0.005
COC, structure 2	-0.764	0.603	0.032	0.006
COC, structure 3	-0.966	0.790	-0.012	-0.015

MDMA	-0.659	0.815	0.017	-0.004
**MOF-808**				
AMP	-0.576	0.658	0.027	0.022
mAMP	-0.599	0.633	0.031	0.026
COC	-0.782	1.100	0.026	0.022
MDMA	-0.665	0.754	0.040	0.027

The equilibrium loading was estimated by means of the Monte-Carlo simulations in the temperature of 298 K, with the use of the Metropolis algorithm and with Universal Force Field[21]. The isotherms were modelled for the fugacity up to 100 kPa. The rigid host approximation was used and can rationalize the underestimation of the modelled loading for the MOF structures with tight channels.

The loading expressed in wt.% is shown in Table S7.

Table S7. Energetics of AMP, mAMP, COC, and MDMA sorption in studied MOF structures in dry and aqueous environments. The calculated loading $n_{molecules}$ was estimated per 1 unit cell of MOF.

Adsorbates	E_{ads} (vacuum) / eV	E_{ads} (water)* / eV	$n_{molecules}$** (calculated)
**NU-1000**			
AMP	-0.422	-0.413	55
mAMP	-0.433	-0.447	38
COC	-0.955	-0.842	23
MDMA	-0.543	-0.517	38
**UiO-67**			
AMP	-0.530	-0.434	9
mAMP	-0.698	-0.353	7
COC, structure 1	-0.935	-0.871	3
COC, structure 2	-0.764	-0.640	
COC, structure 3	-0.966	-1.012	
MDMA	-0.659	-0.547	6
**MOF-808**			

AMP	-0.576	-0.488	24
mAMP	-0.599	-0.504	21
COC	-0.782	-0.514	10
MDMA	-0.665	-0.544	17
* calculations performed with polarisable continuum with $\varepsilon = 80$.<br>** calculated within the rigid host approximation<br>*** values calculated from the experimental %wt			

Accessible Solvent Surface

For modelling of the Accessible Solvent Surface, the following parameters have been used: initial solvent radius of 1.4 Å, and the maximal solvent radius of 2.0 Å. The results are summarised in Table S8.

Table S8. The values of the Accessible Solvent Surface and void fraction for the studied MOF models.

Occupied volume / $Å^3$	Free volume / $Å^3$	Cell volume / $Å^3$	Void fraction	Surface area / $Å^2$
**NU-1000**				
9790.37	13166.32	22956.70	0.574	3632.26
**UiO-67**				
3162.45	1696.49	4858.93	0.349	1130.28
**MOF-808**				
5510.26	5469.64	10979.90	0.498	1794.31

Structures

In the following figures, the relevant parts of the MOF structures are presented with aromatic ring/rings involved in the π-π stacking marked in black. All H atoms removed for clarity.

NU-1000

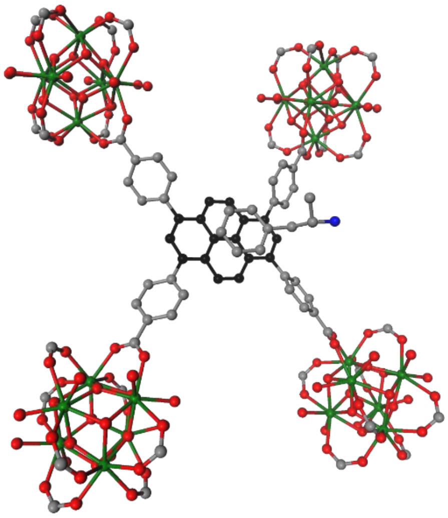

Figure S26. AMP@NU1000. Colour coding: gray: C, blue: N, red: O, green: Zr, black: aromatic rings involved in the π-π stacking.

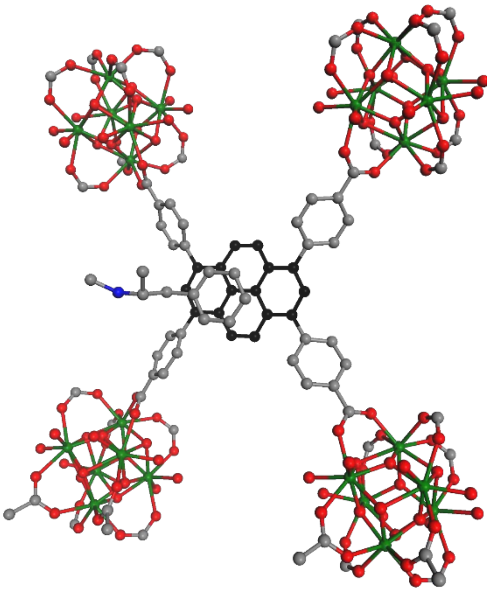

Figure S27. mAMP@NU1000. Colour coding: gray: C, blue: N, red: O, green: Zr, black: aromatic rings involved in the π-π stacking.

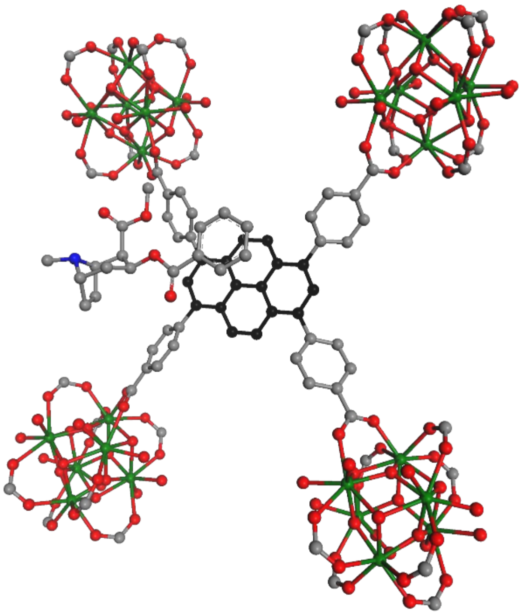

Figure S28. COC@NU1000. Colour coding: gray: C, blue: N, red: O, green: Zr, black: aromatic rings involved in the π-π stacking.

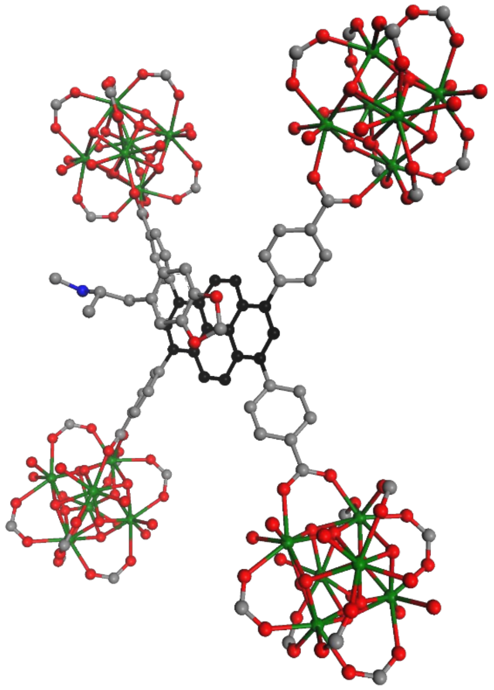

Figure S29. MDMA@NU1000. Colour coding: gray: C, blue: N, red: O, green: Zr, black: aromatic rings involved in the π-π stacking.

UiO-67

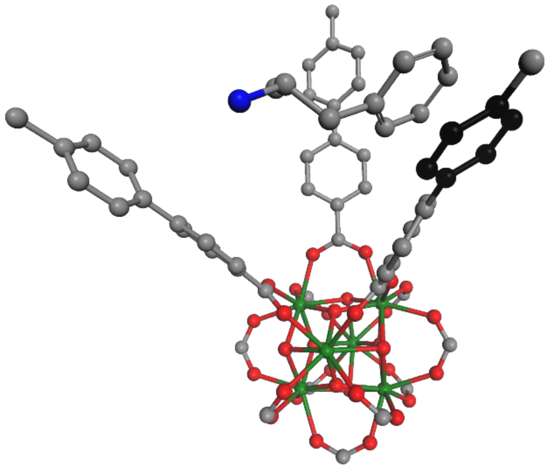

Figure S30. AMP@UiO-67. Colour coding: gray: C, blue: N, red: O, green: Zr, black: aromatic ring involved in the π-π stacking.

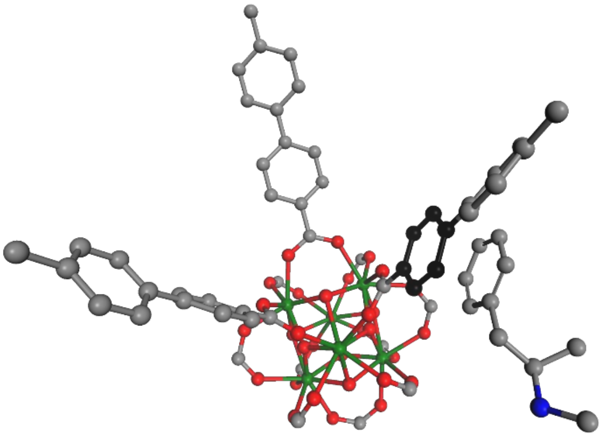

Figure S31. mAMP@UiO-67. Colour coding: gray: C, blue: N, red: O, green: Zr, black: aromatic ring involved in the π-π stacking.

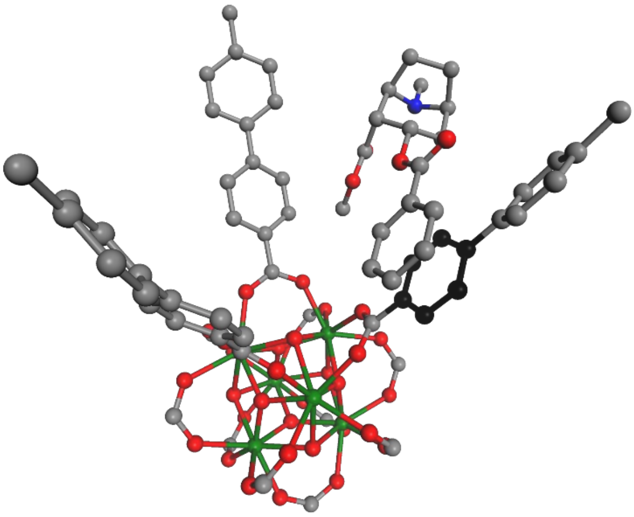

Figure S32. COC@UiO-67, structure 1. Colour coding: gray: C, blue: N, red: O, green: Zr, black: aromatic ring involved in the π-π stacking.

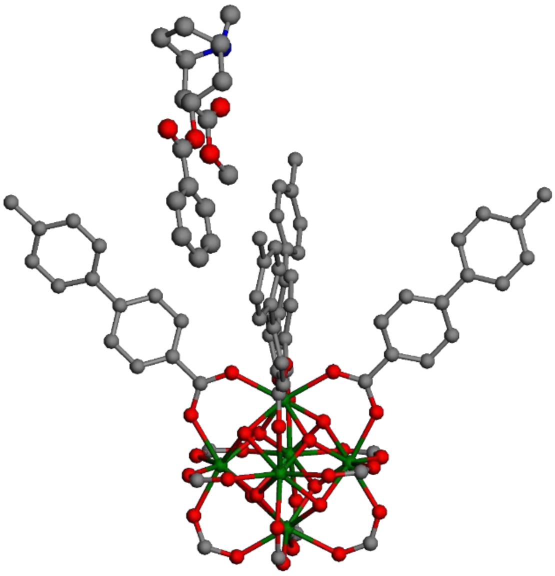

Figure S33. COC@UiO-67, structure 2. Colour coding: gray: C, blue: N, red: O, green: Zr. No π-π stacking is observed.

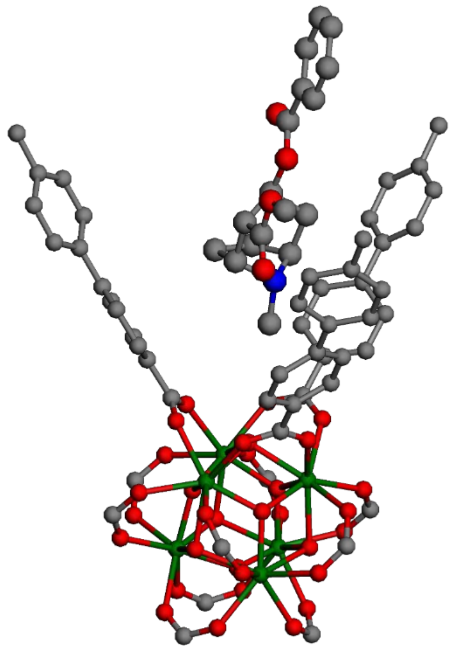

Figure S34. COC@UiO-67, structure 3. Colour coding: gray: C, blue: N, red: O, green: Zr. No π-π stacking is observed.

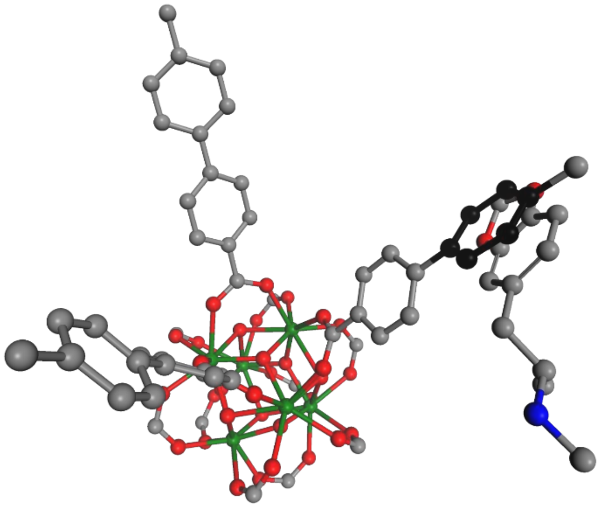

Figure S35. MDMA@UiO-67. Colour coding: gray: C, blue: N, red: O, green: Zr, black: aromatic ring involved in the π-π stacking.

MOF-808

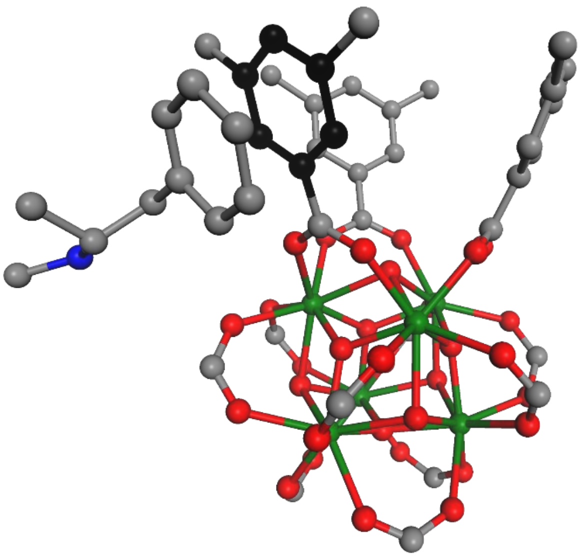

Figure S36. mAMP@MOF-808. Colour coding: gray: C, blue: N, red: O, green: Zr, black: aromatic ring involved in the π-π stacking.

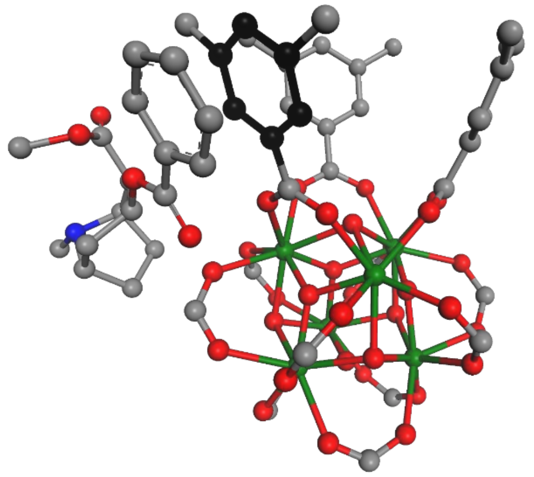

Figure S37. COC@MOF-808. Colour coding: gray: C, blue: N, red: O, green: Zr, black: aromatic ring involved in the π-π stacking.

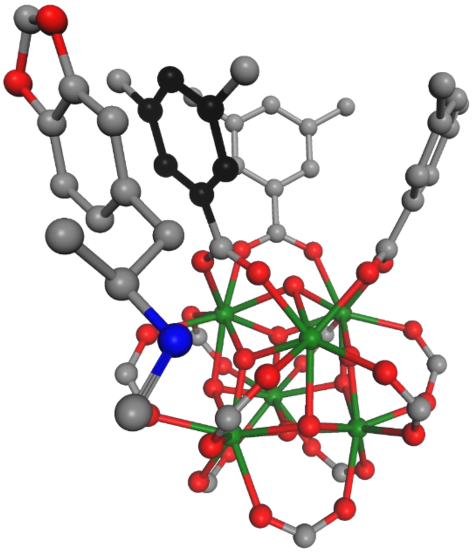

Figure S38. MDMA@MOF-808. Colour coding: gray: C, blue: N, red: O, green: Zr, black: aromatic ring involved in the π-π stacking.

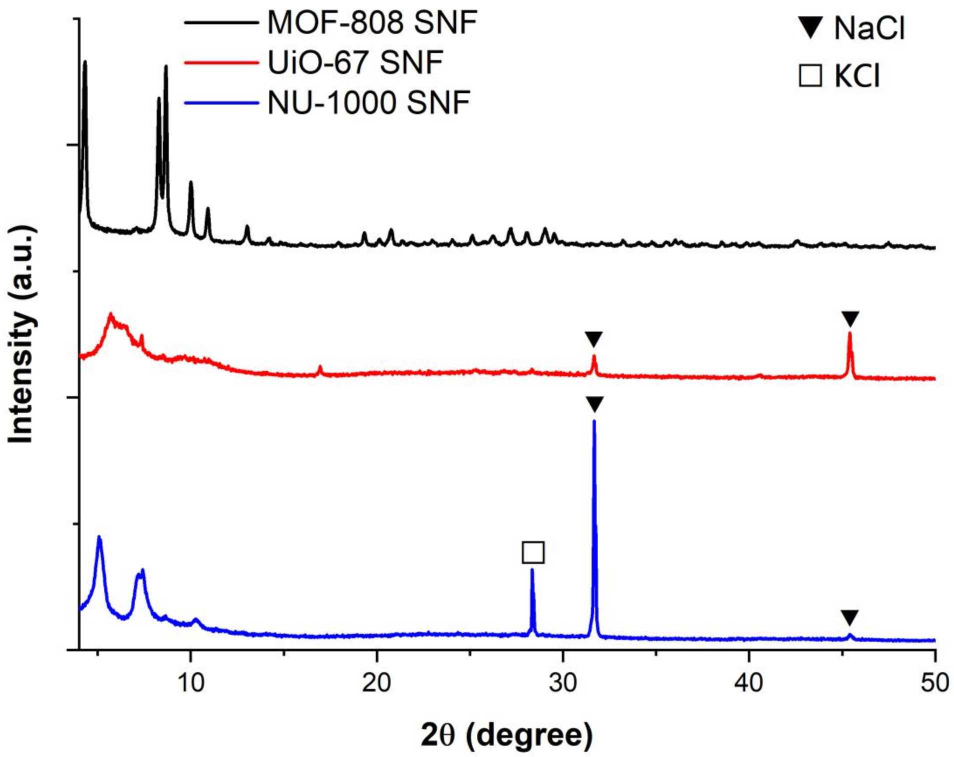

Figure S39. PXRD of selected MOFs after stability test in SNF solution

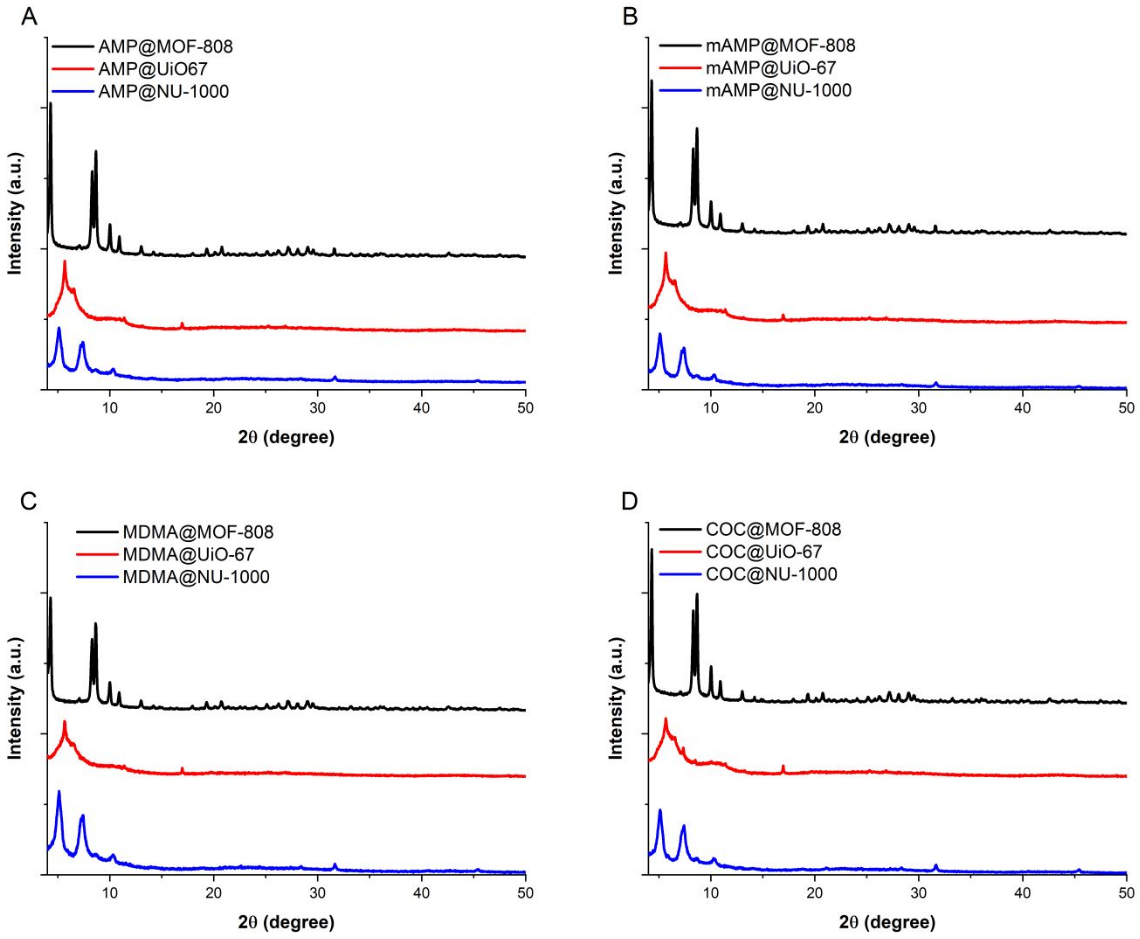

Figure S40. PXRD analysis for MOF samples after adsorption of drugs of abuse: **(A)** AMP; **(B)** mAMP; **(C)** MDMA; **(D)** COC

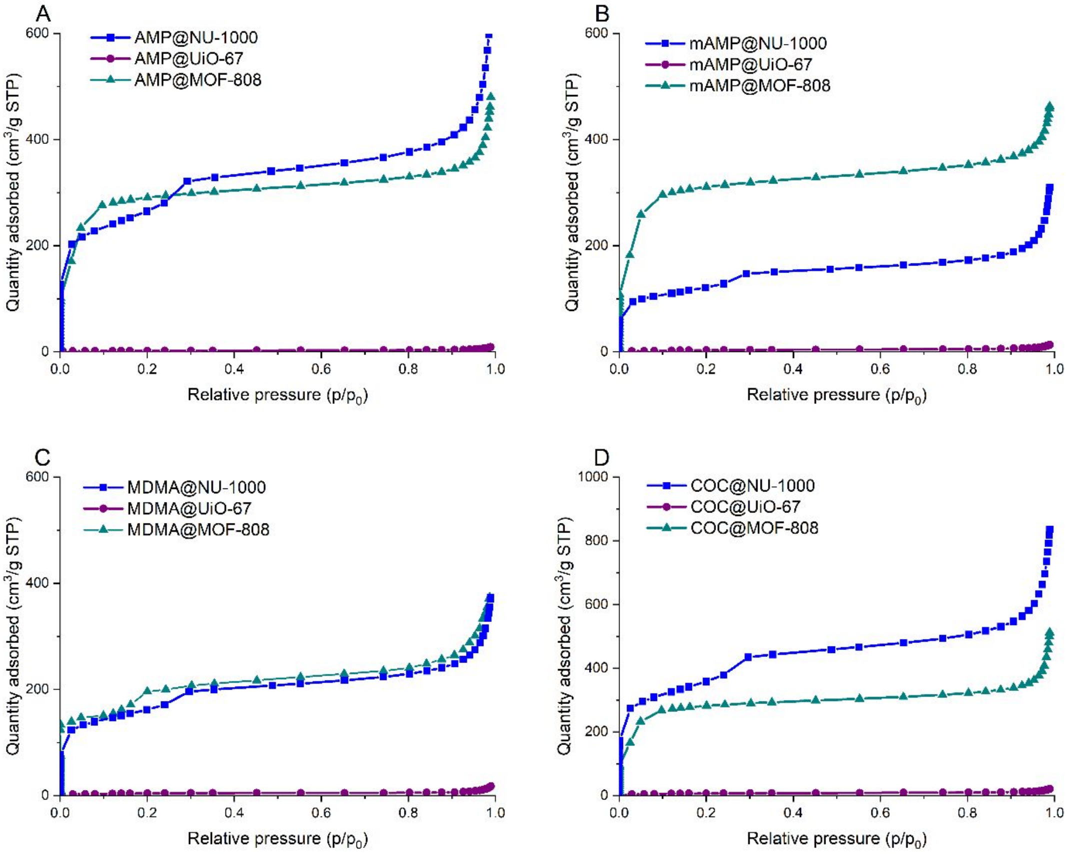

Figure S41. N_2 adsorption isotherms for MOFs after the adsorption of selected drug of abuse; **(A)** AMP; **(B)** mAMP; **(C)** MDMA; **(D)** COC

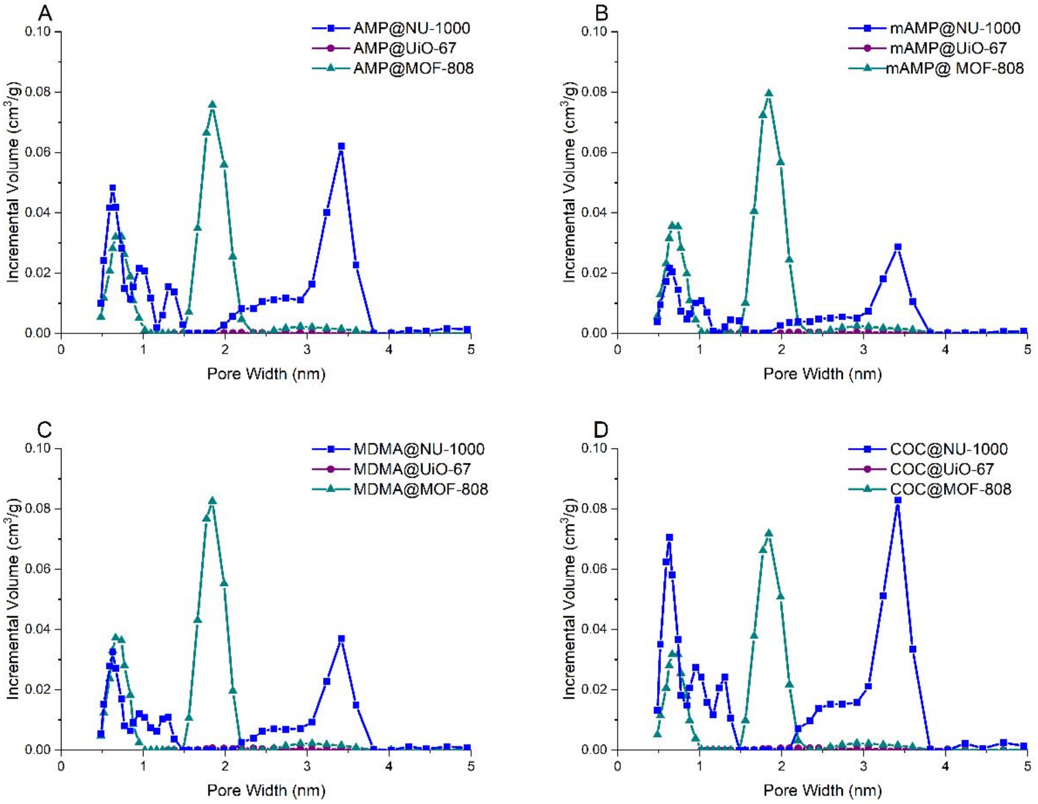

Figure S42. Pore size distribution (PSD) for MOFs after the adsorption of selected drug of abuse; **(A)** AMP; **(B)** mAMP; **(C)** MDMA; **(D)** COC

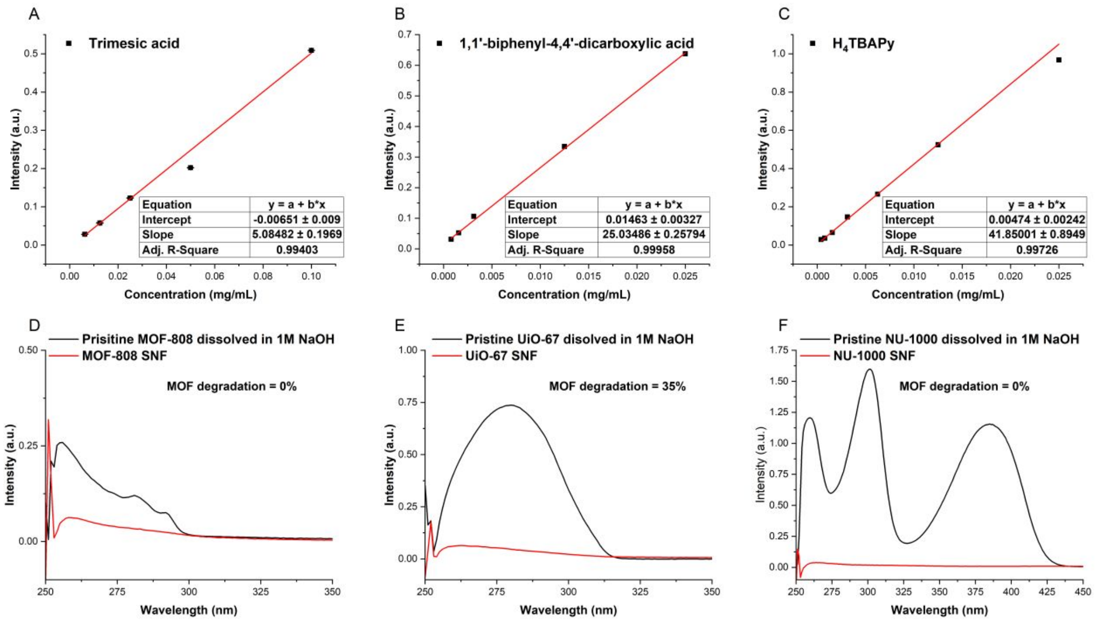

Figure S43. UV-Vis spectra of organic linkers upon their release in SNF medium; UV-Vis calibration curves for: **(A)** trimesic acid, **(B)** 1,1'-biphenyl-4,4'-dicarboxylic acid, **(C)** H_4TBAPy; Organic linker release for pristine MOF dissolved in 1M NaOH (upper spectra) and SNF solution after 24h MOF soaking test **(D)** MOF-808, **(E)** UiO-67, **(F)** NU-1000

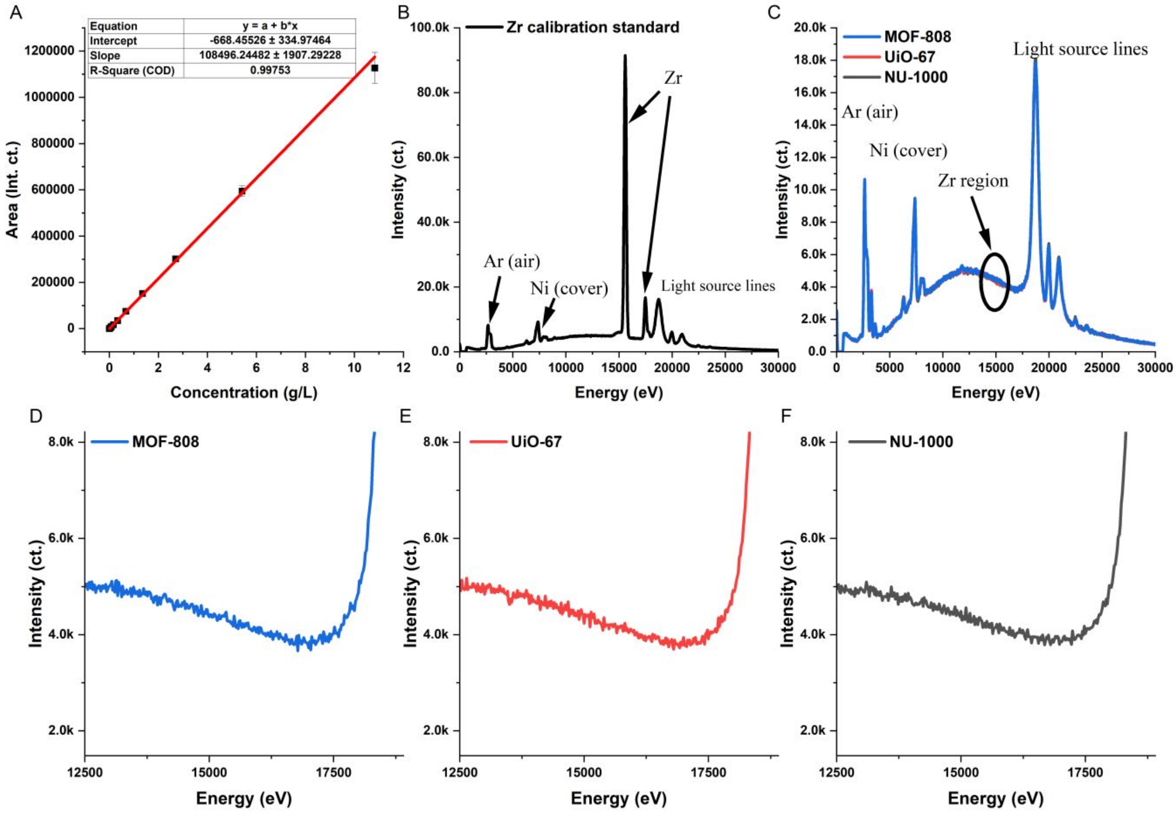

Figure S44. XRF spectroscopy results for determination of Zr in MOF soaking test in SNF solution; **(A)** XRF calibration curve, **(B)** Zr signal from calibration standard, **(C)** filtrate spectrum after MOF soaking test in SNF solution, **(D)** filtrate spectrum after MOF-808 soaking test in SNF solution, **(E)** filtrate spectrum after UiO-67 soaking test in SNF solution, **(F)** filtrate spectrum after NU-1000 soaking test in SNF solution

In vivo

Table S9. One-way ANOVA analysis the effect of AMP (5 µM, 25 µM, 50 µM, 100 µM, 150 µM, 250 µM, 500 µM, and 750 µM) on heart rate measured in 1 min, and average distance (cm) moved by zebrafish larvae during 10 min light phase

Observed parameter	$F(d,f)$	P
heart rate	$F(8, 92) = 23.82$	< 0.0001
locomotor activity	$F(8, 92) = 10.30$	< 0.0001

Table S10. Two-way ANOVA analysis of AMP effects (100 µM) on average distance (cm) moved by zebrafish larvae during 10 min light phase

	Locomotor (100 µM)	
	$F(d,f)$	P
Interaction	$F(3, 84) = 4.067$	$= 0.0095$
MOFs treatment	$F(3, 84) = 0.3503$	$= 0.7890$
AMP treatment	$F(1, 84) = 5.549$	$= 0.0208$

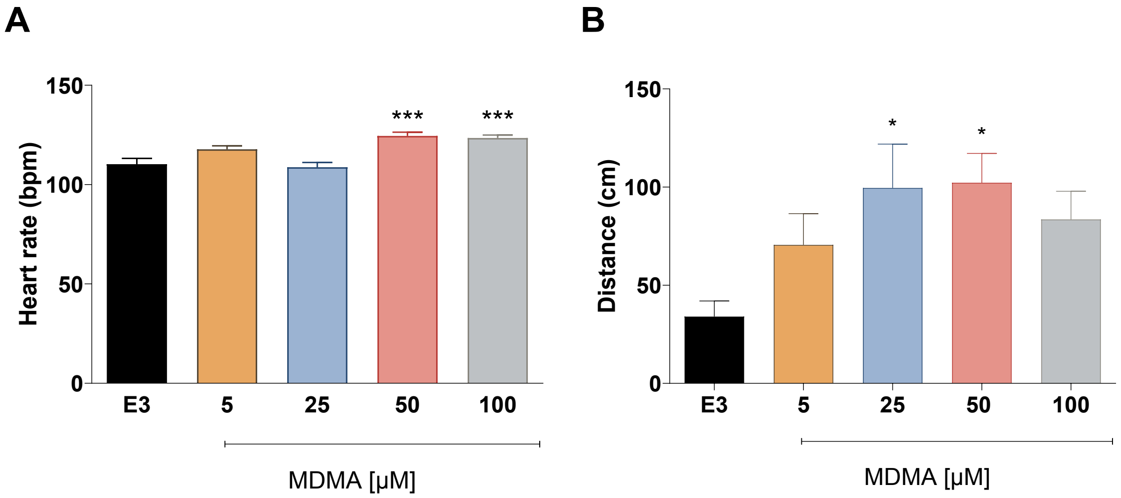

Figure S45. The effect MDMA (5 µM, 25 µM, 50 µM, and 100 µM) on **(A)** heart rate measured in 1 min (beat per minute, bpm), and **(B)** average distance (cm) moved by zebrafish larvae during 10 min light phase. Data are presented as mean ± SEM, $n = 12$, $*p < 0.05$, $***p < 0.001$ in comparison to the control group, post hoc Tukey's test

Table S11. One-way ANOVA analysis of effect MDMA (5 µM, 25 µM, 50 µM, and 100 µM) on heart rate measured in 1 min, and average distance (cm) moved by zebrafish larvae during 10 min light phase

Observed parameter	$F(d,f)$	P
heart rate	$F(4, 34) = 11.03$	< 0.0001
locomotor activity	$F(4, 52) = 3.934$	$= 0.0248$

Table S12. Two-way ANOVA analysis of effect MDMA (50 µM) on heart rate measured in 1 min

	Heartbeat (50 µM)	
	$F(d,f)$	P
Interaction	$F(3, 83) = 5.285$	$= 0.0022$
MOFs treatment	$F(3, 83) = 0.5642$	$= 0.6402$
MDMA treatment	$F(1, 83) = 0.7696$	$= 0.3829$

Table S13. Two-way ANOVA analysis of effect MDMA (50 µM) on heart rate measured in 1 min

	Heartbeat (50 µM)	
	F(*d*,*f*)	*P*
Interaction	*F*(3, 62) = 3.584	= 0.0186
MOFs treatment	*F*(3, 62) = 3.204	= 0.0292
MDMA treatment	*F*(1, 62) = 0.2690	= 0.6058

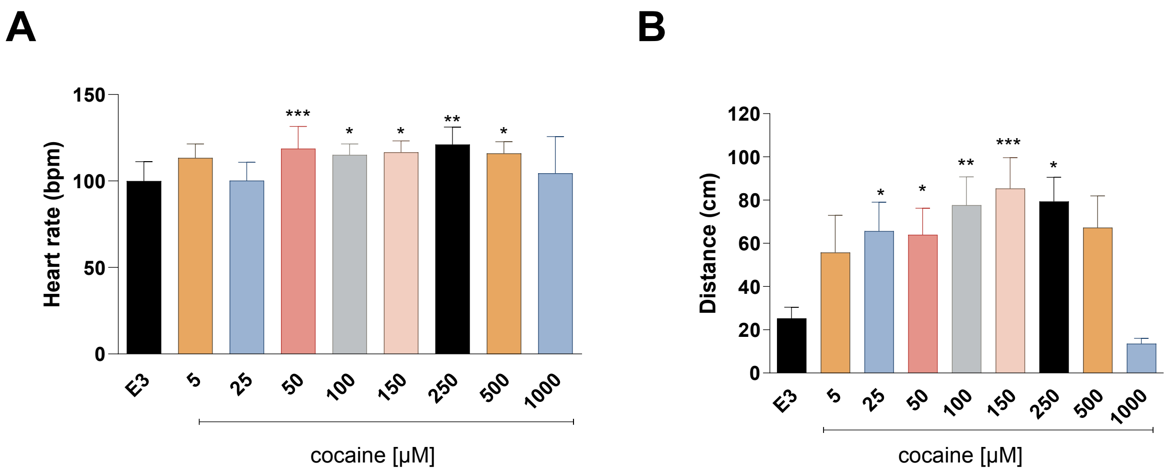

Figure S46. The effect of COC (5 µM, 25 µM, 50 µM, 100 µM, 150 µM, 250 µM, 500 µM, and 1000 µM) on **(A)** heart rate measured in 1 min and **(B)** average distance (cm) moved by zebrafish larvae during 10 min light phase. Data are presented as mean ± SEM, $n = 12$, $*p < 0.05$, $**p < 0.01$, $***p < 0.001$ in comparison to the control group, post hoc Tukey's test

Table S14. One-way ANOVA analysis of effect COC (5 µM, 25 µM, 50 µM, 100 µM, 150 µM, 250 µM, 500 µM, and 1000 µM) on heart rate measured in 1 min, and average distance (cm) moved by zebrafish larvae during 10 min light phase

Observed parameter	*F(d,f)*	*P*
Heartbeat	*F*(8, 98) = 5.112	< 0.0001
Locomotor activity	*F*(4, 98) = 4.652	= 0.0011

Table S15. Two-way ANOVA analysis of COC effects (50 µM, 100 µM) on heart rate measured in 1 min

	Heartbeat (50 µM)		Heartbeat (100 µM)	
	F(d,f)	*P*	*F(d,f)*	*P*
Interaction	$F(3, 96) = 3.710$	= 0.0150	$F(3, 96) = 4.292$	= 0.0150
MOFs treatment	$F(3, 96) = 3.878$	= 0.0123	$F(3, 96) = 2.880$	= 0.0123
COC treatment	$F(1, 96) = 7.914$	= 0.0062	$F(1, 96) = 1.616$	= 0.0062

Table S16. Two-way ANOVA analysis of COC effects (50 µM, 100 µM) on average distance (cm) moved by zebrafish larvae during 10 min light

	Locomotor activity (50 µM)		Locomotor activity (100 µM)	
	F(d,f)	*P*	*F(d,f)*	*P*
Interaction	$F(3, 94) = 21.68$	< 0.0001	$F(3, 91) = 8.050$	< 0.0001
MOFs treatment	$F(3, 94) = 0.2717$	= 0.8457	$F(3, 91) = 2.824$	= 0.0431
COC treatment	$F(1, 94) = 41.31$	< 0.0001	$F(1, 91) = 1.602$	= 0.2088

Bibliography

(1) Kresse, G.; Furthmüller, J. Efficiency of Ab-Initio Total Energy Calculations for Metals and Semiconductors Using a Plane-Wave Basis Set. *Comput. Mater. Sci.* **1996**, *6* (1), 15–50. https://doi.org/10.1016/0927-0256(96)00008-0.

(2) Kresse, G.; Hafner, J. Ab Initio Molecular Dynamics for Open-Shell Transition Metals. *Phys. Rev. B* **1993**, *48* (17), 13115–13118. https://doi.org/10.1103/PhysRevB.48.13115.

(3) Blöchl, P. E. Projector Augmented-Wave Method. *Phys. Rev. B* **1994**, *50* (24), 17953–17979. https://doi.org/10.1103/PhysRevB.50.17953.

(4) Kresse, G.; Joubert, D. From Ultrasoft Pseudopotentials to the Projector Augmented-Wave Method. *Phys. Rev. B - Condens. Matter Mater. Phys.* **1999**, *59* (3), 1758–1775. https://doi.org/10.1103/PhysRevB.59.1758.

(5) Perdew, J. P.; Burke, K.; Ernzerhof, M. Generalized Gradient Approximation Made Simple. *Phys. Rev. Lett.* **1996**, *77* (18), 3865–3868. https://doi.org/10.1103/PhysRevLett.77.3865.

(6) Perdew, J. P.; Burke, K.; Ernzerhof, M. Generalized Gradient Approximation Made Simple. *Phys. Rev. Lett.* **1996**, *77* (18), 3865–3868. https://doi.org/10.1103/PhysRevLett.77.3865.

(7) Grimme, S. Accurate Description of van Der Waals Complexes by Density Functional Theory Including Empirical Corrections. *J. Comput. Chem.* **2004**, *25* (12), 1463–1473. https://doi.org/10.1002/jcc.20078.

(8) Grimme, S. Semiempirical GGA-Type Density Functional Constructed with a Long-Range Dispersion Correction. *J. Comput. Chem.* **2006**, *27* (15), 1787–1799. https://doi.org/https://doi.org/10.1002/jcc.20495.

(9) Rappé, A. K.; Casewit, C. J.; Colwell, K. S.; Goddard, W. A.; Skiff, W. M. UFF, a Full Periodic Table Force Field for Molecular Mechanics and Molecular Dynamics Simulations. *J. Am. Chem. Soc.* **1992**, *114* (25), 10024–10035. https://doi.org/10.1021/ja00051a040.

(10) Henkelman, G.; Arnaldsson, A.; Jónsson, H. A Fast and Robust Algorithm for Bader Decomposition of Charge Density. *Comput. Mater. Sci.* **2006**, *36* (3), 354–360. https://doi.org/10.1016/j.commatsci.2005.04.010.

(11) Allouche, A. Software News and Updates Gabedit — A Graphical User Interface for Computational Chemistry Softwares. *J. Comput. Chem.* **2012**, *32*, 174–182. https://doi.org/10.1002/jcc.

(12) Tang, W.; Sanville, E.; Henkelman, G. A Grid-Based Bader Analysis Algorithm without Lattice Bias. *J. Phys. Condens. Matter* **2009**, *21* (8). https://doi.org/10.1088/0953-8984/21/8/084204.

(13) Yu, M.; Trinkle, D. R. Accurate and Efficient Algorithm for Bader Charge Integration. *J. Chem. Phys.* **2011**, *134* (6), 1–8. https://doi.org/10.1063/1.3553716.

(14) Manz, T. A.; Limas, N. G. Introducing DDEC6 Atomic Population Analysis: Part 1. Charge Partitioning Theory and Methodology. *RSC Adv.* **2016**, *6* (53), 47771–47801. https://doi.org/10.1039/c6ra04656h.

(15) Manz, T. A.; Sholl, D. S. The Electrostatic Potential in Periodic and Nonperiodic Materials. *J. Chem. Theor. Comput.* **2010**, *6*, 2455–2468.

(16) Manz, T. A.; Sholl, D. S. Methods for Computing Accurate Atomic Spin Moments for Collinear and Noncollinear Magnetism in Periodic and Nonperiodic Materials. *J. Chem. Theory Comput.* **2011**, *7* (12), 4146–4164. https://doi.org/10.1021/ct200539n.

(17) Manz, T. A.; Sholl, D. S. Improved Atoms-in-Molecule Charge Partitioning Functional for Simultaneously Reproducing the Electrostatic Potential and Chemical States in Periodic and Nonperiodic Materials. *J. Chem. Theory Comput.* **2012**, *8* (8), 2844–2867. https://doi.org/10.1021/ct3002199.

(18) Manz, T. A. Introducing DDEC6 Atomic Population Analysis: Part 3. Comprehensive Method to Compute Bond Orders. *RSC Adv.* **2017**, *7* (72), 45552–45581. https://doi.org/10.1039/c7ra07400j.

(19) Gajdoš, M.; Hummer, K.; Kresse, G.; Furthmüller, J.; Bechstedt, F. Linear Optical Properties in the Projector-Augmented Wave Methodology. *Phys. Rev. B - Condens. Matter Mater. Phys.* **2006**, *73* (4), 1–9. https://doi.org/10.1103/PhysRevB.73.045112.

(20) Porezag, D.; Pederson, M. R. Infrared Intensities and Raman-Scattering Activities within Density-Functional Theory. *Phys. Rev. B - Condens. Matter Mater. Phys.* **1996**, *54* (11), 7830–7836. https://doi.org/10.1103/PhysRevB.54.7830.

(21) Baroni, S.; Resta, R. Ab Initio Calculation of the Macroscopic Dielectric Constant in Silicon. *Phys. Rev. B* **1986**, *33* (10), 7017–7021. https://doi.org/10.1103/PhysRevB.33.7017.

(22) Liu, W.-G.; Truhlar, D. G. Computational Linker Design for Highly Crystalline Metal–Organic Framework NU-1000. *Chem. Mater.* **2017**, *29* (19), 8073–8081. https://doi.org/10.1021/acs.chemmater.7b01624.

(23) Øien, S.; Wragg, D.; Reinsch, H.; Svelle, S.; Bordiga, S.; Lamberti, C.; Lillerud, K. P. Detailed Structure Analysis of Atomic Positions and Defects in Zirconium Metal-Organic Frameworks. *Cryst. Growth Des.* **2014**, *14* (11), 5370–5372. https://doi.org/10.1021/cg501386j.

(24) Yang, B.; Wheeler, J. I.; Sorensen, B.; Steagall, R.; Nielson, T.; Yao, J.; Mendez-Arroyo, J.; Ess, D. H. Computational Determination of Coordination Structure Impact on Adsorption and Acidity of Pristine and Sulfated MOF-808. *Mater. Adv.* **2021**, *2* (13), 4246–4254. https://doi.org/10.1039/d1ma00330e.

(25) Dymek, K.; Kurowski, G.; Kuterasiński, Ł.; Jędrzejczyk, R.; Szumera, M.; Sitarz, M.; Pajdak, A.; Kurach, Ł.; Boguszewska-Czubara, A.; Jodłowski, P. J. In Search of Effective UiO-66 Metal–Organic Frameworks for Artificial Kidney Application. *ACS Appl. Mater. Interfaces* **2021**, *13*, 45149–45160. https://doi.org/10.1021/acsami.1c05972.

(26) Jodłowski, P. J.; Dymek, K.; Kurowski, G.; Hyjek, K.; Boguszewska-Czubara, A.; Budzyńska, B.; Pajdak, A.; Kuterasiński, Ł.; Piskorz, W.; Jeleń, P.; Sitarz, M. In Vivo and in Vitro Studies of Efficient Mephedrone Adsorption over Zirconium-Based Metal-Organic Frameworks Corroborated by DFT+D Modeling. *Microporous Mesoporous Mater.* **2023**, *359* (February), 112647. https://doi.org/10.1016/j.micromeso.2023.112647.

(27) Berg, R. W.; Nørbygaard, T.; White, P. C.; Abdali, S. Ab Initio Calculations and Raman and SERS Spectral Analyses of Amphetamine Species. *Appl. Spectrosc. Rev.* **2011**, *46* (2), 107–131. https://doi.org/10.1080/05704928.2010.520180.

(28) Farquharson, S.; Brouillette, C.; Smith, W.; Shende, C. A Surface-Enhanced Raman Spectral Library of Important Drugs Associated With Point-of-Care and Field Applications. *Front. Chem.* **2019**, *7* (October), 1–16. https://doi.org/10.3389/fchem.2019.00706.

(29) Fagan, P.; Spálovská, D.; Kuchař, M.; Černohorský, T.; Komorousová, L.; Kocourková, L.; Setnička, V. Ecstasy Tablets: Rapid Identification and Determination of Enantiomeric Excess of MDMA. *Forensic Chem.* **2021**, *26* (September), 100381. https://doi.org/10.1016/j.forc.2021.100381.

(30) de Oliveira Penido, C. A. F.; Pacheco, M. T. T.; Lednev, I. K.; Silveira, L. Raman Spectroscopy in Forensic Analysis: Identification of Cocaine and Other Illegal Drugs of Abuse. *J. Raman Spectrosc.* **2016**, *47* (1), 28–38. https://doi.org/10.1002/jrs.4864.

(31) Jodłowski, P. J.; Dymek, K.; Kurowski, G.; Jaśkowska, J.; Bury, W.; Pander, M.; Wnorowska, S.; Targowska-Duda, K.; Piskorz, W.; Wnorowski, A.; Boguszewska-

Czubara, A. Zirconium-Based Metal–Organic Frameworks as Acriflavine Cargos in the Battle against Coronaviruses─A Theoretical and Experimental Approach. *ACS Appl. Mater. Interfaces* **2022**, *14* (25), 28615–28627. https://doi.org/10.1021/acsami.2c06420.
